# No Transition Metals Required – Oxygen Promoted Synthesis of Imines from Primary Alcohols and Amines under Ambient Conditions

**DOI:** 10.1002/chem.202300094

**Published:** 2023-04-11

**Authors:** Daniel Himmelbauer, Radu Talmazan, Stefan Weber, Jan Pecak, Antonio Thun‐Hohenstein, Maxine‐Sophie Geissler, Lukas Pachmann, Marc Pignitter, Maren Podewitz, Karl Kirchner

**Affiliations:** ^1^ Institute of Applied Synthetic Chemistry TU Wien Getreidemarkt 9/163-AC A-1060 Wien Austria; ^2^ Institute of Materials Chemistry TU Wien Getreidemarkt 9 A-1060 Wien Austria; ^3^ Department of Physiological Chemistry Faculty of Chemistry University of Vienna Althanstrasse 14 1090 Wien Austria

**Keywords:** DFT, imines, oxidation reactions, radical mechanisms, transition metal free

## Abstract

The synthesis of imines denotes a cornerstone in organic chemistry. The use of alcohols as renewable substituents for carbonyl‐functionality represents an attractive opportunity. Consequently, carbonyl moieties can be in situ generated from alcohols upon transition‐metal catalysis under inert atmosphere. Alternatively, bases can be utilized under aerobic conditions. In this context, we report the synthesis of imines from benzyl alcohols and anilines, promoted by KO^t^Bu under aerobic conditions at room temperature, in the absence of any transition‐metal catalyst. A detailed investigation of the radical mechanism of the underlying reaction is presented. This reveals a complex reaction network fully supporting the experimental findings.

## Graphical abstract

**Transition‐metal free**: The synthesis of imines from benzyl alcohol and anilines promoted by oxygen and KO^t^Bu at room temperature in the absence of a transition metal catalyst is described. The detailed investigation of the radical mechanism is presented revealing complex reaction paths and branching patterns.

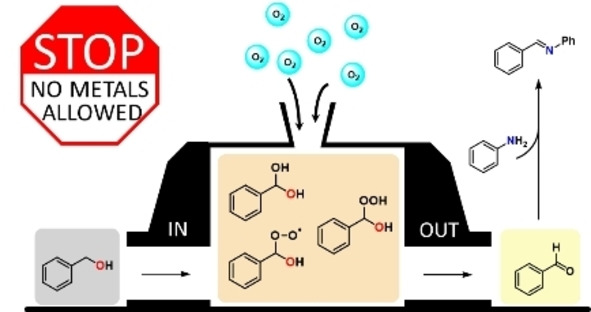

## Introduction

**Scheme 1:**
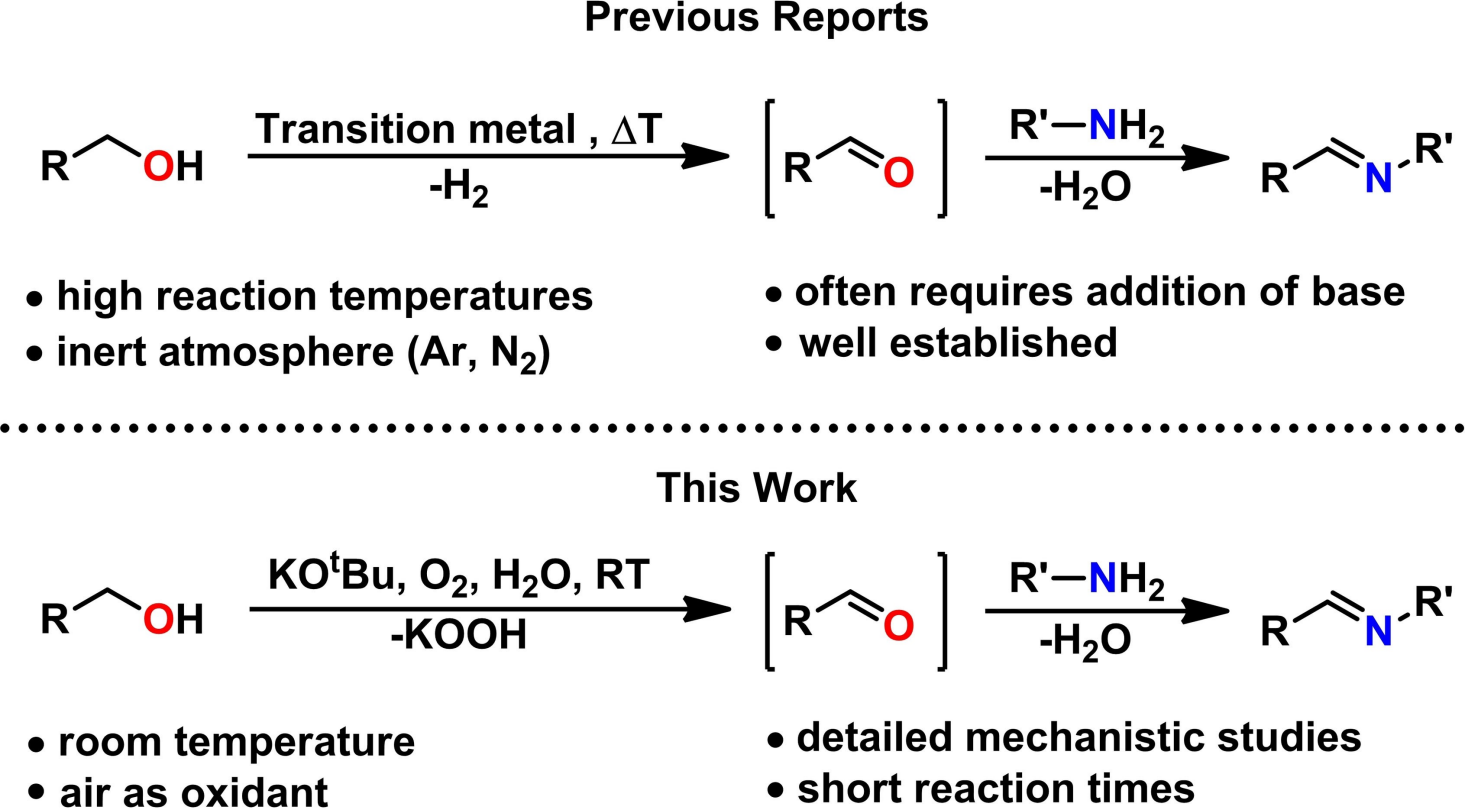
Transition metal catalyzed synthesis of imines from alcohols and amines (top) and base/oxygen promoted imine synthesis in the absence of a transition‐metal catalyst (bottom).

The utilization of alcohols as alkylation surrogates for aldehydes represents a highly desirable reaction route in organic chemistry, since alcohols can be directly obtained from biomass.^[1]^ Therefore, efficient catalytic procedures for in situ generation of carbonyl moieties, based on noble‐^[2]^ and non‐precious transition metals^[3]^ were described in the last decades by a manifold of researchers, including reports from our group.^[4]^ However, it has been shown that benzyl alcohols can be swiftly oxidized to the corresponding carbonyls upon treatment with a strong base under atmospheric conditions.^[5]^ In fact, various condensation reactions^[6]^ can be carried out with benzyl alcohols in presence of base (sometimes in catalytic amounts) without transition‐metal complexes. It was found that the majority of these transformations are driven by alkali metal catalyzed Meerwein–Ponndorf–Verley reduction/Oppenauer oxidation (MPV‐O) reactions.^[7]^ A similar transformation represents the synthesis of imines from benzyl alcohols and anilines^[8]^ – a reaction that has been known since the 1950s but seems to be largely overseen in the organometallic's community (Scheme 1). Additionally, Le Berre and co‐workers studied the oxidation of the related diphenylmethanol with KO^t^Bu and oxygen to benzophenone and KOOH, originally reported as KO_2_.^[9]^

In contrast to transition metal catalyzed reactions, only little mechanistic insights are provided so far for this process. We describe here an efficient coupling of alcohols and amines promoted by oxygen at room temperature and propose a detailed reaction mechanism based on both experimental findings and extensive theoretical studies.

## Results and Discussion

In order to examine the impact of various parameters in oxygen‐promoted imine formation, optimization reactions were carried out, utilizing benzyl alcohol and aniline as model system. Selected results are depicted in Table 1. At first, different bases were investigated, showing good reactivity for strong bases. In this context, quantitative product formation could be observed for KO^t^Bu (Table 1, entry 1) at room temperature in toluene. Notably, under the same reaction conditions, only traces of product were formed with KOH. Concerning the solvent, high conversions were detected in toluene and THF. It should be noted that poor reactivity was observed, if the reaction was carried out under argon atmosphere and under UV‐irradiation (Table 1, entries 8 and 9). Interestingly, TEMPO can be used as efficient promotor under oxygen‐free conditions (Table 1, entry 10). Having established the optimal parameters, scope and limitation of the introduced protocol was explored. In this context, anilines, containing electron‐donating or ‐withdrawing groups, were reacted with benzyl alcohol to give excellent yields (Table 2 entries **1 a**–**1 r**). Furthermore, the condensation of substituted benzyl alcohols with anilines was examined (Table 2 entries **1 s**–**1 ah**), showing high reactivity for most substrates. The introduced protocol tolerates a wide range of functional groups including halides, (thio)ethers, alkenes, acetals, the nitro‐group as well as electron‐rich or electron‐poor heterocycles. However, a massive drop in reactivity was observed for the reaction of aniline with aliphatic 1‐octanol (see Table 2 entry **1 ai**).

**Table 1:** Optimization reaction for oxygen‐promoted imine formation.^[a]^

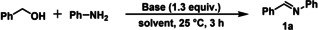			
Entry	Base	Solvent	Conversion^[b]^ [%]
**1**	**KO^t^Bu**	**toluene**	**>99**
2	KOH	toluene	traces
3	KHMDS	toluene	66
4	NaO^t^Bu	toluene	50
5	LiO^t^Bu	toluene	60
6	KO^t^Bu	THF	>99
7	KO^t^Bu	MeCN	11
8^[c]^	KO^t^Bu	toluene	5
9^[c,d]^	KO^t^Bu	toluene	19
10^[c,e]^	KO^t^Bu	toluene	82

[a] Conditions: benzyl alcohol (1.0 mmol), aniline (1.2 mmol), base (1.3 mmol), solvent (6 mL), 25 °C, open reaction vessel, 3 h. [b] determined by GC‐MS. [c] under Ar. [d] under UV‐irradiation. [e] 1 equiv. TEMPO added.

**Table 2:**
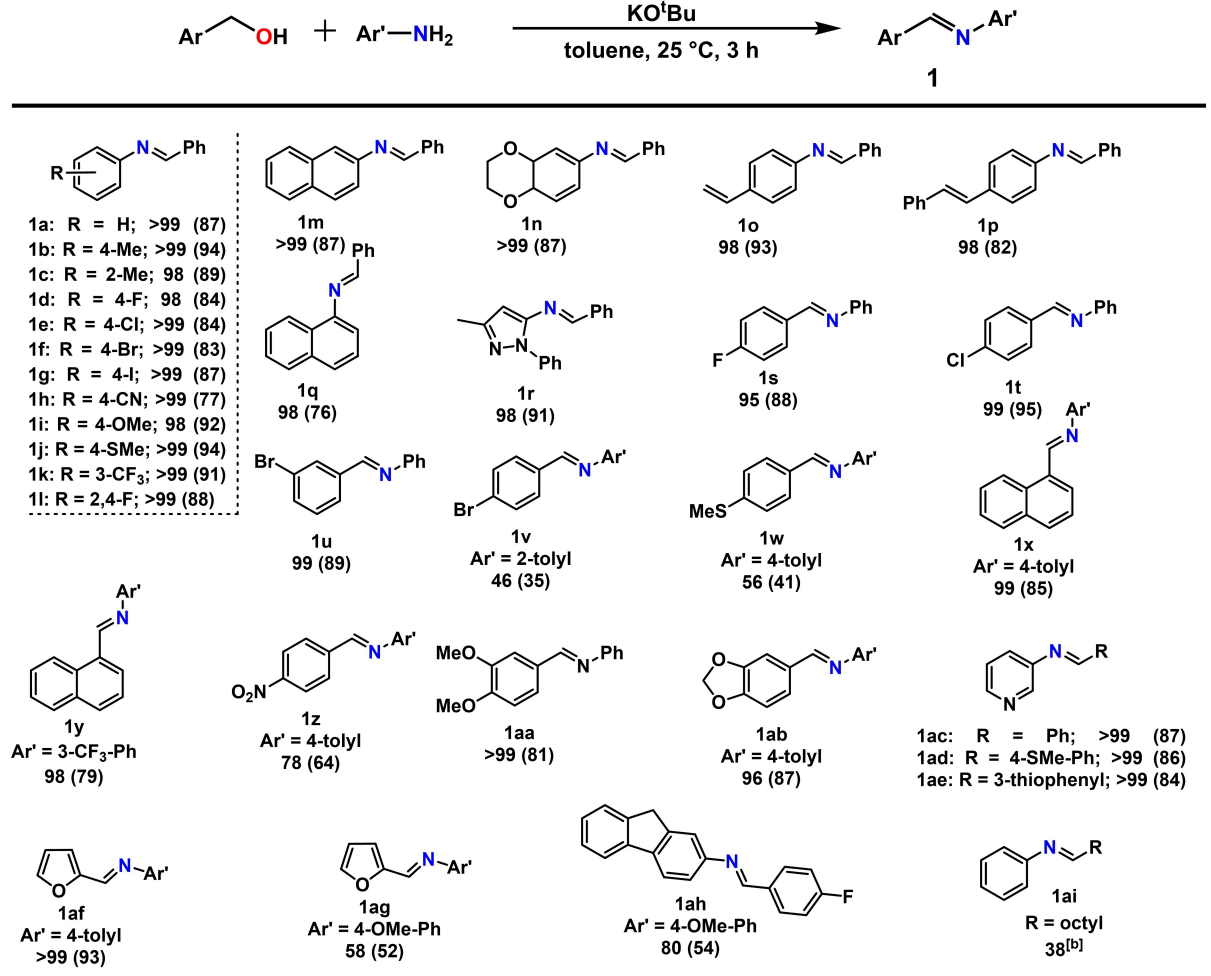
Substrate scope of oxygen‐promoted imine formation.^[a]^

[a] Conditions: benzyl alcohol (1.0 mmol), aniline (1.2 mmol), KO^t^Bu (1.3 mmol), solvent (6 mL), 25 °C, open reaction vessel, 3 h, conversion determined by GC‐MS, isolated yield given in parenthesis. [b] 24 h.

Encouraged by the high reactivity of the developed methodology we performed mechanistic investigations, employing benzyl alcohol and aniline as model substrates for all experimental and computational studies. At first, the role of KO^t^Bu was explored. It should be noted that the purity of KO^t^Bu and several other substrates was carefully analyzed to exclude traces of potentially (catalytically) active impurities using ICP‐MS. We could reproduce all results with various batches of KO^t^Bu from different suppliers with purity levels from 98 % up to >99.99 %. Furthermore, upon stochiometric addition of 18‐crown‐6 as potassium scavenger 88 % conversion was detected: This suggests that the metal ion is not involved in the rate‐determining step, ruling out an alkali metal MPV‐O pathway (cf. computational investigation). Next, we investigated whether this transformation is driven by a radical mechanism. Although the addition of TEMPO did not lead to a drop in reactivity, the reaction pathway clearly involves radicals, since the addition of the typical radical trap 3,4‐chromanediol completely suppressed all reactivity. KO^t^Bu seems to simply act as a base, forming the benzylate salt, which then forms a radical with oxygen from the air (see below). In fact, potassium benzylate, PhCH_2_OK, can be used as starting material, resulting in moderate yield due to high moisture sensitivity of the salt.

**Scheme 2:**
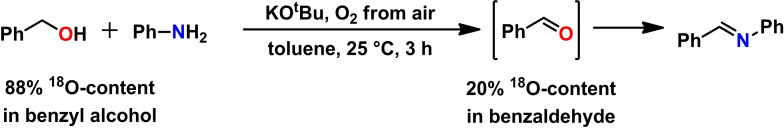
^18^O‐Labeling experiment for the base/oxygen promoted synthesis of imines.

The impact of oxygen on the reaction was investigated and full conversion was observed for the model reaction within 15 min under a pure O_2_ atmosphere. Next, deuterated benzyl alcohol‐α,α‐D_2_ was used as substrate. No drop of deuterium content was detected in the product. Surprisingly, if ^18^O‐enriched benzyl alcohol was employed as starting material, a massive drop in ^18^O‐content was observed in the in situ generated benzaldehyde (Scheme 2). This could be attributed to an exchange with oxygen from air or to a scrambling reaction with water (cf. computational investigation).

Furthermore, we examined whether catalytic amounts of KO^t^Bu are sufficient for this transformation as described by several researchers under more forcing conditions.^[8]^ If 30 mol % of KO^t^Bu are used in the model reaction under O_2_‐atmosphere, a turnover number of approx. 2.2 was observed, which proves that the reaction can in general be operated in a catalytic fashion.

**Scheme 3:**
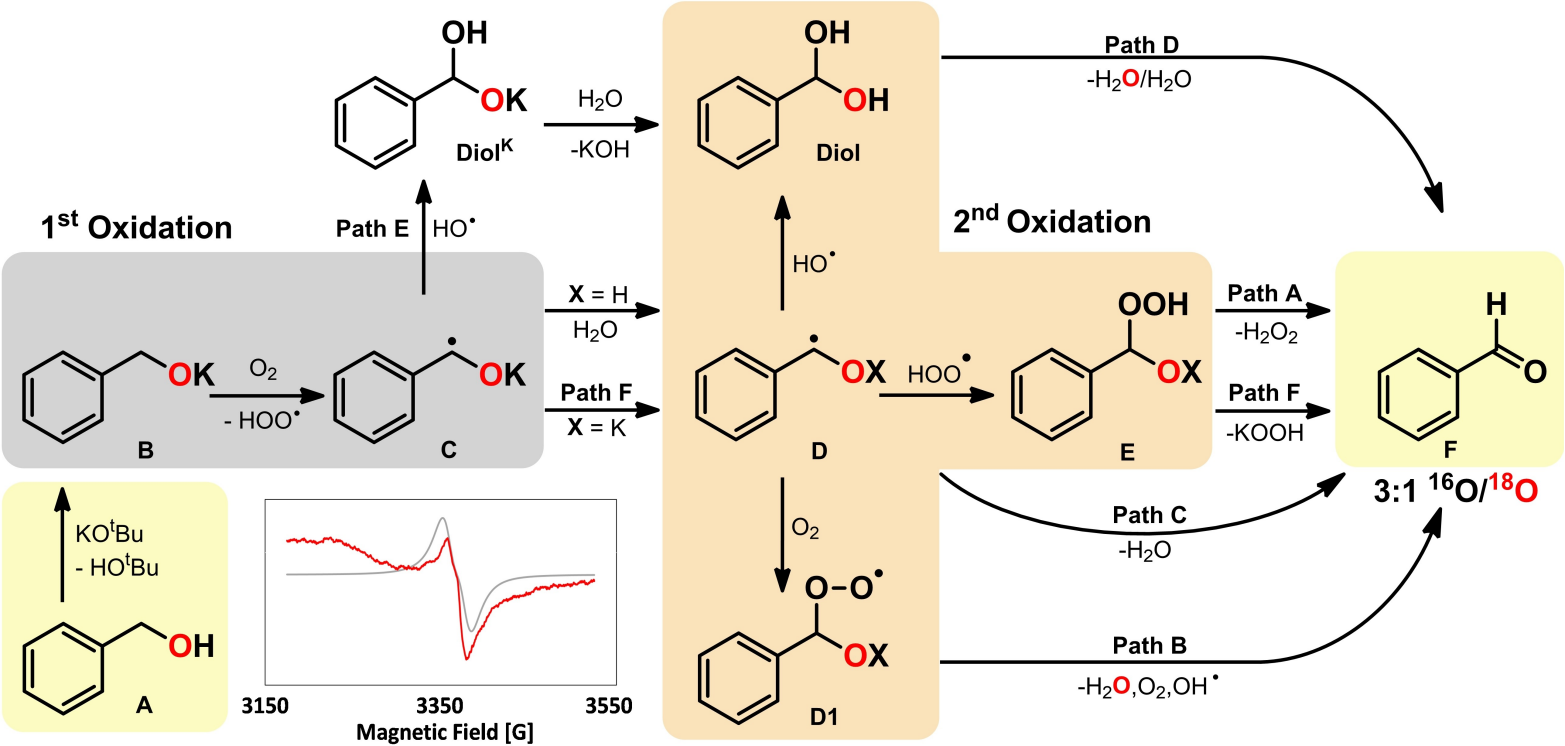
Simplified reaction mechanism for the formation of benzaldehyde from benzyl alcohol through various pathways. The red O‐atoms represent the ^18^O‐labeling. The inserted EPR spectrum depicts a radical species (**C** or **D**) observed in experiment.

To gain further insights into the reaction mechanism, we carried out extensive quantum chemical calculations (DLPNO‐CCSD(T)/def2‐QZVP//PBE0/def2‐TZVP/D4 in toluene,^[10]^ see Supporting Information for details), through which we were able to determine a complex radical pathway. Formation of the benzyl aldehyde can be achieved with and without loss in ^18^O‐labeling, shown in Scheme 3. The reaction mechanism can be separated into several reaction paths starting from path A, which can branch off at various reaction steps resulting in pathways (paths B to F) that depend on intermediates generated in preceding reaction steps. As some of the intermediates are rather short lived, we refrain from quantifying the likelihood of these pathways to occur. Overall, the reaction mechanism can be subdivided into two subsequent hydrogen atom abstraction (HAT) steps.

**Figure 1:**
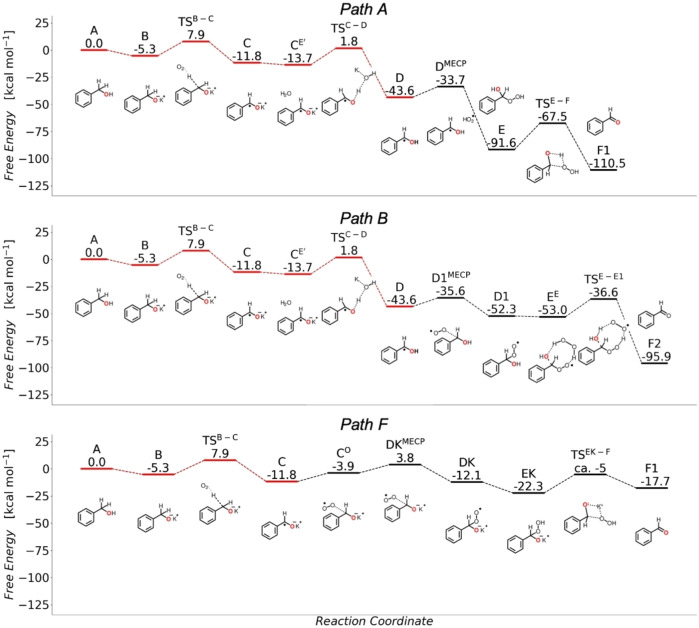
Simplified energy profiles of the various radical reaction pathways to form benzaldehyde. Paths A and B are water assisted, while path F denotes the water‐free pathway. The value for the transition state **TS^EK−F^** is only an estimate, based on reaction path optimization. All values are free energies Δ*G*, in kcal/mol, calculated with DLPNO‐CCSD(T)/def2‐QZVP//PBE0/def2‐TZVP/D4. The ^18^O‐label is depicted in red. Due to the nature of CCSD, accurate energies are not achievable for the MECP points. In there cases, the energy differences are obtained from B2PLYP/def2‐TZVP/D4//PBE0/def2‐TZVP/D4 (for more information see computational methodology section and SI).

From the simplified reaction free energy diagram presented in Figure 1 showing relevant intermediates and transition states (for full diagram see Supporting Information Figures S7 and S8), it can be seen that benzyl alcohol **A** reacts with the tert‐butyl base to form the potassium salt **B**. This step of the reaction is favored, with a Δ*G*=−5.3 kcal/mol. Notably, this step can itself be skipped completely as the reaction was experimentally shown to proceed when starting directly from the potassium salt. This also indicates that the tert‐butyl base does not play a significant role in reaction steps following **B**.

The salt proceeds to interact with molecular oxygen, which leads to oxidation and the formation of the radical **C** via hydrogen atom abstraction (HAT) depicted in **TS^B−C^**. The free energy barrier for this step is Δ*G* ^≠^=13.2 kcal/mol, while the formation of the **C** radical itself is favored (Δ*G*=−11.8 kcal/mol). With the formation of **C**, a rather stable peroxyl radical is liberated. In presence of residual water, the **C^E’^** complex (Δ*G*=−13.7 kcal/mol) is formed. The potassium ion assists proton abstraction from water, forming the radical **D**, which is strongly favored, with Δ*G*=−43.6 kcal/mol. The energy barrier for this step is given by **TS^C−D^** and has a free energy of Δ*G* ^≠^=15.5 kcal/mol. For both **C** and **D** g_iso_ values were calculated,^[11]^ with g_iso,C_=2.003 and g_iso,D_=2.004. A radical species was also detected in EPR spectroscopy (g_iso_=2.001, see Scheme 3) and our g‐tensor calculations indicate that this structure must be either **C** or **D** or a mixture of both (see Figure S9 for details).

In path A, the initiation of the reaction, **D** can undergo a spin crossing event, in the proximity of the peroxyl radical generated previously, with an energy barrier of Δ*G* ^≠^=9.9 kcal/mol for **D^MECP^**, resulting in the formation of **E** (Δ*G*=−91.6 kcal/mol). Such hydroperoxide species are found to be stable for similar compounds.^[12]^ **E** then undergoes a scrambling reaction with an energy barrier of Δ*G* ^≠^=24.1 kcal/mol, through **TS^E−F^**, resulting in the final product, **F1**. In path A, the initial ^18^O‐labeled oxygen of the benzyl alcohol is retained.

From structure **D**, the reaction can also proceed in the presence of environmental O_2_. Following path B (see Figure 1) the reaction with oxygen results in a spin crossover event, with a barrier of Δ*G* ^≠^=8.0 kcal/mol for **D1^MECP^**, culminating in the formation of **D1** (with Δ*G*=−52.3 kcal/mol). **D1** can then re‐enter path A, propagating the radical reaction by HAT from **B** under generation of **E** with a free energy of this reaction step of Δ*G*=−21.9 kcal/mol (not shown in diagram). Alternatively, **D1** can proceed via **E^E^** (Δ*G*=−53.0 kcal/mol), provided that the H_2_O_2_ generated during the path A is in proximity. Continuing from **E^E^**, the structure undergoes a series of rearrangements, with the highest energy point represented by **TS^E−E1^**, at Δ*G*=−36.6 kcal/mol. The rate determining barrier for these steps is Δ*G* ^≠^=16.4 kcal/mol, the second HAT. In the final product of the reaction, **F2**, the ^18^O‐labeled oxygen atom is substituted.

Moreover, in the presence of a hydroxyl radical (generated in path B), the additional path C and path D branching off from **D** are also possible (see Figure S8). Depending on the orientation of the two radicals in the encounter structure, path C can be followed, through the **F1^MECP^** spin crossover point (second HAT). This presents a barrier of Δ*G* ^≠^=10.8 kcal/mol and results in the **F1** product, as it transitions into the singlet spin state. Alternatively (path D), a different orientation of the hydroxyl radical pushes the structure into forming a diol. The spin transition barrier for **Diol^MECP^** is Δ*G* ^≠^=11.5 kcal/mol. Once on the singlet surface, **Diol** is created (Δ*G*=−36.7 kcal/mol). This is subsequently followed by a scrambling reaction with a barrier of Δ*G* ^≠^=18.5 kcal/mol, that has an equal probability of resulting in **F1** (labelled benzaldehyde) or **F2** (non‐labelled benzaldehyde). The cumulative barrier for this path is Δ*G* ^≠^=25.4 kcal/mol.

Another possibility for branching exists in **C** (path E), provided that an OH radical is present (see Figure S8). The spin crossover **C^MECP^** that takes place has a barrier of Δ*G* ^≠^=15.1 kcal/mol and results in **Diol^K^** (Δ*G*=−36.1 kcal/mol), the K^+^ salt analogue of **Diol**. This structure can then react in the presence of water to result in the formation of **Diol** with a barrier for this reaction step of Δ*G* ^≠^=17.2 kcal/mol (**TS^C−Diol^**). Afterwards the **Diol** intermediate re‐enters path D via **TS^Diol−F^** (scrambling) to yield **F1** and **F2** with equal probability.

Many alternate paths towards the product **F** starting from each intermediate were attempted, but the transition states were found to be either very high or calculations converged to previously known intermediates. In our proposed mechanism water plays a crucial role to form (neutral) low energy intermediates and transition states. In contrast, the initial investigation by Le Berre hinted at a water‐free reaction but was missing mechanistic details.^[9]^ Nevertheless, we modelled this pathway in analogy to our hitherto determined mechanism. In this reaction cascade (path F), **C** interacts with O_2_ forming the potassium analogue of **D1**, denoted as **DK**_,_ which is with Δ*G*=−12.1 kcal/mol significantly less stable than the potassium free counterpart **D1** (Δ*G*=−52.3 kcal/mol). Likewise, the barrier for this transformation was estimated to be around 16 kcal/mol compared to Δ*G* ^≠^=13.6 kcal/mol for the water‐assisted pathway. Radical propagation mediated by **DK** resulting in **EK** is also less favorable than in the water‐assisted pathway (Δ*G*=−10.2 kcal/mol vs. Δ*G*=−21.9 kcal/mol), while the final formation of benzaldehyde is even exergonic with Δ*G*=+4.6 kcal/mol. The relative stability of **F** in the water‐free pathway is thereby only Δ*G*=−17.7 kcal/mol as opposed to Δ*G*=−110.5 kcal/mol in the water‐assisted pathway. Although the water‐free Le Berre mechanism is feasible, it is thermodynamically less favorable. In addition, we would like to note here that the consecutive reaction with amine leads to stochiometric amounts of water.

The termination of the reaction can happen via paths C, D or E, as well as through the decomposition of H_2_O_2_. This can be generated in path A, or by the combination of two OH radicals formed via path B.

A quantitative estimation for the ratio of **F1** to **F2** is very challenging, because several of the pathways depend on the presence and proximity of short‐lived radical species. From a kinetic perspective, path B presents the lowest overall barriers, Δ*G* ^≠^_max_=16.4 kcal/mol, which suggest that given the right environment, **F2** would be produced in higher amounts – a finding in line with experimental observations. Still, this reaction pathway is kinetically competing with the water‐free pathway, for which a rate‐determining barrier of ca. 17 kcal/mol is estimated.

The fast condensation to the final imine, from the amine and the in situ generated benzaldehyde proceeds via a well‐known exergonic C−N coupling pathway with a reaction barrier of Δ*G* ^≠^=16 kcal/mol.^[13]^ The released water promotes the main water‐assisted reaction pathway – accelerating benzaldehyde formation. The experimentally determined drop in ^18^O‐content can therefore originate from exchange with oxygen (path B) or from scrambling with water (paths D and E). Alternatively, ^18^O‐labeled benzaldehyde can undergo base mediated exchange with water, as known from fundamental organic chemistry. However, this exchange has to compete with the rapid imine formation.

To determine the extent to which each reaction pathway is followed, an exhaustive microkinetic modelling would have to be performed, which is beyond the scope of this paper.

In conclusion, we report the first concise reaction mechanism of the transition metal free in situ formation of benzaldehyde under ambient conditions. Our combined experimental and computational studies unambiguously showed that dioxygen (and water) is essential for this complex radical process. We could show that KO^t^Bu acts solely as a deprotonating agent, that the concentration of O_2_ is critical for a rapid conversion and that the presence of a transition metal catalyst is not required.

The overall mechanism can be decomposed into two separate HAT reactions that eventually lead to oxidation of benzyl alcohol to benzaldehyde. EPR spectroscopy was able to confirm the presence of a radical intermediate and corroborate theoretical calculations. The forgoing of expensive transition‐metal catalysts in the formation of aldehydes from alcohols is highly desirable. Thus, the detailed mechanism provides starting points for future investigations and optimizations of this and similar systems.

## Experimental Section

**General protocol for N‐Alkylation**: KO^t^Bu (146 mg, 1.3 mmol) was put into a 10 mL flat‐bottom vial. Then a toluene (6 mL) solution of the aniline derivative (110 μL, 1.2 mmol) and alcohol (104 μL, 1.0 mmol) was added. The reaction mixture was stirred for 3 h at room temperature, while exposed to ambient atmospheric conditions. A sample was taken for GC‐MS analysis. Afterwards, the reaction mixture was filtered over a pad of Celite, and the solvent was removed under reduced pressure to yield the crude product which was purified via recrystallization or column chromatography.

**Computational methodology**: Structures were fully optimized using the PBE0 density functional,^[10e]^ the def2‐TZVP basis set,^[10d]^ D4 corrections.^[10f]^ Calculations were performed in toluene, modelled as implicit solvent.^[10g]^ Structures were verified to energy minima or transition states by analysis of the Hessian, showing either no or exactly one imaginary frequency that matches the reaction coordinate, respectively. Final electronic energies were calculated with local pair‐natural orbital (DLPNO) based singles‐ and doubles, and perturbative triples coupled cluster theory (CCSD(T)),^[10a]^ while thermodynamic corrections to obtain free energies were calculated with DFT (see above) using the rigid‐rotor quasi harmonic oscillator model. Minimum energy crossing points were carried out with a method proposed by Harvey.^[14]^ EPR tensors were calculated with the double‐hybrid density functional B2PLYP^[15]^ and gauge‐invariant independent atomic orbitals, while spectra were visualized with Easyspin.^[16]^ All calculations were performed with ORCA 5.0.3.^[17]^ Subsequent to the completion of this work, it was made known that ORCA 5.0.3 contained a bug in the implementation of D4, which could occasionally lead to wrong geometries and energies. As this was fixed in orca 5.0.4, we have re‐optimized the structures and computed the corresponding DFT energies where applicable. There were no notable discrepancies between the two ORCA versions, in the case of our system.

## Conflict of interest

The authors declare no conflict of interest.

1

## Supporting information

As a service to our authors and readers, this journal provides supporting information supplied by the authors. Such materials are peer reviewed and may be re‐organized for online delivery, but are not copy‐edited or typeset. Technical support issues arising from supporting information (other than missing files) should be addressed to the authors.

Supporting Information

## Data Availability

The data that support the findings of this study are available. In the supplementary material of this article, the coordinates of all calculated structures are available in xmol format under https://doi.org/10.5281/zenodo.7626699.

## References

[1a] G. Guillena , D. J. Ramon , M. Yus , Chem. Rev. 2010, 110, 1611–1641; 19928825 10.1021/cr9002159

[1b] K. Barta , P. C. Ford , Acc. Chem. Res. 2014, 47, 1503–1512. 24745655 10.1021/ar4002894

[2a] B. Gnanaprakasam , J. Zhang , D. Milstein , Angew. Chem. Int. Ed. 2010, 49, 1468–1471; 10.1002/anie.200907018 20101664

[2b] D. Srimani , Y. Ben-David , D. Milstein , Angew. Chem. Int. Ed. 2013, 52, 4012–4015; 10.1002/anie.201300574 23468418

[2c] S. Imm , S. Bähn , L. Neubert , H. Neumann , M. Beller , Angew. Chem. Int. Ed. 2010, 49, 8126–8129; 10.1002/anie.201002576 20677295

[2d] M. H. S. A. Hamid , J. M. J. Williams , Chem. Commun. 2007, 725–727; 10.1039/b616859k 17392963

[2e] T. Zweifel , J.-V. Naubron , H. Grützmacher , Angew. Chem. Int. Ed. 2009, 48, 559–563; 10.1002/anie.200804757 19072802

[2f] R. Kawahara , K. Fujita , R. Yamaguchi , J. Am. Chem. Soc. 2010, 132, 15108–15111; 20931960 10.1021/ja107274w

[2g] B. Blank , S. Michlik , R. Kempe , Adv. Synth. Catal. 2009, 351, 2903–2911;

[2h] M. A. Esteruelas , V. Lezáun , A. Martínez , M. Oliván , E. Oñate , Organometallics 2017, 36, 2996–3004.

[3a] G. Zhang , S. K. Hanson , Org. Lett. 2013, 15, 650–653; 23311959 10.1021/ol303479f

[3b] K. Paudel , S. Xu , O. Hietsoi , B. Pandey , C. Onuh , K. Ding , Organometallics 2021, 40, 418–426;

[3c] T. Yan , B. L. T. Feringa , K. Barta , Nat. Commun. 2014, 5, 5602–5609; 25424885 10.1038/ncomms6602

[3d] A. J. Rawlings , L. J. Diorazio , M. Wills , Org. Lett. 2015, 17, 1086–1089; 25687336 10.1021/ol503587n

[3e] A. Mukherjee , A. Nerush , G. Leitus , L. J. W. Shimon , Y. Ben-David , N. A. Espinosa Jalapa , D. Milstein , J. Am. Chem. Soc. 2016, 138, 4298–4301; 26924231 10.1021/jacs.5b13519

[3f] R. Fertig , T. Irrgang , F. Freitag , J. Zander , R. Kempe , ACS Catal. 2018, 8, 8525–8530.

[4a] M. Mastalir , G. Tomsu , E. Pittenauer , G. Allmaier , K. Kirchner , Org. Lett. 2016, 18, 3462–3465; 27356282 10.1021/acs.orglett.6b01647

[4b] M. Mastalir , M. Glatz , N. Gorgas , B. Stöger , E. Pittenauer , G. Allmaier , L. F. Veiros , K. Kirchner , Chem. Eur. J. 2016, 22, 12316–12320; 27377955 10.1002/chem.201603148

[4c] M. Mastalir , M. Glatz , E. Pittenauer , G. Allmaier , K. Kirchner , J. Am. Chem. Soc. 2016, 138, 15543–15546; 27934004 10.1021/jacs.6b10433

[4d] M. Mastalir , M. Glatz , E. Pittenauer , G. Allmaier , K. Kirchner , J. Am. Chem. Soc. 2017, 139, 8812–8815. 28628321 10.1021/jacs.7b05253

[5a] G. A. Russell , E. G. Janzen , J. Am. Chem. Soc. 1962, 84, 4153–4154;

[5b] X. Wang , D. Z. Wang , Tetrahedron 2011, 67, 3406–341;

[5c] L.-H. Zhou , X.-Q. Yu , L. Pu , Tetrahedron Lett. 2010, 51, 475–477;

[5d] W. Zhang , M. Liu , H. Wu , J. Ding , J. Cheng , Tetrahedron Lett. 2008, 5336–5338.

[6a] L. J. Allen , R. H. Crabtree , Green Chem. 2010, 12, 1362–1364;

[6b] Q. Xu , J. Chen , Q. Liu , Adv. Synth. Catal. 2013, 355, 697–704;

[6c] Q. Xu , J. Chen , H. Tian , X. Yuan , S. Li , C. Zhou , J. Liu , Angew. Chem. Int. Ed. 2014, 53, 225–229; 10.1002/anie.201308642 24273002

[6d] N. Shao , J. Rodriguez , A. Quintard , Org. Lett. 2020, 22, 7197–7201; 32877190 10.1021/acs.orglett.0c02536

[6e] C.-Z. Yao , Q.-Q. Li , M.-M. Wang , X.-S. Ning , Y.-B. Kang , Chem. Commun. 2015, 51, 7729–7732; 10.1039/c5cc01965f 25850736

[6f] B. C. Roy , I. A. Ansari , S. A. Samim , S. Kundu , Chem. Asian J. 2019, 14, 2215–2219; 31046180 10.1002/asia.201900285

[6g] M. Xiao , X. Yue , R. Xu , W. Tang , D. Xue , C. Li , M. Lei , J. Xiao , C. Wang , Angew. Chem. Int. Ed. 2019, 58, 10528–10536; 10.1002/anie.201905870 31162782

[6h] Q. Xu , Q. Li , X. Zhu , J. Chen , Adv. Snth. Catal. 2013, 355, 73–80;

[6i] C. Wang , C. Chen , J. Han , J. Zhang , Y. Yao , Y. Zhao , Eur. J. Org. Chem. 2015, 2972–2977;

[6j] Q.-Q. Li , Z.-F. Xiao , C.-Z. Yao , H.-X. Zheng , Y.-B. Kang , Org. Lett. 2015, 17, 5328–5331; 26473336 10.1021/acs.orglett.5b02685

[6k] X.-H. Lu , Y.-W. Sun , X.-L. Wie , C. Peng , D. Zhou , Q.-H. Xia , Catal. Commun. 2014, 55, 78–82;

[6l] X. Li , S. Li , Q. Li , X. Dong , Y. Li , X. Yu , Q. Xu , Tetrahedron 2016, 72, 264–272;

[6m] S. Tivari , P. K. Singh , P. P. Singh , V. Srivastava , RSC Adv. 2022, 12, 35221–35226. 36540212 10.1039/d2ra07065k PMC9730743

[7a] V. Polshettiwar , R. S. Varma , Green Chem. 2009, 11, 1313–1316;

[7b] A. Ouali , J.-P. Majoral , A.-M. Caminade , M. Taillefer , ChemCatChem 2009, 1, 504–509;

[7c] J. Ballester , A.-M. Caminade , J.-P. Majoral , M. Taillefer , A. Ouali , Catal. Commun. 2014, 47, 58–62.

[8a] Y. Sprinzak , J. Am. Chem. Soc. 1956, 78, 3207–3208;

[8b] J. Xu , R. Zhuang , L. Bao , G. Tang , Y. Zhao , Green Chem. 2012, 14, 2384–2387;

[8c] R. R. Donthiri , R. D. Patil , S. Adimurthy , Eur. J. Org. Chem. 2012, 4457–4460.

[9] A. Leberre , Bull. Soc. Chim. Fr. 1961, 1543–1549.

[10a] C. Riplinger , F. Neese , J. Chem. Phys. 2013, 138, 034106; 23343267 10.1063/1.4773581

[10b] F. Weigend , R. Ahlrichs , Phys. Chem. Chem. Phys. 2005, 7, 3297–3305; 16240044 10.1039/b508541a

[10c] J. Zheng , X. Xu , D. G. Truhlar , Theor. Chem. Acc. 2011, 128, 295–305;

[10d] F. Weigend , Phys. Chem. Chem. Phys. 2006, 8, 1057–1065; 16633586 10.1039/b515623h

[10e] C. Adamo , V. Barone , J. Chem. Phys. 1999, 110, 6158–6170;

[10f] E. Caldeweyher , C. Bannwarth , S. Grimme , J. Chem. Phys. 2017, 147, 034112; 28734285 10.1063/1.4993215

[10g] V. Barone , M. Cossi , J. Phys. Chem. A 1998, 102, 1995–2001.

[11a] A. Hellweg , C. Hattig , S. Hofener , W. Klopper , Theor. Chem. Acc. 2007, 117, 587–597;

[11b] G. L. Stoychev , A. A. Auer , F. Neese , J. Chem. Theory Comput. 2018, 14, 4756–4771; 30048136 10.1021/acs.jctc.8b00624

[11c] V. A. Tran , F. Neese , J. Chem. Phys. 2020, 153, 054105. 32770923 10.1063/5.0013799

[12] J. M. Anglada , R. Crehuet , J. S. Francisco , Eur. J. Chem. 2016, 22, 18092–18100. 10.1002/chem.201604499 27808436

[13] Y. Q. Ding , Y. Z. Cui , T. D. Li , J. Phys. Chem. A 2015, 119, 4252–4260 . 25859816 10.1021/acs.jpca.5b02186

[14] J. N. Harvey , M. Aschi , H. Schwarz , W. Koch Theor , Chem. Acc. 1998, 99, 95.

[15] S. Grimme , J. Chem. Phys. 2006, 124, 34108. 10.1063/1.2148954 16438568

[16] S. Stoll , A. Schweiger , J. Magn. Reson. 2006, 178, 42–55. 16188474 10.1016/j.jmr.2005.08.013

[17a] F. Neese , WIREs Comput. Mol. Sci. 2022,12, e1606;

[17b] F. Neese , F. Wennemohs , U. Becker , C. Riplinger , J. Chem. Phys. 2020, 152, 224108. 32534543 10.1063/5.0004608

