# Supplementary material for: No Transition Metals Required – Oxygen Promoted Synthesis of Imines from Primary Alcohols and Amines under Ambient Conditions
Source: Chemistry. 2023 Apr 11;29(29):e202300094. doi: 10.1002/chem.202300094 (PMC10946877; doi:10.1002/chem.202300094)
Supplement: Supplementary file 1 — Supporting Information [file CHEM-29-0-s001.pdf]

Chemistry–A European Journal

Supporting Information

No Transition Metals Required – Oxygen Promoted Synthesis of Imines from Primary Alcohols and Amines under Ambient Conditions

Daniel Himmelbauer, Radu Talmazan, Stefan Weber, Jan Pecak, Antonio Thun-Hohenstein, Maxine-Sophie Geissler, Lukas Pachmann, Marc Pignitter, Maren Podewitz,* and Karl Kirchner*

1. General Information 2
2. Experimental protocols 3
3. Spectroscopic Data for Imine Products 4
4. Experimental mechanistic studies 12
5. Computational studies 17
6. NMR Spectra of Imines Products 20
7. References 55

1. General Information

All reactions were performed under ambient conditions and solvents were used as purchased, if not stated otherwise. All aniline and benzyl alcohol substrates as well as all bases were purchased from commercially available sources like Sigma-Aldrich, Acros Organics or TCI and used without further purification. The deuterated solvents were purchased from Eurisotope and dried over 3 Å molecular sieves. ^{1}H and $^{13}C\{^{1}H\}$ spectra were recorded on Bruker AVANCE-250, AVANCE-400, and AVANCE-600 spectrometers. ^{1}H and $^{13}C\{^{1}H\}$ NMR spectra were referenced internally to residual protio-solvent, and solvent resonances, respectively, and are reported relative to tetramethylsilane (δ = 0 ppm).

High resolution-accurate mass data mass spectra were recorded on a hybrid Maxis Qq-aoTOF mass spectrometer (Bruker Daltonics, Bremen, Germany) fitted with an ESI-source. Measured accurate mass data of the [M]+ ions for confirming calculated elemental compositions were typically within ±5 ppm accuracy. The mass calibration was done with a commercial mixture of perfluorinated trialkyl-triazines (ES Tuning Mix, Agilent Technologies, Santa Clara, CA, USA).

GC–MS analysis was conducted on an ISQ LT Single quadrupole MS (Thermo Fisher) directly interfaced to a TRACE 1300 Gas Chromatographic systems (Thermo Fisher), using a Rxi-5Sil MS (30 m, 0.25mm ID) cross-bonded dimethyl polysiloxane capillary column.

CW-EPR spectroscopic measurements were performed on an Xband Bruker Elexsys-II E500 EPR spectrometer (Bruker Biospin GmbH, Rheinstetten, Germany) in solution at 100 K. A highsensitivity cavity (SHQE1119) was used for measurements, setting the microwave frequency to 9.86 GHz, the modulation frequency to 100 kHz, the center field to 6000 G, the sweep width to 12000 G, the sweep time to 120 s, the modulation amplitude to 4 G, the microwave power to 15.9 mW, the conversion time to 7.33 ms, and the resolution to 4096 points. The spectra were analyzed using the Bruker Xepr software.

2. Experimental protocols

General protocol for N-Alkylation

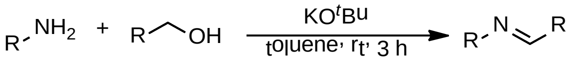

KO^{t}Bu (146 mg, 1.3 mmol) was put into a 10 ml flat-bottom vial. Then a toluene (6 ml) solution of the aniline derivative (110 µl, 1.2 mmol) and alcohol (104 µl, 1.0 mmol) was added. The reaction mixture was stirred for 3 h at room temperature, while exposed to ambient atmospheric conditions. A sample was taken for GC-MS analysis with n-dodecane used as internal standard. Afterwards, the reaction mixture was filtered over a pad of Celite, and the solvent was removed under reduced pressure to yield the crude product which was purified and isolated as described below.

Further optimization reactions

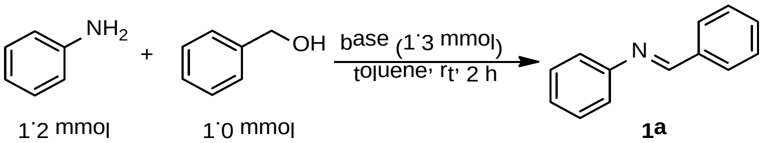

1.2 mmol 1.0 mmol **1a**

Table S1: Further optimization reactions of aniline with benzyl alcohol.[a]

Entry	Base	Conversion [%]	Additive
1	KO^{t}Bu	>99	TEMPO
2[b]	KO^{t}Bu	82	TEMPO
3[b]	-	2	TEMPO
4	KO^{t}Bu	0	3,4-chromanediol
5[b,c]	KO^{t}Bu	19	-

[a] Reaction conditions: Aniline (55 µl, 0.6 mmol), benzyl alcohol (52 µl, 0.5 mmol), KO^{t}Bu (73 mg, 0.65 mmol), solvent (6 ml), additive (0.65 mmol), ambient conditions, room temperature. Conversion of **1a** was determined by GC/MS. [b] Exclusion of oxygen. [c] Reaction solution was radiated with UV light.

ICP-MS analysis

ICP-MS measurements were carried out on an iCAP Qc quadrupole inductively coupled plasma-mass spectrometer (ICP-MS, Thermo, Bremen), using typical instrumental settings for kinetic energy discrimination mode. Potassium *tert*-butoxide used in all performed and described experiments was carefully analyzed. Samples were diluted with 1% nitric acid solution, prepared from plasma-pure nitric acid and double distilled water. Additionally, a selection of organic substrates was analyzed by LA-ICP-MS. Only ppb traces of transition metals such as Pd or Pt were found.

3. Spectroscopic Data for Imine Products

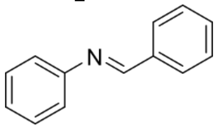

(E)-N-benzylideneaniline (**1a**)[1]. The crude product was purified by recrystallisation in *n*-heptane (1 ml) yielding 157 mg (87 % isolated yield) as white solid. **^{1}H NMR** (δ, 400 MHz, CD_2Cl_2, 25 °C): 8.48 (s, 1H), 7.92 - 7.90 (m, 2H), 7.49 (dd, *J* = 5.1 Hz, *J* = 1.9 Hz 3H), 7.42 - 7.38 (m, 2H), 7.26 - 7.20 (m, 3H). **^{13}C{^{1}H} NMR** (δ, 101 MHz, CD_2Cl_2, 25 °C): 160.6, 152.5, 136.8, 131.7, 129.5, 129.1, 129.1, 126.3, 121.2.

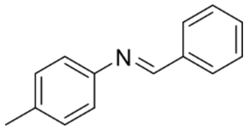

(E)-N-benzylidene-4-methylaniline (**1b**)[2]. The crude product was purified by Al_2O_3 120 (12 g, basic activated) column chromatography (*n*-heptane/Et_2O 20:1) yielding 183 mg (94 % isolated yield) as yellow liquid. **^{1}H NMR** (δ, 400 MHz, CD_2Cl_2, 25 °C): 8.48 (s, 1H), 7.92 - 7.88 (m, 2H), 7.50 - 7.47 (m, 3H), 7.23 - 7.20 (m, 2H), 7.15 - 7.12 (m, 2H), 2.37 (s, 3H, CH_3). **^{13}C{^{1}H} NMR** (δ, 101 MHz, CD_2Cl_2, 25 °C): 159.7, 149.8, 136.9, 136.3, 131.5, 130.1, 129.1, 129.0, 121.1, 21.1.

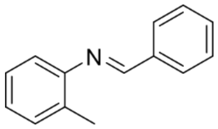

(E)-1-phenyl-N-(o-tolyl)methanimine (**1c**)[3]. The crude product was purified by Al_2O_3 120 (12 g, basic activated) column chromatography (*n*-heptane/Et_2O 20:1) yielding 173 mg (89 % isolated yield) as yellow liquid. **^{1}H NMR** (δ, 400 MHz, CD_2Cl_2, 25 °C): 8.38 (s, 1H), 7.95 - 7.91 (m, 2H), 7.51 - 7.48 (m, 3H), 7.24 - 7.20 (m, 2H), 7.13 (td, *J* = 7.4 Hz, *J* = 1.3 Hz, 1H), 6.94 (dd, *J* = 7.6 Hz, *J* = 1.1 Hz, 1H), 2.35 (s, 3H, CH_3). **^{13}C{^{1}H} NMR** (δ, 101 MHz, CD_2Cl_2, 25 °C): 159.8, 151.6, 137.0, 132.3, 131.6, 130.6, 129.1, 129.1, 127.1, 126.0, 117.9, 17.9.

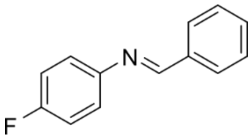

(E)-N-benzylidene-4-fluoroaniline (**1d**)[2]. The crude product was purified by Al_2O_3 120 (12 g, basic activated) column chromatography (*n*-heptane/Et_2O 2:1) yielding 167 mg (84 % isolated yield) as pale-yellow solid. **^{1}H NMR** (δ, 400 MHz, CD_2Cl_2, 25 °C): 8.46 (s, 1H), 7.91 - 7.89 (m, 2H), 7.50 - 7.49 (m, 3H), 7.23 - 7.20 (m, 2H), 7.12 - 7.08 (m, 2H). **^{13}C{^{1}H} NMR** (δ, 101 MHz, CD_2Cl_2, 25 °C): 162.8, 160.5, 160.4, 148.6, 136.7, 131.8, 129.1 (d, *J* = 7.1 Hz), 122.7 (d, *J* = 8.3 Hz), 116.2 (d, *J* = 22.6 Hz).

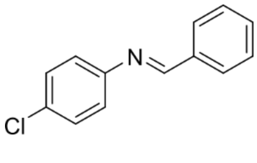

(E)-N-benzylidene-4-chloroaniline (**1e**)[4]. The crude product was purified by recrystallisation in *n*-heptane (1 ml) yielding 181 mg (84 % isolated yield) as white solid. **^{1}H NMR** (δ, 400 MHz, CD_2Cl_2, 25 °C): 8.45 (s, 1H), 7.92 - 7.89 (m, 2H), 7.52 - 7.47 (m, 3H), 7.39 - 7.36 (m, 2H), 7.19 - 7.15 (m, 2H). **^{13}C{^{1}H} NMR** (δ, 101 MHz, CD_2Cl_2, 25 °C): 161.1, 151.0, 136.5, 132.0, 131.7, 129.6, 129.2, 129.2, 122.7.

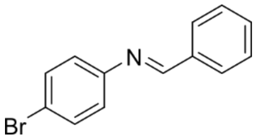

(E)-N-benzylidene-4-bromoaniline (**1f**)[4]. The crude product was purified by recrystallisation in *n*-heptane (1 ml) yielding 216 mg (83 % isolated yield) as white solid. **^{1}H NMR** (δ, 400 MHz, CD_2Cl_2, 25 °C): 8.45 (s, 1H), 7.92 - 7.89 (m, 2H), 7.54 - 7.48 (m, 5H), 7.13 - 7.09 (m, 2H). **^{13}C{^{1}H} NMR** (δ, 101 MHz, CD_2Cl_2, 25 °C): 161.1, 151.5, 136.5, 132.6, 132.0, 129.2, 129.2, 123.0, 119.5.

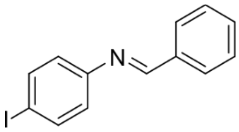

(E)-N-benzylidene-4-iodoaniline (**1g**)[4]. The crude product was purified by recrystallisation in *n*-heptane (1 ml) yielding 268mg (87 % isolated yield) as pale-yellow solid. **^{1}H NMR** (δ, 400 MHz, CD_2Cl_2, 25 °C): 8.44 (s, 1H), 7.91 - 7.89 (m, 2H), 7.74 - 7.70 (m, 2H), 7.52 - 7.47 (m, 3H). 7.00 - 6.96 (m, 2H). **^{13}C{^{1}H} NMR** (δ, 101 MHz, CD_2Cl_2, 25 °C): 161.2, 152.2, 138.6, 136.5, 132.0, 129.2, 129.2, 123.4, 90.4.

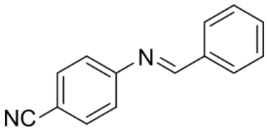

(E)-4-(benzylideneamino)benzonitrile (**1h**)[5].The crude product was purified by recrystallisation in *n*-heptane/Et_2O (1 ml, 1:1) yielding 185 mg (77 % isolated yield) as pale-yellow solid. **^{1}H NMR** (δ, 400 MHz, CD_2Cl_2, 25 °C): 8.43 (s, 1H), 7.92 (m, 2H), 7.70 (m, 2H), 7.55 - 7.49 (m, 3H), 7.24 (m, 2H).**^{13}C{^{1}H} NMR** (δ, 101 MHz, CD_2Cl_2, 25 °C): 162.8, 156.5, 136.1, 133.8, 132.5, 129.5, 129.3, 121.9, 119.3, 109.4.

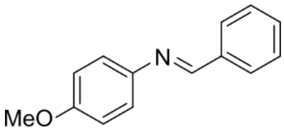

(E)-*N*-benzylidene-4-methoxyaniline (**1i**)[6]. The crude product was purified by Al_2O_3 60 (12 g, basic activated) column chromatography (*n*-heptane/Et_2O 2:1) yielding 194 mg (92 % isolated yield) as pale-yellow solid. **^{1}H NMR** (δ, 400 MHz, CD_2Cl_2, 25 °C): 8.50 (s, 1H), 7.89 (m, 2H), 7.48 (m, 3H), 7.24 (m, 2H), 6.94 (m, 2H), 3.82 (s, 3H).**^{13}C{^{1}H} NMR** (δ, 101 MHz, CD_2Cl_2, 25 °C): 158.8, 158.5, 145.2, 137.0, 131.3, 129.1, 128.9, 122.5, 114.7, 55.9.

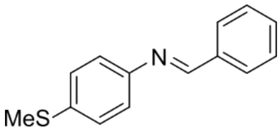

(E)-N-benzylidene-4-(methylthio)aniline (**1j**)[7]. The crude product was purified by recrystallisation in *n*-heptane/toluene (2 ml, 5:1) yielding 214 mg (94 % isolated yield) as brown solid. **^{1}H NMR** (δ, 400 MHz, CD_2Cl_2, 25 °C): 8.49 (s, 1H), 7.90 (m, 2H), 7.49 (m, 3H), 7.30 (m, 2H), 7.19 (m, 2H), 2.51 (s, 3H).**^{13}C{^{1}H} NMR** (δ, 101 MHz, CD_2Cl_2, 25 °C): 159.9, 149.6, 136.8, 136.5, 131.7, 129.2, 129.1, 127.8, 121.9, 16.4.

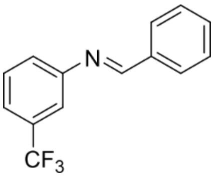

(E)-N-benzylidene-4-(trifluoromethyl)aniline (**1k**)[8]. The crude product was purified by Al_2O_3 150 (12 g, basic activated) column chromatography (*n*-heptane/Et_2O 1:2) yielding 228 mg (91 % isolated yield) as pale-yellow solid. **^{1}H NMR** (δ, 400 MHz, CD_2Cl_2, 25 °C): 8.49 (s, 1H), 7.93 (m, 2H), 7.56 – 7.48 (m, 5H), 7.46 (m, 1H), 7.40 (m, 1H). **^{13}C{^{1}H} NMR** (δ, 101 MHz, CD_2Cl_2, 25 °C): 162.2, 153.1, 136.3, 132.2, 131.7 (d, *J* = 32.1 Hz), 130.2, 129.4, 129.2, 124.7 124.6 (d, *J* = 272 Hz), 122.7 (q, *J* = 3.8 Hz), 118.2 (q, *J* = 3.8 Hz).

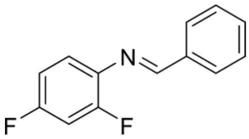

(E)-N-benzylidene-2,4-difluoroaniline (**1l**)[9].The crude product was purified by Al_2O_3 150 (12 g, basic activated) column chromatography (*n*-heptane) yielding 192 mg (88 % isolated yield) as yellow liquid. **^{1}H NMR** (δ, 400 MHz, CD_2Cl_2, 25 °C): 8.52 (s, 1H), 7.93 - 7.91 (m, 2H), 7.55 - 7.47 (m, 3H), 7.20 - 7.18 (m, 1H), 6.97 - 6.90 (m, 2H).**^{13}C{^{1}H} NMR** (δ, 101 MHz, CD_2Cl_2, 25 °C): 163.0 (t, *J* = 2.2 Hz), 162.2 (d, *J* = 11.2 Hz), 159.8 (d, *J* = 11.3 Hz), 157.0 (d, *J* = 12.0 Hz), 154.5 (d, *J* = 12.3 Hz), 136.8 (dd, *J* = 10.4 Hz, *J* = 3.7 Hz), 136.4, 132.2, 129.2 (d, *J* = 4.9 Hz), 122.5 (dd, *J* = 9.6 Hz, *J* = 3.2 Hz), 111.7 (dd, *J* = 22.1 Hz, *J* = 4.0 Hz), 104.9 (dd, *J* = 26.3 Hz, *J* = 24.3 Hz).

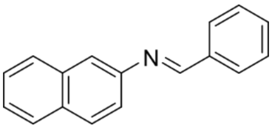

(E)-N-benzylidene-2-napthylamine (**1m**)[10]. The crude product was purified by recrystallisation in *n*-heptane (1 ml) yielding 268mg (87 % isolated yield) as pale-yellow solid. **^{1}H NMR** (δ, 400 MHz, CD_2Cl_2, 25 °C): 8.62 (s, 1H), 7.99 - 7.97 (m, 2H), 7.91 - 7.87 (m, 3H), 7.63 (m, 1H), 7.54 - 7.45 (m, 6H). **^{13}C{^{1}H} NMR** (δ, 101 MHz, CD_2Cl_2, 25 °C): 160.8, 150.0, 136.8, 134.6, 132.4, 131.8, 129.3, 129.2, 129.2, 128.3, 128.1, 126.8, 125.8, 121.5, 118.1.

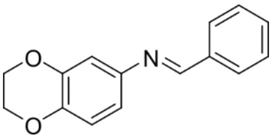

(E)-N-benzylidene-2,3-dihydrobenzo[b][1,4]dioxin-6-amine (**1n**)[11]. The crude product was purified by Al_2O_3 60 (6 g, basic activated) column chromatography (*n*-heptane/toluene/Et_2O 2:1:0.1) yielding 207 mg (87 % isolated yield) as pale-yellow solid. **^{1}H NMR** (δ, 400 MHz, CD_2Cl_2, 25 °C): 8.46 (s, 1H), 7.87 (m, 2H), 7.47 (m, 3H), 6.87 (dd, *J* = 8.3 Hz, *J* = 0.5 Hz, 1H), 6.81 – 6.77 (m, 2H), 4.27 (s 4H). **^{13}C{^{1}H} NMR** (δ, 101 MHz, CD_2Cl_2, 25 °C): 158.9, 146.0, 144.3, 142.8, 136.9, 131.4, 129.1, 128.9, 117.8 114.9, 110.0, 65.0.

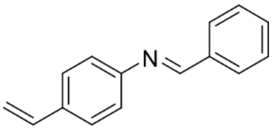

(E)-*N*-benzylidene-4-vinylaniline (**1o**)[12]. The crude product was purified by recrystallisation in *n*-heptane (4 ml) yielding 193 mg (93 % isolated yield) as yellow liquid. **^{1}H NMR** (δ, 400 MHz, CD_2Cl_2, 25 °C): 8.50 (s, 1H), 7.91 (m, 2H), 7.48 (m, 5H), 7.20 (m, 2H), 6.75 (dd, *J* = 17.6 Hz, *J* = 10.9 Hz, 1H), 5.76 (dd, *J* = 17.6 Hz, *J* = 0.9 Hz, 1H), 5.24 (dd, *J* = 10.9 Hz, *J* = 0.9 Hz, 1H). **^{13}C{^{1}H} NMR** (δ, 101 MHz, CD_2Cl_2, 25 °C): 160.3, 151.9, 136.8, 136.7, 135.9, 131.8, 129.2, 129.1, 127.4 121.5, 113.5.

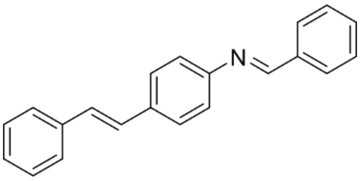

(E)-N-benzylidene-4-aminostilbene (**1p**)[4]. The crude product was purified by recrystallisation in *n*-heptane/toluene (4 ml, 1:1) yielding 232 mg (82 % isolated yield) as gold/brown solid. **^{1}H NMR** (δ, 400 MHz, CD_2Cl_2, 25 °C): 8.53 (s, 1H), 7.93 (m, 2H), 7.56 (m, 4H), 7.50 (m, 3H), 7.38 (m, 2H), 6.26 (s, 3H), 7.16 (d, *J* = 2.5 H, 2H). **^{13}C{^{1}H} NMR** (δ, 101 MHz, CD_2Cl_2, 25 °C): 160.2, 151.7, 137.8, 136.8, 135.7, 131.8, 129.2, 129.1, 129.1, 128.5, 128.4, 128.0, 127.7, 126.8, 121.8, 53.8.

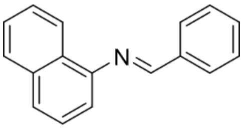

(E)-N-benzylidene-1-napthylamine (**1q**)[2]. The crude product was purified by Al_2O_3 60 (7 g, basic activated) column chromatography (*n*-heptane) yielding 175 mg (76 % isolated yield) as orange oil. **^{1}H NMR** (δ, 400 MHz, CD_2Cl_2, 25 °C): 8.58 (s, 1H), 8.36 - 8.33 (m, 1H), 8.05 - 8.03 (m, 2H), 7.89 - 7.86 (m, 1H), 7.75 - 7.73 (m, 1H), 7.55 - 7.47 (m, 6H), 7.10 - 7.08 (m, 1H).**^{13}C{^{1}H} NMR** (δ, 101 MHz, CD_2Cl_2, 25 °C): 160.8, 149.7, 136.9, 134.4, 131.9, 129.3, 129.3, 129.2 128.0, 126.8, 126.5, 126.1, 126.1, 124.3, 113.0.

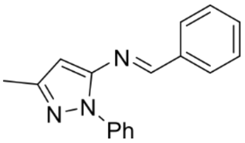

(E)-N-benzylidene-3-methyl-1-phenyl-1H-pyrazol-5-amine (**1r**). The crude product was purified by Al_2O_3 60 (6 g, basic activated) column chromatography (*n*-heptane/toluene/Et_2O 2:1:0.1) yielding 237 mg (91 % isolated yield) as pale-yellow solid. **^{1}H NMR** (δ, 400 MHz, CD_2Cl_2, 25 °C): 8.66 (s, 1H), 7.87 (m, 2H), 7.75 (m, 2H), 7.47 (m, 5H), 7.32 (m, 1H), 6.22 (s, 1H), 2.33 (s, 3H). **^{13}C{^{1}H} NMR** (δ, 101 MHz, CD_2Cl_2, 25 °C): 160.7, 150.8, 149.5, 136.4, 132.3, 129.4, 129.3, 128.9, 126.6, 124.2, 93.6, 14.3. HR-MS (ESI^+, CH_3CN/MeOH + 1 % H_2O): m/z calcd for $C_{17}H_{16}N_3$ $[M+H]^+$ 262.1339, found 262.1331.

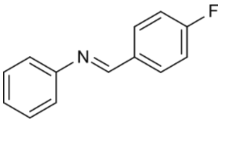

(*E)-N-(4-fluorobenzylidene)aniline* (**1s**)[2]. The crude product was purified by Al_2O_3 150 (12 g, basic activated) column chromatography (*n*-heptane/Et_2O 20:1) yielding 185 mg (88 % isolated yield) as white solid. **^{1}H NMR** (δ, 400 MHz, CD_2Cl_2, 25 °C): 8.44 (s, 1H), 7.92 (m, 2H), 7.40 (m, 2H), 7.26 – 7.16 (m, 5H). **^{13}C{^{1}H} NMR** (δ, 101 MHz, CD_2Cl_2, 25 °C): 165.1 (d, *J* = 251 Hz), 159.1, 152.3, 133.2 (d, *J* = 3.2 Hz), 131.1 (d, *J* = 8.8 Hz), 129.6, 126.4, 121.2, 116.2 (d. *J* = 21.9).

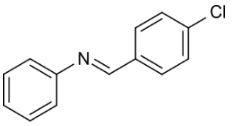

(E)-N-(4-chlorobenzylidene)aniline (**1t**)[4]. The crude product was purified by recrystallisation in *n*-heptane (2 ml) yielding 204 mg (95 % isolated yield) as pale-yellow solid. **^{1}H NMR** (δ, 400 MHz, CD_2Cl_2, 25 °C): 8.45 (s, 1H), 7.87 (m, 2H), 7.47 (m, 2H), 7.40 (m, 2H), 7.27 – 7.20 (m, 3H). **^{13}C{^{1}H} NMR** (δ, 101 MHz, CD_2Cl_2, 25 °C): 159.1, 152.1, 137.5, 135.4, 130.3, 129.6, 129.4, 126.5, 121.2.

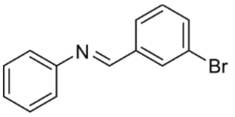

(E)-N-(3-bromobenzylidene)aniline (**1u**)[13]. The crude product was purified by Al_2O_3 150 (12 g, basic activated) column chromatography (*n*-heptane/Et_2O 20:1) yielding 232 mg (89 % isolated yield) as yellow liquid. **^{1}H NMR** (δ, 400 MHz, CD_2Cl_2, 25 °C): 8.42 (s, 1H), 8.11 (m, 1H), 7.82 (dt, *J* = 7.7 Hz, *J* = 1.2 Hz, 1H), 7.63 (ddd, *J* = 8.0 Hz, *J* = 2.1 Hz, *J* = 1.1 Hz, 1H), 7.43 – 7.36 (m, 3H), 7.28 – 7.21 (m, 3H). **^{13}C{^{1}H} NMR** (δ, 101 MHz, CD_2Cl_2, 25 °C): 158.8, 151.9, 138.8, 134.5, 131.5, 130.8, 129.6, 128.0, 126.7, 123.3, 121.2.

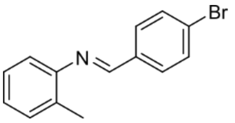

(E)-1-(4-bromophenyl)-N-(o-tolyl)methanimine (**1v**)[14]. The crude product was purified by Al_2O_3 150 (12 g, basic activated) column chromatography (*n*-heptane/Et_2O 20:1) yielding 95 mg (46 % isolated yield) as yellow oil. **^{1}H NMR** (δ, 400 MHz, CD_2Cl_2, 25 °C): 8.34 (s, 1H), 7.81 (m, 2H), 7.63 (m, 2H), 7.21 (m, 2H), 7.13 (td, *J* = 7.4 Hz, *J* = 1.4 Hz, 1H), 6.94 (dd, *J* = 7.7 Hz, *J* = 1.4 Hz, 1H), 2.34 (s, 3H). **^{13}C{^{1}H} NMR** (δ, 101 MHz, CD_2Cl_2, 25 °C): 158.4, 151.1, 136.0, 132.5, 132.3, 130.6, 130.5, 127.1, 126.3, 125.9, 117.7, 17.9.

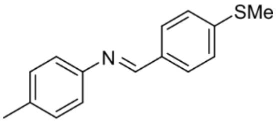

(E)-1-(4-(methylthio)phenyl)-N-(p-tolyl)methanimine (**1w**)[15]. The crude product was purified by recrystallisation in *n*-heptane (1 ml) yielding 96 mg (41 % isolated yield) as pale-yellow solid. **^{1}H NMR** (δ, 400 MHz, CD_2Cl_2, 25 °C): 8.42 (s, 1H), 7.80 (m, 2H), 7.31 (m, 2H), 7.31 (m, 2H), 7.20 (m, 2H), 7.12 (m, 2H), 2.54 (s, 3H), 2.36 (s, 3H). **^{13}C{^{1}H} NMR** (δ, 101 MHz, CD_2Cl_2, 25 °C): 159.0, 149.8, 143.5, 136.2, 133.5, 130.1, 129.3, 126.0, 121.1, 21.1, 15.3.

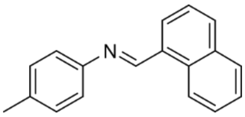

(E)-1-(naphthalen-1-yl)-N-(p-tolyl)methanimine (**1x**)[16]. The crude product was purified by Al_2O_3 150 (2 g, basic activated) column chromatography (*n*-heptane) yielding 208 mg (85 % isolated yield) as yellow oil. **^{1}H NMR** (δ, 400 MHz, CD_2Cl_2, 25 °C): 9.19 (d, *J* = 8.7 Hz, 1H), 9.14 (s, 1H), 8.13 (dd, *J* = 7.2 Hz, *J* = 1.1 Hz, 1H), 8.01 (d, *J* = 8.2 Hz, 1H), 7.97 (m, 1H), 7.68 (ddd, *J* = 8.5 Hz, *J* = 6.2 Hz, *J* = 1.5 Hz, 1H), 7.62 (m, 2H), 7.29 (m, 4H), 2.45 (s, 3H). **^{13}C{^{1}H} NMR** (δ, 101 MHz, CD_2Cl_2, 25 °C): 159.6, 150.4, 136.3, 134.4, 132.1, 132.0, 131.8, 130.3, 130.2, 129.1, 127.7, 126.6, 125.7, 124.8, 121.2, 21.2.

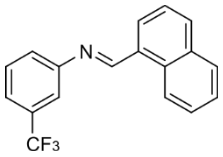

(E)-1-(naphthalen-1-yl)-*N*-(3-(trifluoromethyl)phenyl)methanimine (**1y**). The crude product was purified by Al_2O_3 150 (2 g, basic activated) column chromatography (*n*-heptane) yielding 236 mg (79 % isolated yield) as yellow oil. **^{1}H NMR** (δ, 400 MHz, CD_2Cl_2, 25 °C): 9.16 (d, *J* = 8.8 Hz, 1H), 9.09 (s, 1H), 8.11 (dd, *J* = 7.2 Hz, *J* = 1.1 Hz, 1H), 8.04 (d, *J* = 8.2 Hz, 1H), 7.97 (m, 1H), 7.69 - 7.47 (m, 6H), 7.49 (m, 1H). **^{13}C{^{1}H} NMR** (δ, 101 MHz, CD_2Cl_2, 25 °C): 162.2, 153.6, 134.4, 132.9, 131.9, 131.8, 131.6, 131.4, 131.3, 130.3, 129.2, 128.1, 126.8, 126.0, 125.7, 124.8, 124.7, 123.3, 122.7, 122.7, 122.7, 122.6, 118.3, 118.3, 118.2, 118.2. HR-MS (ESI$^+$, CH_3CN/MeOH + 1 % H_2O): m/z calcd for $C_{18}H_{12}F_3N$ $[M+H]^+$ 300.0995, found 300.0989.

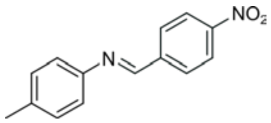

(E)-4-methyl-N-(4-nitrobenzylidene)aniline (**1z**)[16]. The crude product was purified by Al_2O_3 150 (4 g, basic activated) column chromatography (*n*-heptane/Et_2O 1:1) yielding 125 mg (64 % isolated yield) as yellow solid. **^{1}H NMR** (δ, 400 MHz, CD_2Cl_2, 25 °C): 8.59 (s, 1H), 8.31 (m, 2H), 8.08 (m, 2H), 7.22 (m, 4H), 2.38 (s, 3H). **^{13}C{^{1}H} NMR** (δ, 101 MHz, CD_2Cl_2, 25 °C): 156.9, 149.6, 148.7, 142.3, 137.7, 130.3, 129.6, 124.3, 121.4, 21.2.

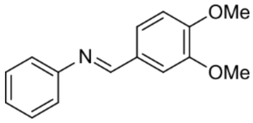

(E)-N-(3,4-Dimethoxybenzylidene)aniline (**1aa**)[17]. The crude product was purified by Al_2O_3 150 (2 g, basic activated) column chromatography (*n*-heptane/Et_2O 1:1) yielding 195 mg (81 % isolated yield) as yellow oil. **^{1}H NMR** (δ, 400 MHz, CD_2Cl_2, 25 °C): 8.37 (s, 1H), 7.60 (d, *J* = 7.6 Hz, 1H), 7.39 (m, 2H), 7.34 (dd, *J* = 8.2 Hz, *J* = 1.9 Hz, 1H), 7.24 - 7.18 (m, 3H), 6.95 (d, *J* = 8.2 Hz, 1H), 3.93 (s, 3H), 3.90 (s, 3H). **^{13}C{^{1}H} NMR** (δ, 101 MHz, CD_2Cl_2, 25 °C): 160.1, 152.7, 152.7, 150.0, 130.0, 129.5, 125.9, 124.5, 121.2, 111.1, 109.6, 56.3, 56.2.

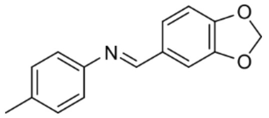

(E)-1-(benzo[d][1,3]dioxol-5-yl)-N-(p-tolyl)methanimine (**1ab**)[10]. The crude product was purified by recrystallisation in *n*-heptane (1 ml) yielding 208 mg (87 % isolated yield) as pale-yellow solid. **^{1}H NMR** (δ, 400 MHz, CD_2Cl_2, 25 °C): 8.36 (s, 1H), 7.51 (d, *J* = 7.5 Hz, 1H), 7.27 (dd, *J* = 8.0 Hz, *J* = 1.6 Hz, 1H), 7.19 (m, 2H), 7.10 (m, 2H), 6.89 (d, *J* = 8.0 Hz, 1H), 6.04 (s, 2H), 2.36 (s, 3H). **^{13}C{^{1}H} NMR** (δ, 101 MHz, CD_2Cl_2, 25 °C): 158.8, 150.8, 149.8, 148.9, 136.0, 131.9, 130.1, 125.9, 121.1, 108.5, 106.9, 102.2, 21.1.

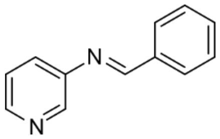

(E)-*N*-benzylidene-3-pyridinamine (**1ac**)[10]. The crude product was purified by Al_2O_3 150 (6 g, basic activated) column chromatography (*n*-heptane/toluene 1:1) yielding 116 mg (87 % isolated yield) as yellow liquid. **^{1}H NMR** (δ, 400 MHz, CD_2Cl_2, 25 °C): 8.49 (s, 1H), 8.48 – 8.45 (m, 2H), 7.93 (m, 2H), 7.54 – 7.50 (m, 4H), 7.33 (ddd, *J* = 8.1 Hz, *J* = 4.7 Hz, *J* = 0.8 Hz, 2H). **^{13}C{^{1}H} NMR** (δ, 101 MHz, CD_2Cl_2, 25 °C): 162.3, 148.1, 147.5, 143.2, 136.3, 132.2, 129.3, 129.2, 127.8 124.0.

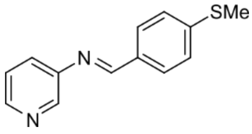

(E)-1-(4-(methylthio)phenyl)-N-(pyridin-3-yl)methanimine (**1ad**)[18]. The crude product was purified by Al_2O_3 150 (10 g, basic activated) column chromatography (Et_2O) yielding 196 mg (86 % isolated yield) as white solid. **^{1}H NMR** (δ, 400 MHz, CD_2Cl_2, 25 °C): 8.44 (m, 3H), 7.83 (m, 2H), 7.51 (ddd, *J* = 8.1 Hz, *J* = 2.6 Hz, *J* = 1.6 Hz, 1H), 7.51 (m, 3H), 2.54 (s, 3H). **^{13}C{^{1}H} NMR** (δ, 101 MHz, CD_2Cl_2, 25 °C): 161.6, 148.2, 147.4, 144.6, 143.2, 132.9, 129.6, 127.8, 125.9, 124.0, 15.2.

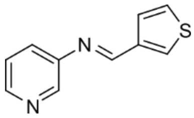

(E)-N-(pyridin-3-yl)-1-(thiophen-3-yl)methanimine (**1ae**). The crude product was purified by Al_2O_3 150 (2 g, basic activated) column chromatography (Et_2O) yielding 158 mg (84 % isolated yield) as yellow oil. **^{1}H NMR** (δ, 400 MHz, CD_2Cl_2, 25 °C): 8.49 (s, 1H), 8.44 (m, 2H), 7.88 (dd, *J* = 2.9 Hz, *J* = 1.1 Hz, 1H), 7.68 (ddd, *J* = 5.1 Hz, *J* = 1.2 Hz, *J* = 0.4 Hz, 1H), 7.50 (ddd, *J* = 8.1 Hz, *J* = 2.6 Hz, *J* = 1.6 Hz, 1H), 7.43 (ddd, *J* = 5.1 Hz, *J* = 2.9 Hz, *J* = 0.7 Hz, 1H), 7.31 (ddd, *J* = 8.1 Hz, *J* = 4.8 Hz, *J* = 0.7 Hz, 1H). **^{13}C{^{1}H} NMR** (δ, 101 MHz, CD_2Cl_2, 25 °C): 156.3, 148.2, 147.4, 143.1, 141.0, 131.6, 127.8, 127.4, 126.1, 124.0. HR-MS (ESI^+, CH_3CN/MeOH + 1 % H_2O): m/z calcd for $C_{10}H_8N_2S$ $[M+H]^+$ 189.0481, found 189.0485.

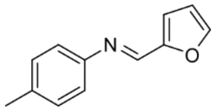

(E)-1-(furan-2-yl)-N-(p-tolyl)methanimine (**1af**)[19]. The crude product was purified by Al_2O_3 150 (12 g, basic activated) column chromatography (*n*-heptane/Et_2O 20:1) yielding 172 mg (93 % isolated yield) as yellow oil. **^{1}H NMR** (δ, 400 MHz, CD_2Cl_2, 25 °C): 8.30 (s, 1H), 7.62 (d, *J* = 1.7 Hz, 1H), 7.20 (m, 2H), 7.13 (m, 2H), 6.95 (dd, *J* = 3.5 Hz, *J* = 0.6 Hz, 1H), 6.57 (dd, *J* = 3.5 Hz, *J* = 1.8 Hz, 1H), 2.36 (s, 3H). **^{13}C{^{1}H} NMR** (δ, 101 MHz, CD_2Cl_2, 25 °C): 153.0, 149.3, 147.3, 145.8, 136.6, 130.2, 121.2, 115.9, 112.5, 21.1.

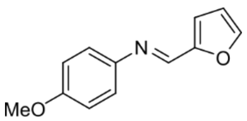

(E)-1-(furan-2-yl)-N-(4-methoxyphenyl)methanimine (**1ag**)[20].The crude product was purified by Al_2O_3 150 (12 g, basic activated) column chromatography (*n*-heptane/toluene/Et_2O 1:1:0.1) yielding 105 mg (52 % isolated yield) as yellow oil. **^{1}H NMR** (δ, 400 MHz, CD_2Cl_2, 25 °C): 8.31 (s, 1H), 7.61 (d, *J* = 1.7 Hz, 1H), 7.22 (m, 2H), 6.92 (m, 3H), 6.57 (dd, *J* = 3.5 Hz, *J* = 1.8 Hz, 1H), 3.81 (s, 3H). **^{13}C{^{1}H} NMR** (δ, 101 MHz, CD_2Cl_2, 25 °C): 158.9, 153.1, 146.1, 145.7, 144.7, 122.6, 115.5, 114.8, 112.5, 55.8.

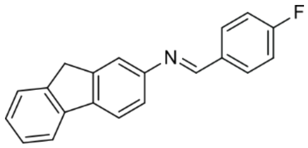

(E)-N-(4-fluorobenzylidene)-9H-fluoren-2-amine (**1ah**). The crude product was purified by Al_2O_3 150 (4 g, basic activated) column chromatography (*n*-heptane/acetone 1:1) yielding 155 mg (54 % isolated yield) as pale-yellow solid. **^{1}H NMR** (δ, 400 MHz, CD_2Cl_2, 25 °C): 8.54 (s, 1H), 7.95 (m, 2H), 7.80 (t, *J* = 8.1 Hz, 2H), 7.56 (m, 1H), 7.43 (m, 1H), 7.39 (m, 1H), 7.31 (dd, *J* = 7.4 Hz, *J* = 1.2 Hz, 1H), 7.27 (m, 1H), 7.20 (m, 2H), 3.95 (s, 2H). **^{13}C{^{1}H} NMR** (δ, 101 MHz, CD_2Cl_2, 25 °C): 166.3, 163.8, 158.3, 151.1, 145.0, 143.9, 141.7, 140.3, 133.4, 131.1, 131.1, 127.2, 126.9, 125.4, 120.7, 120.5, 120.1, 118.0, 116.3, 116.1, 37.3. HR-MS (ESI^+, CH_3CN/MeOH + 1 % H_2O): m/z calcd for $C_{20}H_{15}FN$ $[M+H]^+$ 288.1183, found 288.1187.

4. Experimental mechanistic studies

N-Alkylation of aniline under pure O_2 atmosphere

KO^{t}Bu (0.65 mmol) and molecular sieves (15 pellets, 4 Å) were put into a 10 mL screwcap-vial containing a magnetic stir bar. Then a toluene (3 mL) solution containing aniline (0.60 mmol) and benzyl alcohol (0.50 mmol) was added. The vial was closed and flushed with pure oxygen for 1 min and then stirred for 15 min. A GC-MS analysis showed full conversion.

N-Alkylation of aniline in the presence of 18-crown-6 ether

KO^{t}Bu (0.65 mmol) and molecular sieves (15 pellets, 4 Å) were put into a 10 mL flat-bottom vial containing a magnetic stir bar. Then a toluene (3 mL) solution containing aniline (0.60 mmol), benzyl alcohol (0.50 mmol) and 18-crown-6 ether (0.65 mmol) was added. The reaction mixture was stirred for 3 h at room temperature, while exposed to ambient atmospheric conditions. A GC-MS analysis showed 88% conversion.

Synthesis of benzyl alcohol [^{18}O]

Benzyl alcohol [^{18}O] was synthesized by slightly modified procedure from the literature.[21]

Toluene-4-sulforic acid (2mg) was added to methyl orthobenzoate (925.3 µl, 5.38 mmol, 1 equiv.) in a Schlenk tube under argon atmosphere. [^{18}O]Water (99% ^{18}O) (100 µl, 5.54 mmol, 1.03 equiv.) was added under rapid stirring. The mixture became homogenous after 2 minutes of stirring. The solution was stirred for further 30 minutes. Methanol was gently removed in vacuum (100 mbar, 30 minutes). 10 mL dry diethyl ether was added, and the mixture was cooled to 0 °C. Solid lithium aluminum hydride (217.8 mg, 5.65 mmol, 1.05 equiv.) was added in batch. The suspension was stirred for 5 minutes at 0 °C and 16 hours at room temperature. Water (2 mL) and saturated ammonium chloride solution (2 mL) were slowly added. The aqueous phase was extracted with *n*-pentane (3x 10 mL). The combined organic phases were dried over sodium sulfate and the solvent was gently removed, yielding benzyl alcohol [^{18}O] (290 mg, 49%) as colorless liquid.

^{1}H NMR (400 MHz, $CDCl_3$, δ): 7.37-7.35 (m, *4*H), 7.34 – 7.27 (m, 1H), 4.70 (d, *J* = 5.8 Hz, 2H), 1.63 (t, *J* = 5.9 Hz, 1H) ppm. **^{13}C{^{1}H} NMR** (100 MHz, $CDCl_3$, δ): 141.0, 128.7, 127.8, 127.1, 65.5 ppm.

The ^{18}O-content was assigned by mass spectrometry, using EI-MS from GC-MS data.[22]

Calculation: (Signal of m/z 110) / [(Signal of m/z 110) + (Signal of m/z 108)]:
70.02 / (70.02 + 9.93) = 88 % ^{18}O in benzyl alcohol

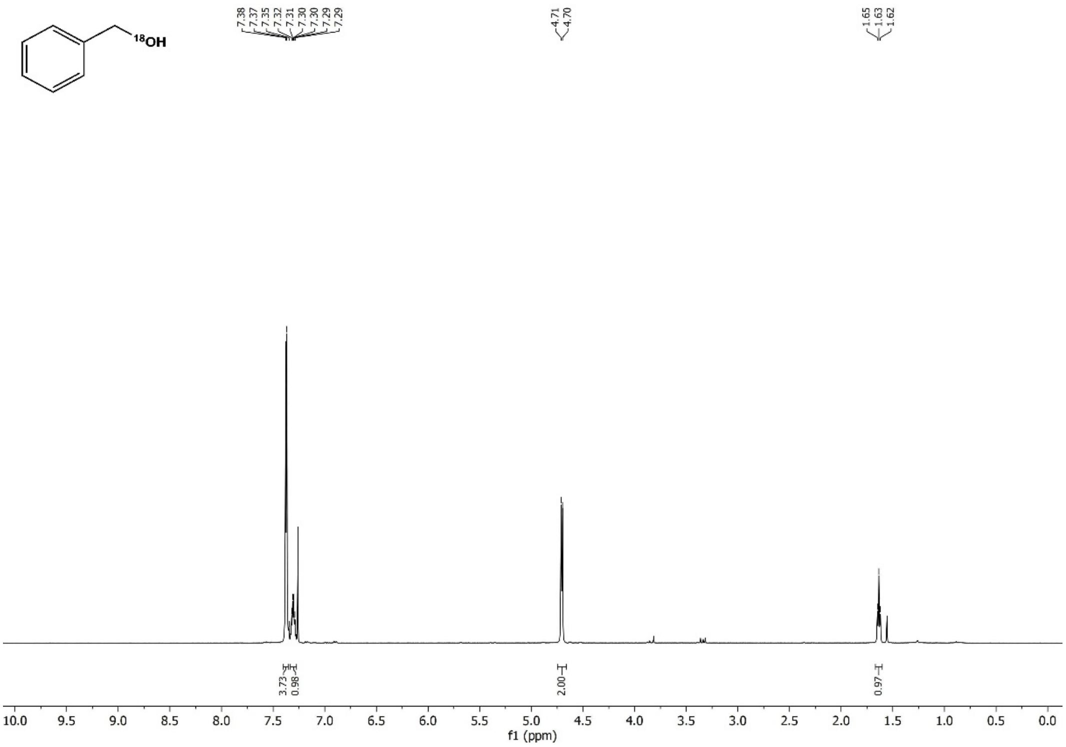

Figure S1 ^{1}H NMR spectrum of benzyl alcohol [^{18}O].

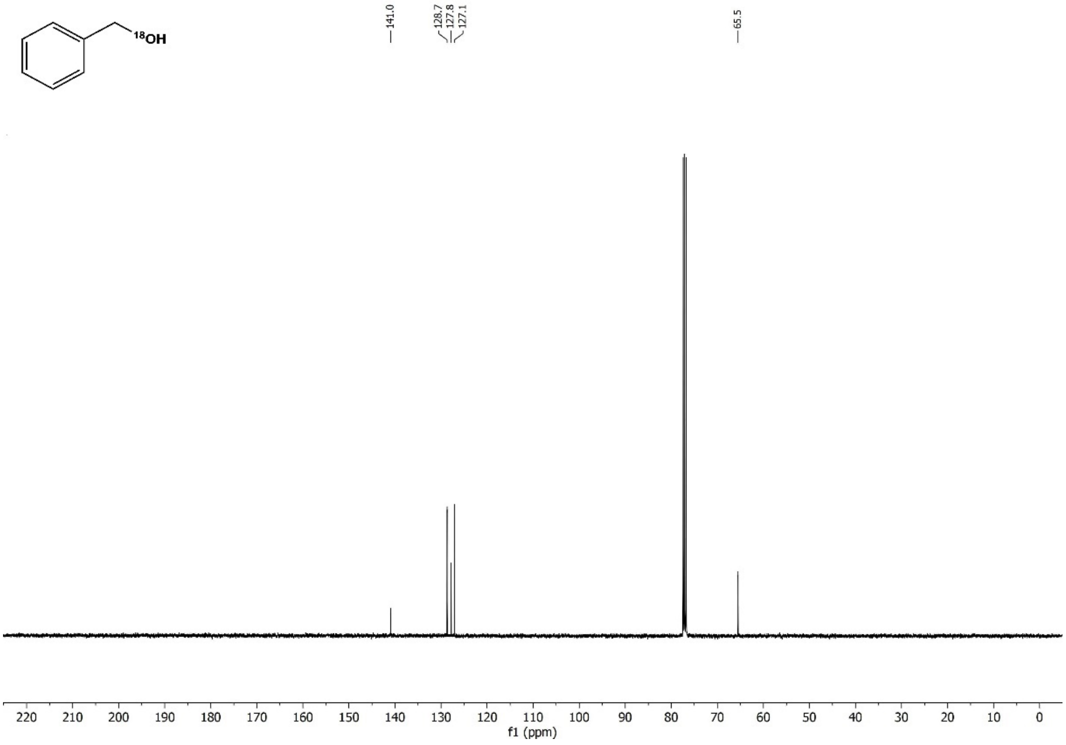

Figure S2 $^{13}C\{^{1}H\}$ NMR spectrum of benzyl alcohol [^{18}O].

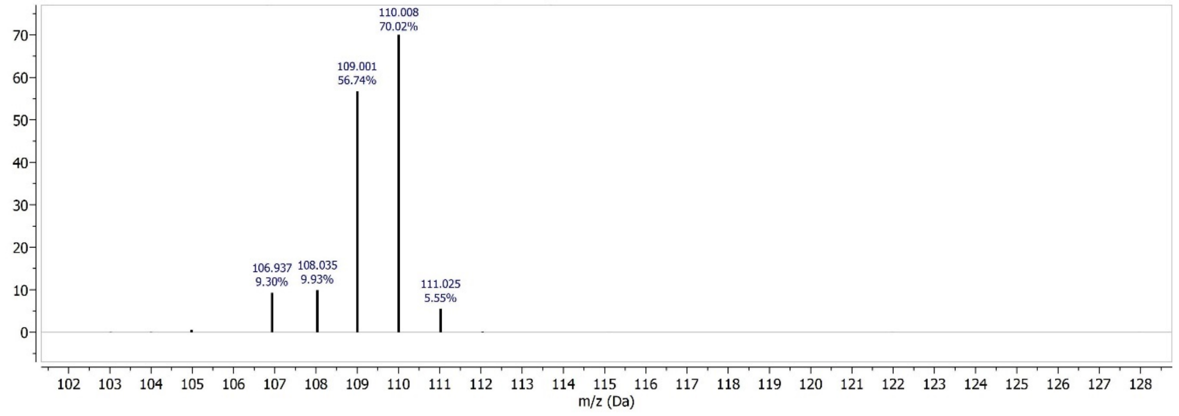

Figure S3 EI-MS spectrum of benzyl alcohol [^{18}O].

Synthesis of potassium benzylate ($PhCH_2OK$)

In an Argon filled glovebox benzyl alcohol (1.5 mL, 14.4 mmol) was reacted neat with metal K (0.5 g). The reaction mixture was stirred for 6 h at room temperature, and the solidified residue washed twice with pentane (3 mL). The off-white solid was dried in vacuum. Yield: 1.72 g (81 %)

^{1}H NMR (250 MHz, THF-d8): 7.37 (d, J = 7.2 Hz, 2H), 7.21 (t, J = 7.3 Hz, 2H), 7.09 (t, J = 7.2 Hz, 1H), 4.75 (s, 2H).

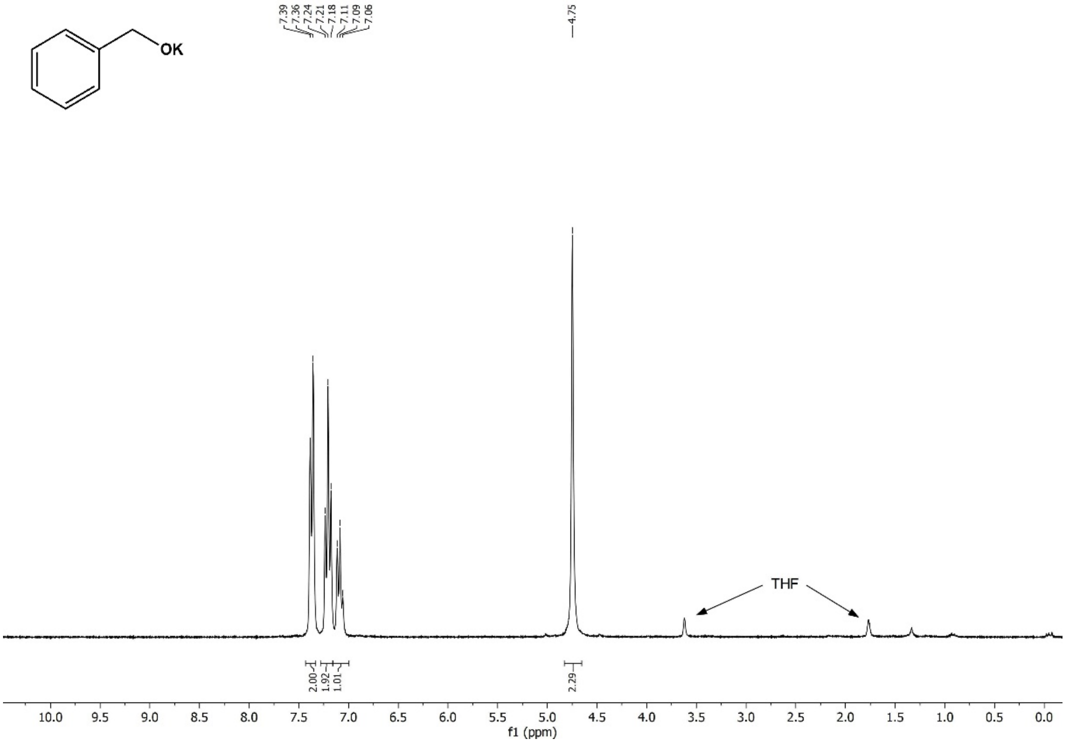

Figure S4 ^{1}H NMR of potassium benzylate.

N-Alkylation of aniline with benzyl alcohol [^{18}O]

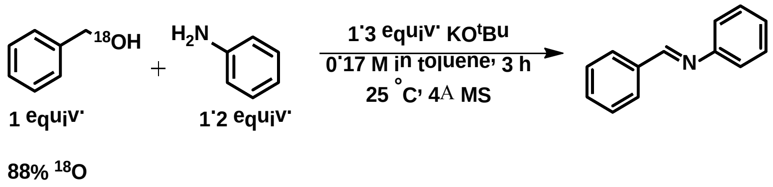

The reaction was carried out according to the procedure described above, employing benzyl alcohol [^{18}O] instead of standard benzyl alcohol. Samples for GC-MS analysis were taken every 15 min. The ^{18}O content was determined by EI-MS.

20±2% ^{18}O content was found in *in situ* formed benzaldehyde upon reaction progress.

N-Alkylation of aniline using PhCH$_2$OK salt

The reaction was carried out according to the procedure described above, employing the potassium benzylate salt instead of standard benzyl alcohol and KO^{t}Bu. The manipulation of reactants was carried out in a glovebox, whereas the reaction itself was carried out under ambient atmospheric conditions. A GC-MS analysis showed 54% conversion.

N-Alkylation of aniline with benzyl alcohol-α,α-d$_2$

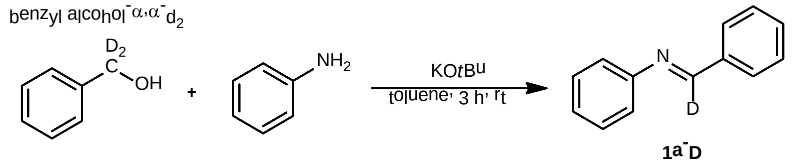

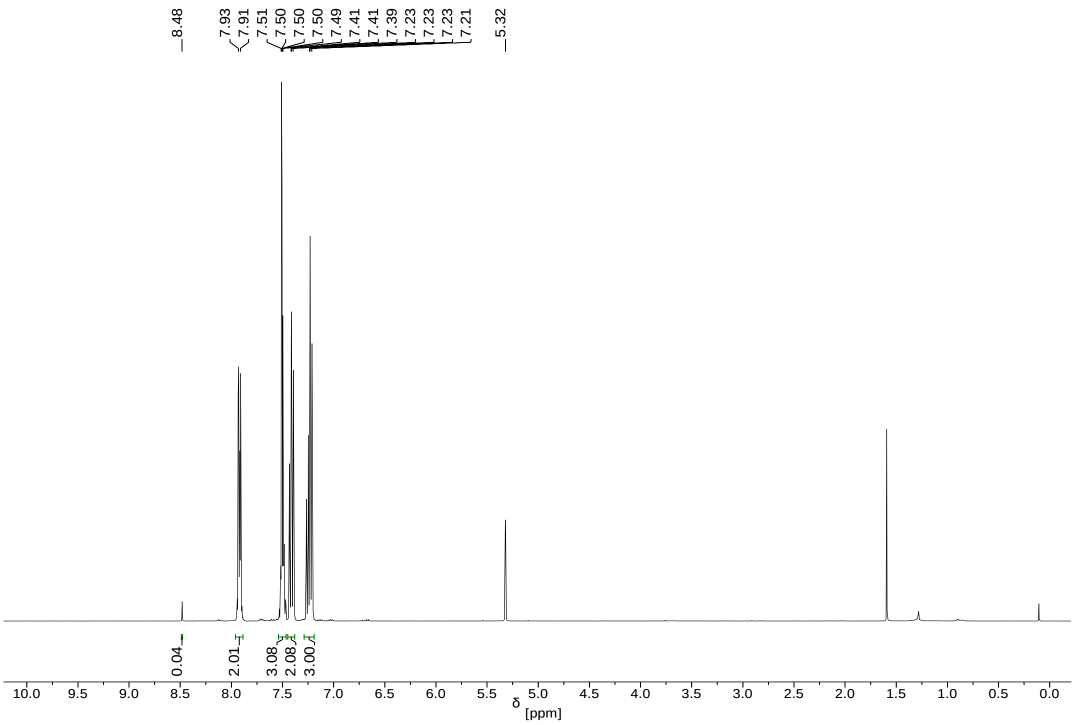

Figure S5. ^{1}H NMR of **1a-D.** Reaction conditions: aniline (55 μl, 0.6 mmol), benzyl alcohol-α,α-d2 (52 μl, 0.5 mmol, 96% D), KO^{t}Bu (73 mg, 0.65 mmol), molecular sieves (15-20 pellets, 4 Å) and toluene (3 ml), room temperature, 3 h. Work up same as **1a.**

EPR monitoring of N-Alkylation

In a glovebox, a flame-dried EPR tube was charged with a 0.5 mL of a solution of KO^{t}Bu, aniline and benzyl alcohol in toluene (prepared as described in the general procedure above) and sealed. A consecutive EPR experiment (100 K and room temperature) of this sample showed no presence of paramagnetic material. The measurement itself was repeated after 1 h with identical results.

Subsequently the air-tight sample tube was opened, and 2 mL of air was injected with a long steel canula into the solution. The sample was consecutively cooled down to 100 K and subjected to EPR measurement. A signal was detected with g_{iso} = 2.001 and hyperfine coupling A(^{1}H) = 18 G distinctive for an organic carbon-based radical.

A similar experiment was carried out with $PhCH_2OK$ salt and aniline to give identical results.

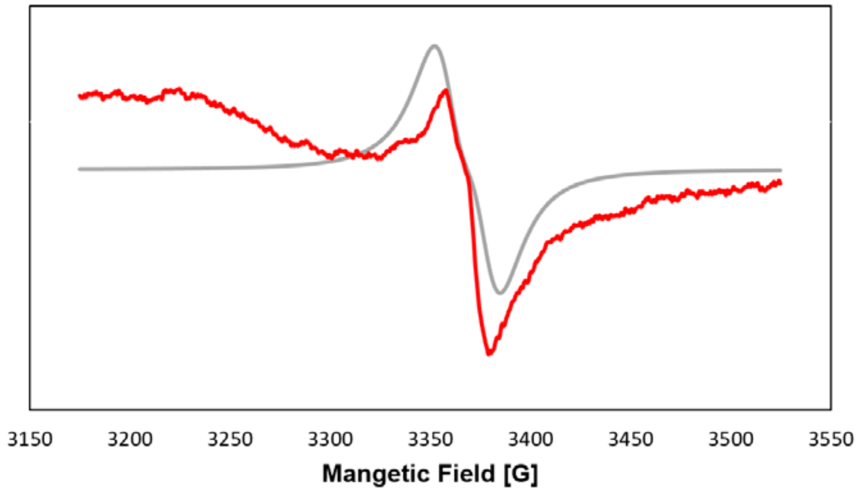

Figure S6. X-Band cw-EPR spectrum of the reaction mixture at 100 K in frozen toluene glass. The obtained signal is depicted in red and the computer simulation in grey color.

5. Computational studies

For the structure optimization, Density Functional Theory (DFT) was the method of choice, using the PBE0[23a] functional, def2-TZVP[23b] basis set with D4[23c] dispersion corrections as well as implicit solvation via the CPCM[23d] implementation (toluene was used as solvent). All calculations were performed using the ORCA 5.0.3[23e,f] software suite. To validate the accuracy of the DFT method, structures **A** and **D** were optimized using domain based local pair-natural orbital (DLPNO) based singles- and doubles, and perturbative triples coupled cluster (CCSD(T))[23g] with the def2-TZVP basis set an the CPCM implicit solvent model and minimal differences in the geometry were observed. The energetics confirmed this, as the energy change between single point calculations performed at the DLPNO-CCSD(T)/def2-QZVP[23b]/CPCM level on both DFT and CCSD optimized structure was less than 0.25 kcal/mol. To speed up calculation coulomb fitting was used.[23h,i]

By performing single point calculations using B2PLYLP[23i]/def2-TZVP/D4/CPCM on the structures and comparing them to CCSD values, we observed very small energy differences of up to 3 kcal/mol. As it is known that CCSD does not handle mixed spin states well, it was unfeasible to obtain accurate energy values for the minimum energy crossing points (MECP). As such, the DFT double hybrid values were taken for the energy barriers related to these spin-transition points along the reaction pathway. The MECP structures were calculated using a method by Harvey *et al as* implemented in ORCA.[24] Here, both the energy of spin surface 1 and the energy difference between the two spin surfaces are minimized. The structures were verified to be true minimum geometries by running surface crossing adapted frequency calculations, which showed no negative values.

The full proposed mechanism is depicted in Figure S7, with a multitude of conditional branching points, depending on the immediate environment of the molecule. While the mechanism itself is not exhaustive, it can provide greater insight into the possible species formed and their interactions.

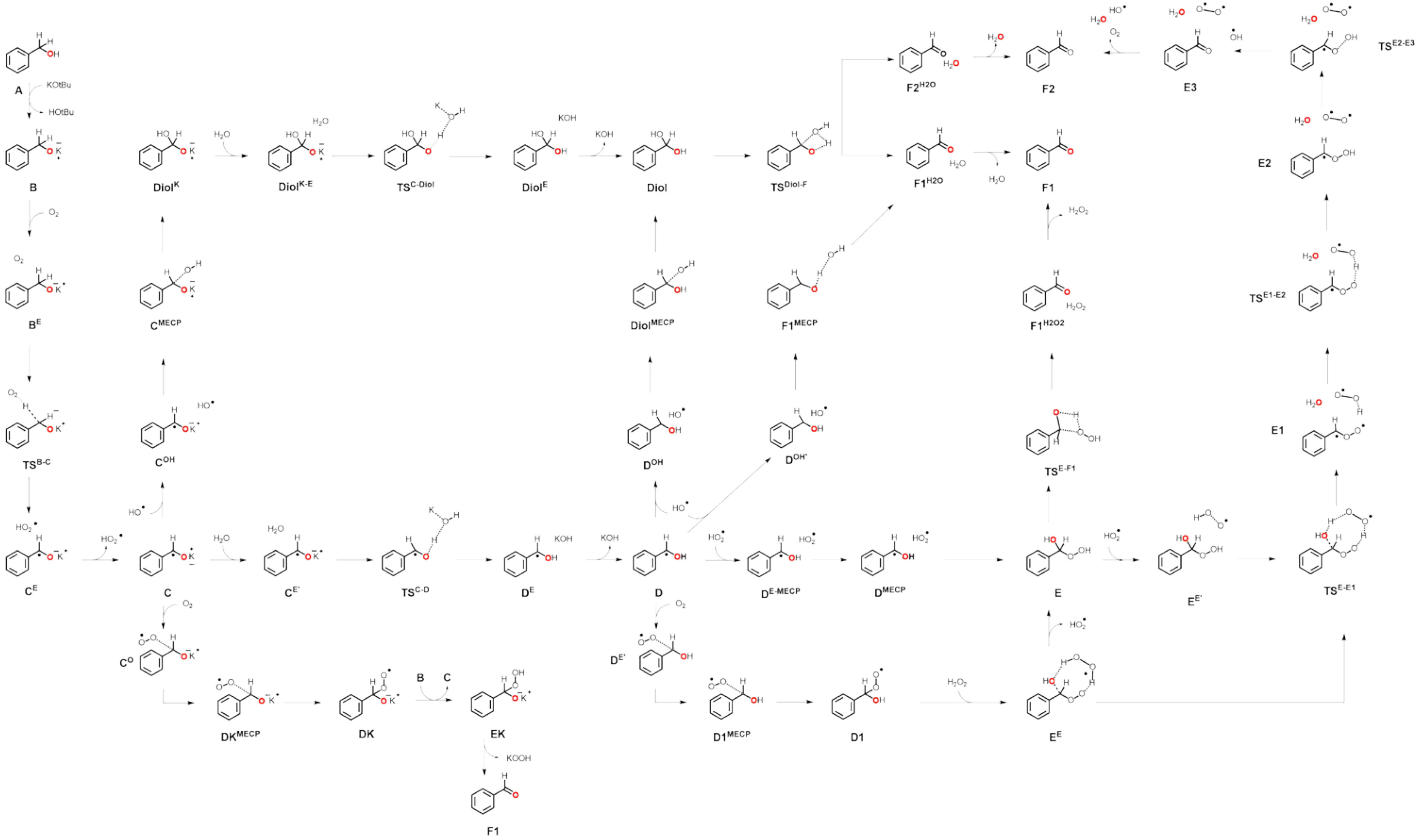

Figure S7. Reaction network of the oxidation of benzylalcohol to benzaldehyde.

Figure S8 depicts the reaction pathways C to E that depend on the existence of the short-lived hydroxyl radical and are therefore less likely to take place, even though the calculated thermodynamics and kinetics suggest that they are viable.

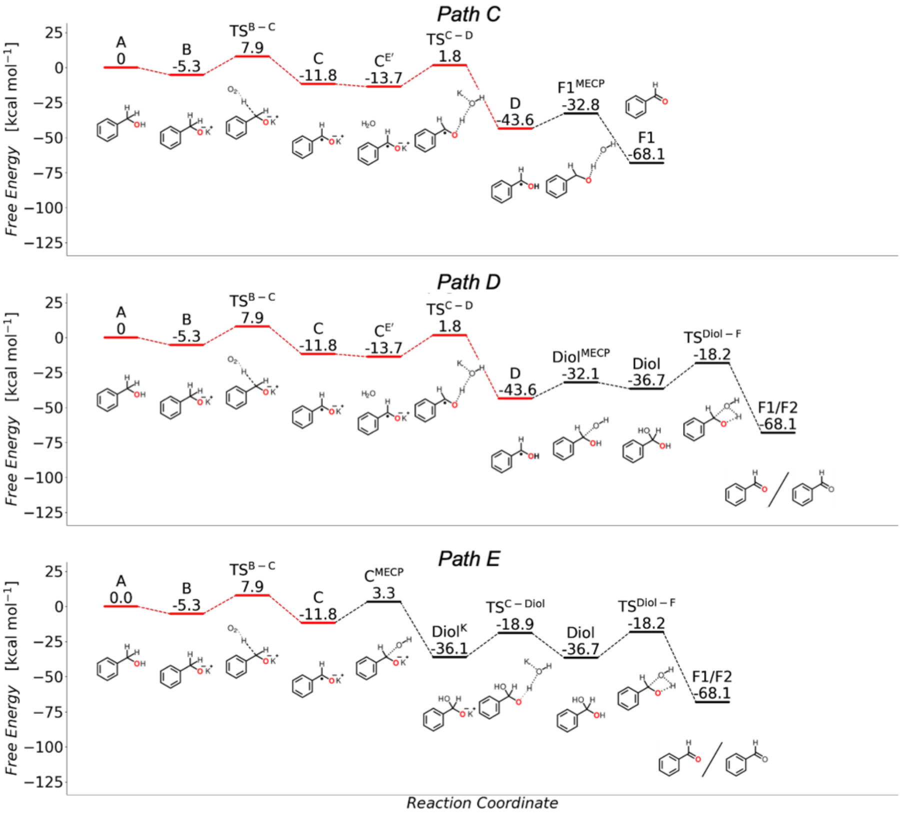

Figure S8. Pathways C-E of the water-assisted mechanism, branching out from C and D respectively. As these pathways depend on the presence of a short-lived hydroxyl radical, they are less likely to take place. All values are free energies ΔG, in kcal/mol, calculated with DLPNO-CCSD(T)/def2-QZVP//PBE0/def2-TZVP/D4. The ^{18}O-label is depicted in red.

For the EPR calculations, the method of choice was B2PLYP/def2-TZVP/D4/CPCM, using gauge independent atomic orbitals. We performed calculations on all intermediate radicals, and **C**/**D** have the g_{iso} values closest to the experimental results. Simulations of the EPR spectra were performed with Easyspin[25] and do not provide clear evidence whether **C** or **D** is the experimentally observed radical species or a combination of both as a small shoulder is visible in the experimental spectra, compare Figures S6 and S9.

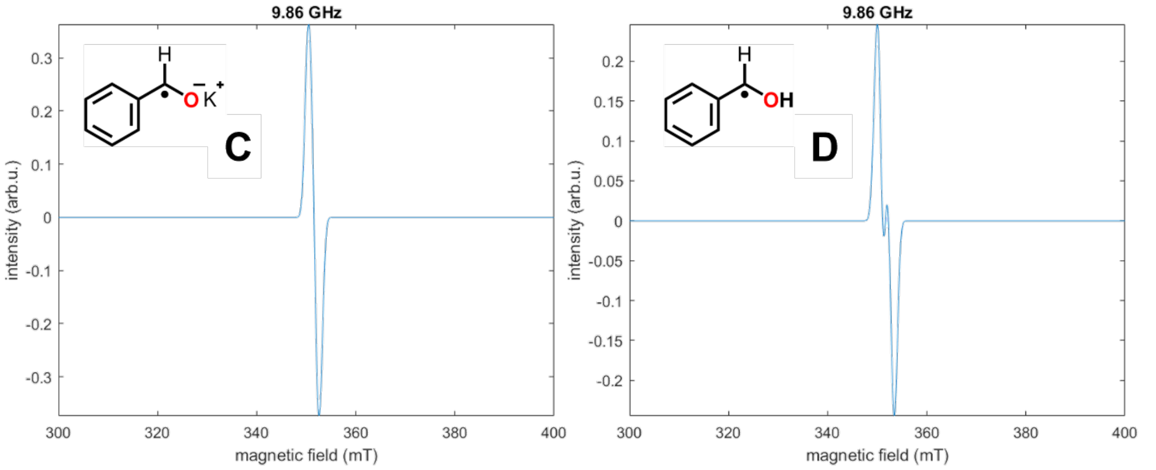

Figure S9. Simulated EPR spectrum at 100 K. Values obtained from B2PLYLP/def2-TZVP/D4/CPCM calculations using gauge independent atomic orbitals.

6. NMR Spectra of Imines Products

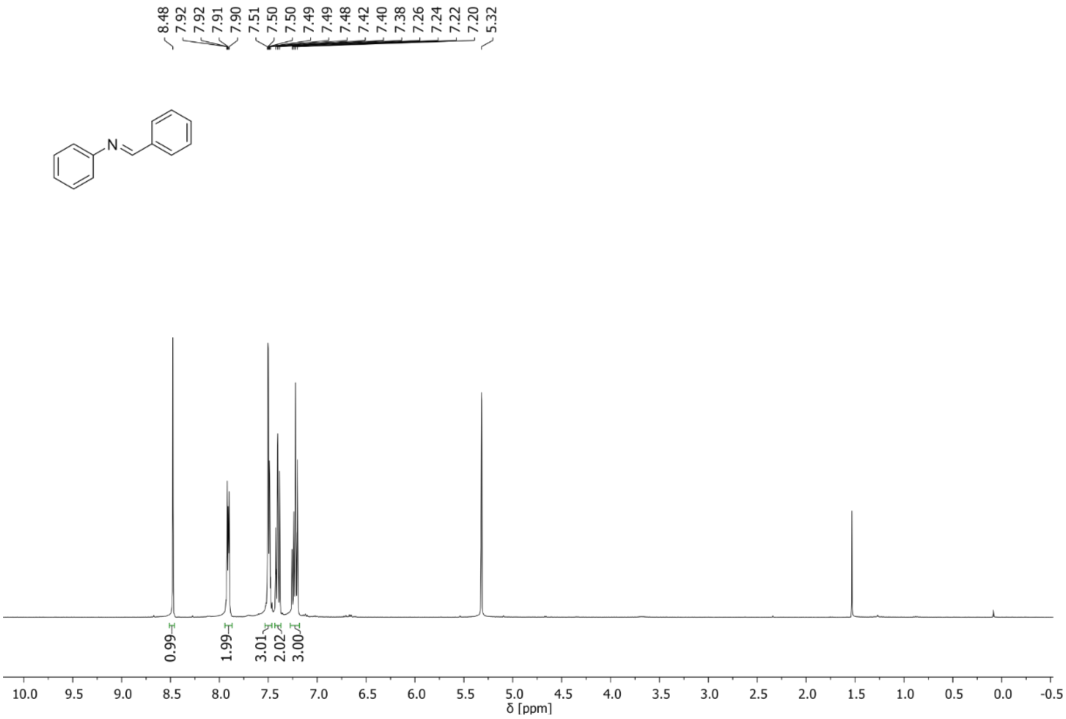

^{1}H NMR: (E)-N-benzylideneaniline (**1a**)

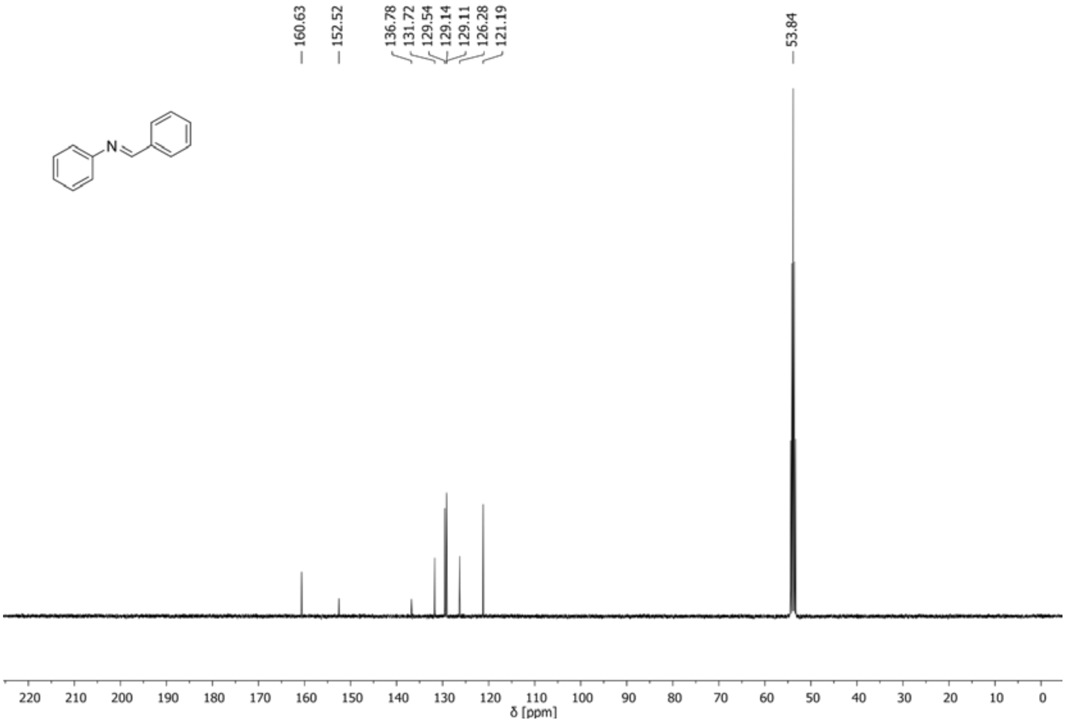

$^{13}C\{^{1}H\}$ NMR: (E)-N-benzylideneaniline (**1a**)

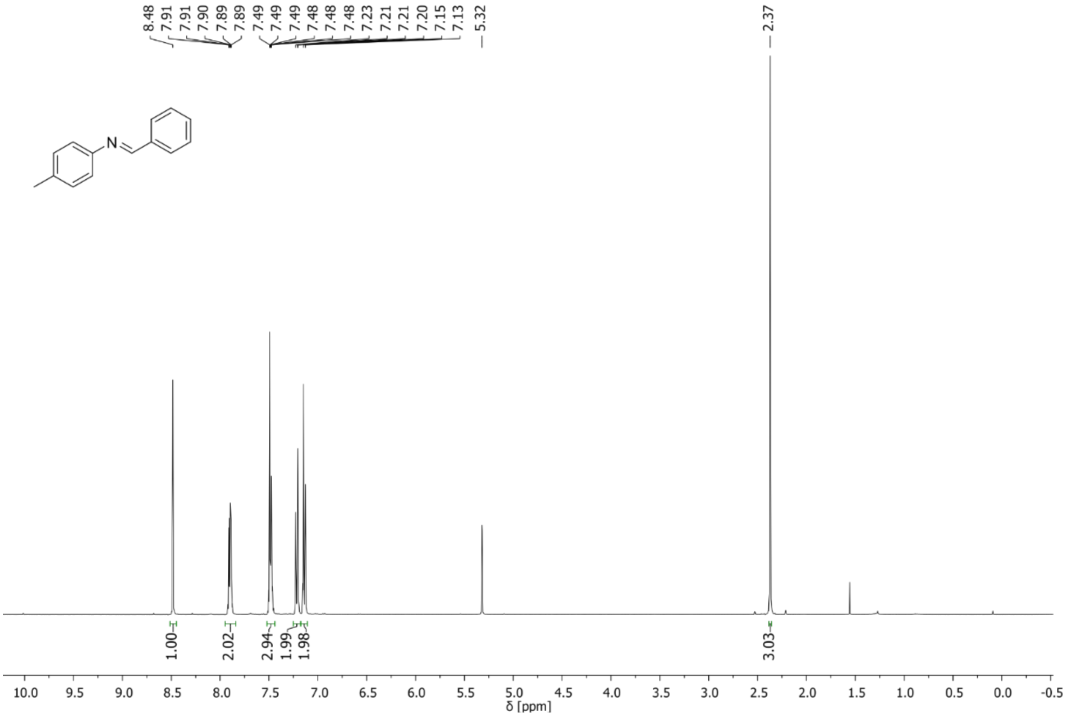

^{1}H NMR: (E)-1-phenyl-N-(p-tolyl)methanimine (**1b**)

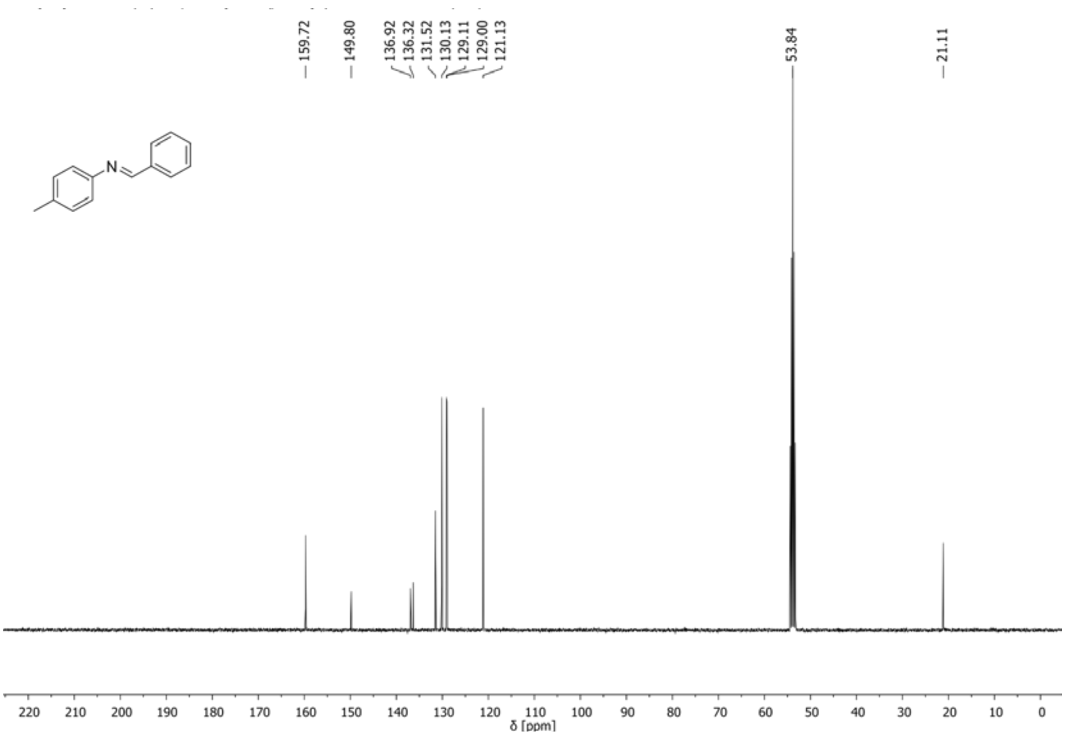

$^{13}C\{^{1}H\}$ NMR: (E)-1-phenyl-N-(p-tolyl)methanimine (**1b**)

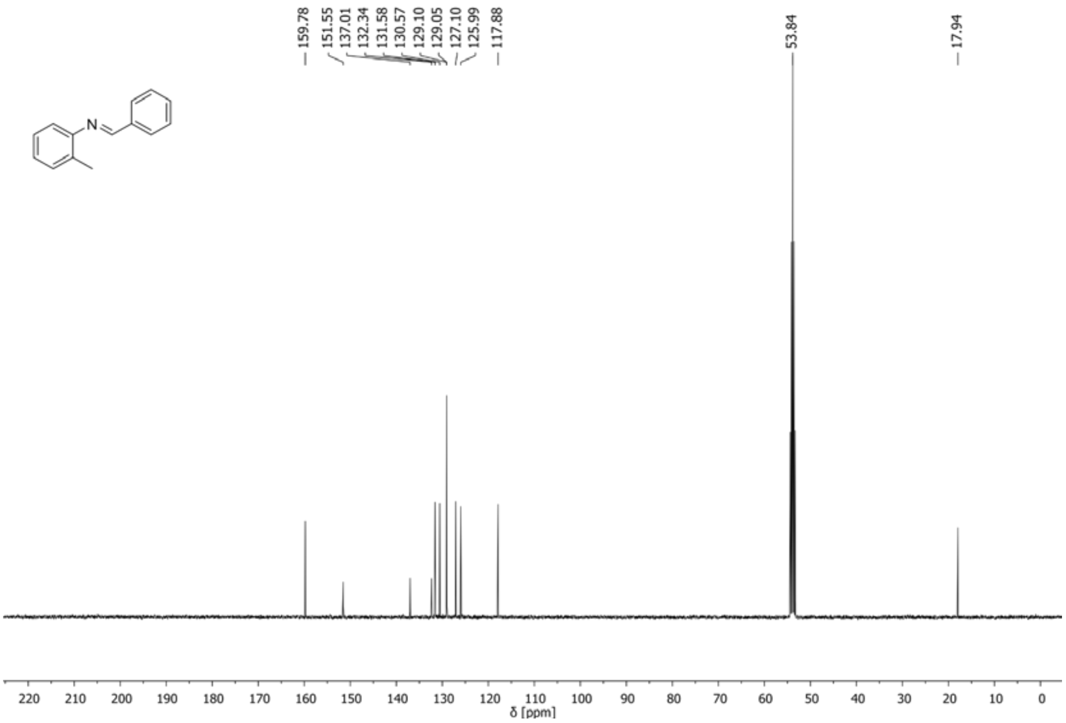

^{1}H NMR: (E)-1-phenyl-N-(o-tolyl)methanimine (**1c**)

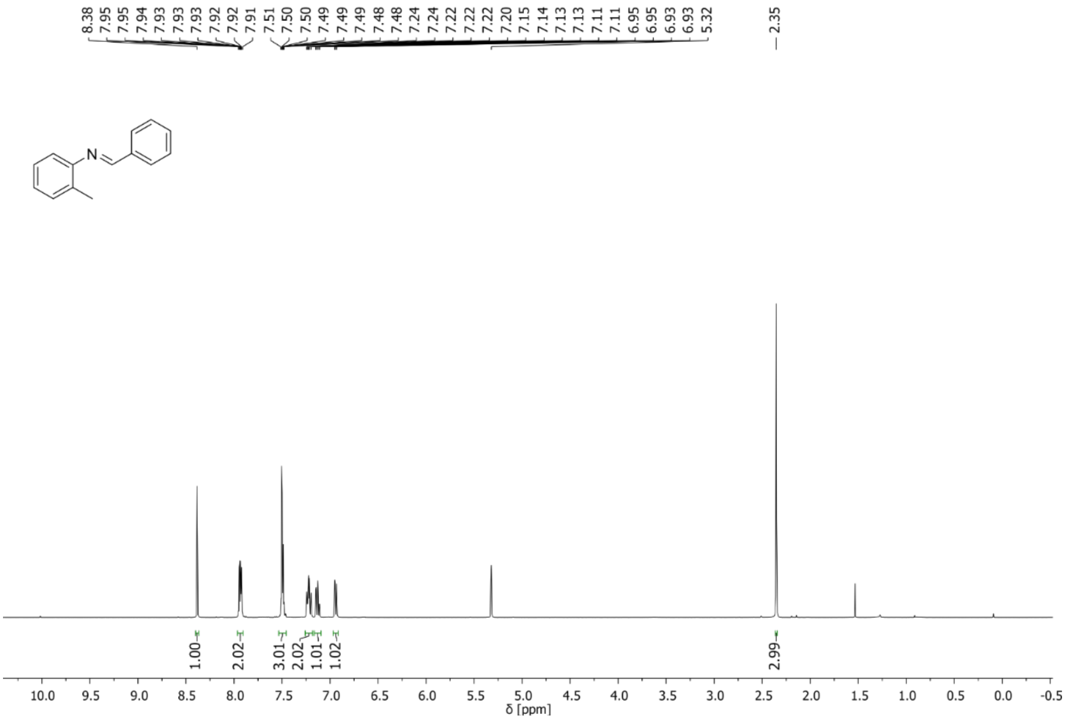

$^{13}C\{^{1}H\}$ NMR: (E)-1-phenyl-N-(o-tolyl)methanimine (**1c**)

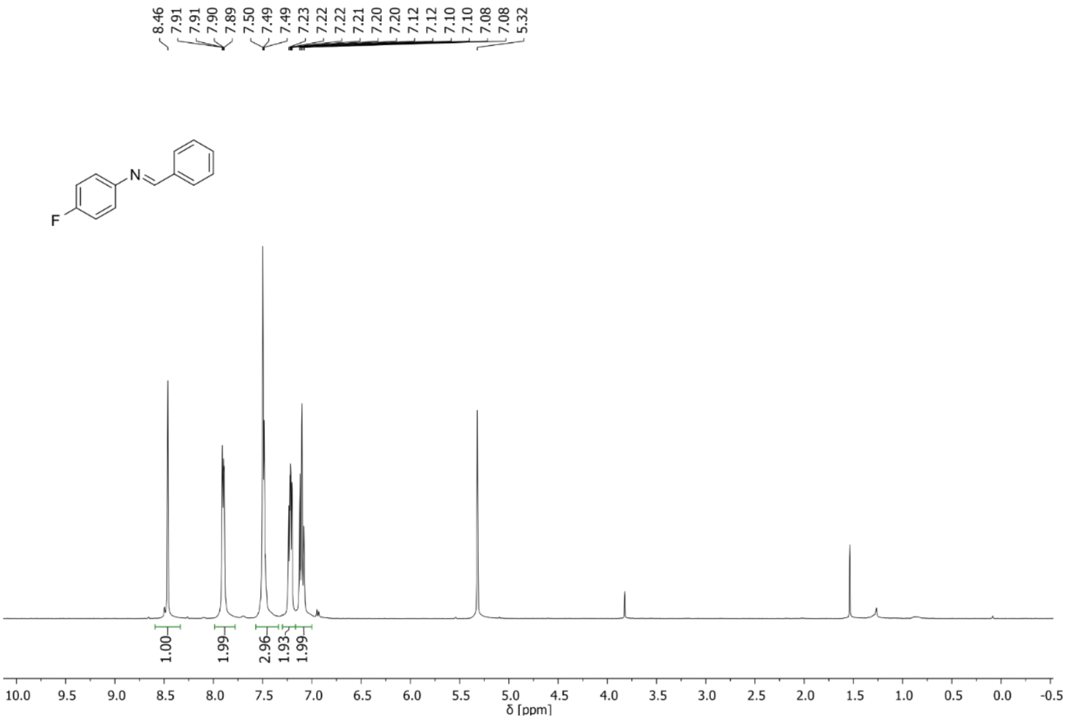

^{1}H NMR: (E)-N-benzylidene-4-fluoroaniline (**1d**)

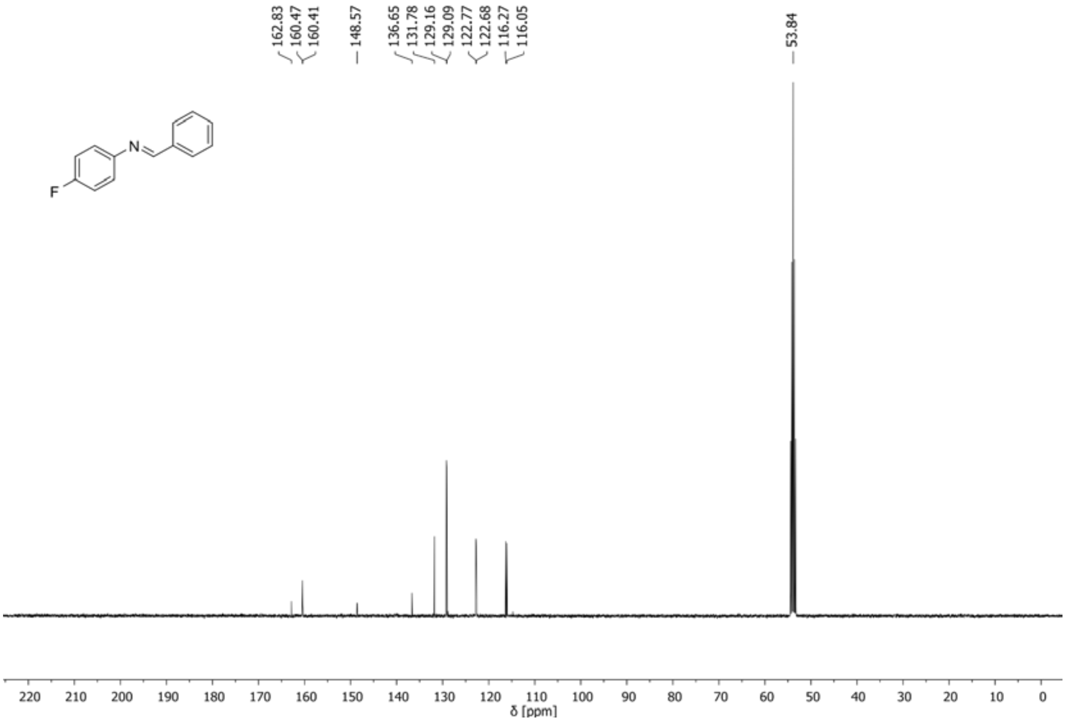

$^{13}C\{^{1}H\}$ NMR: (E)-N-benzylidene-4-fluoroaniline (**1d**)

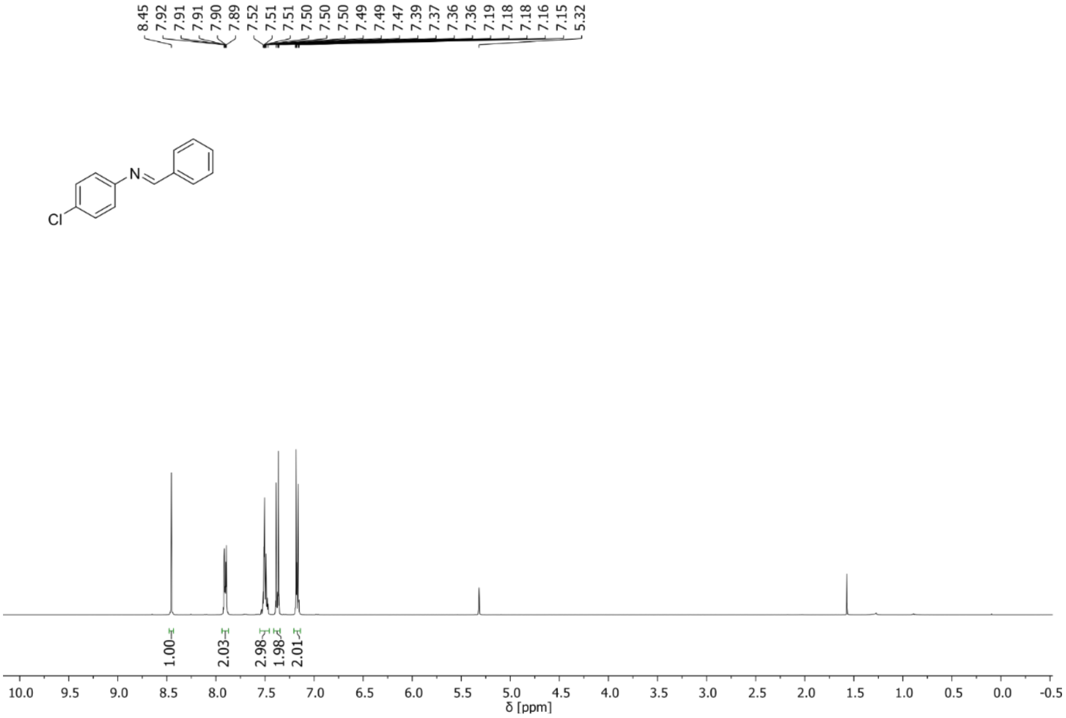

^{1}H NMR: (E)-N-benzylidene-4-chloroaniline (**1e**)

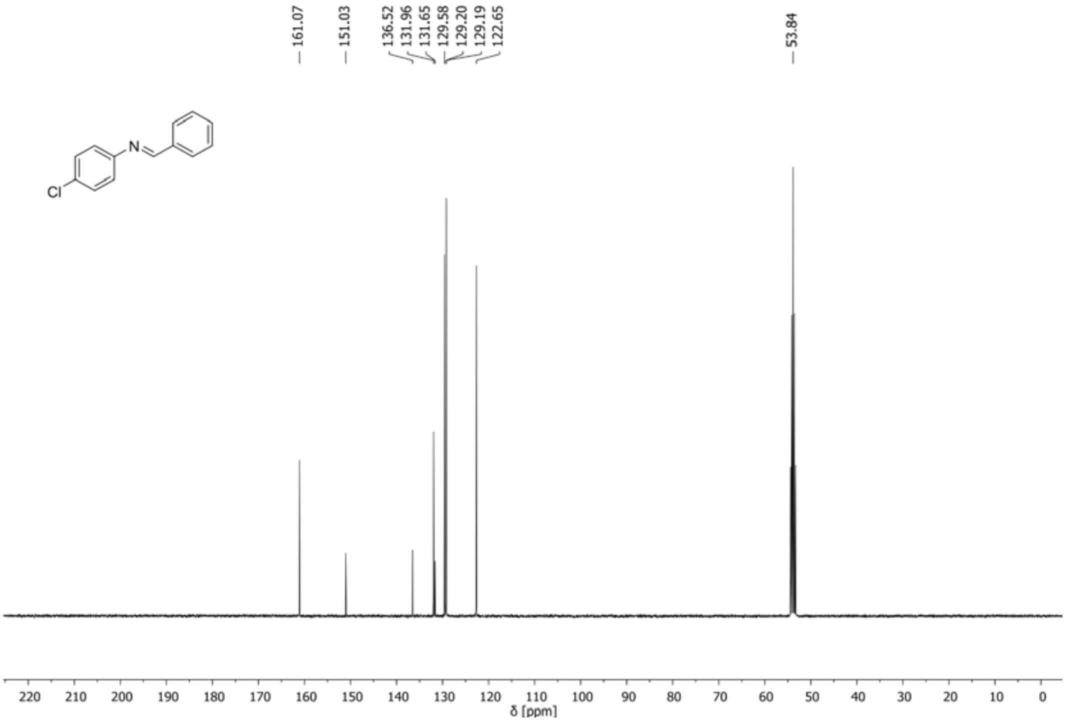

$^{13}C\{^{1}H\}$ NMR: (E)-N-benzylidene-4-chloroaniline (**1e**)

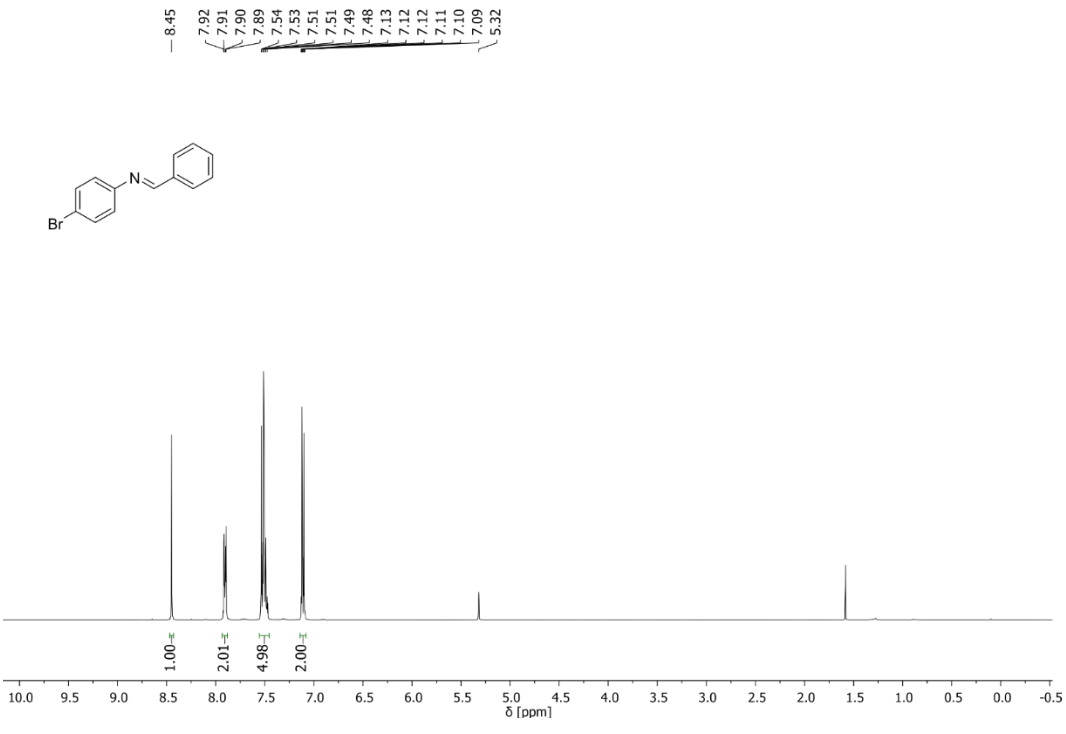

^{1}H NMR: (E)-N-benzylidene-4-bromoaniline (**1f**)

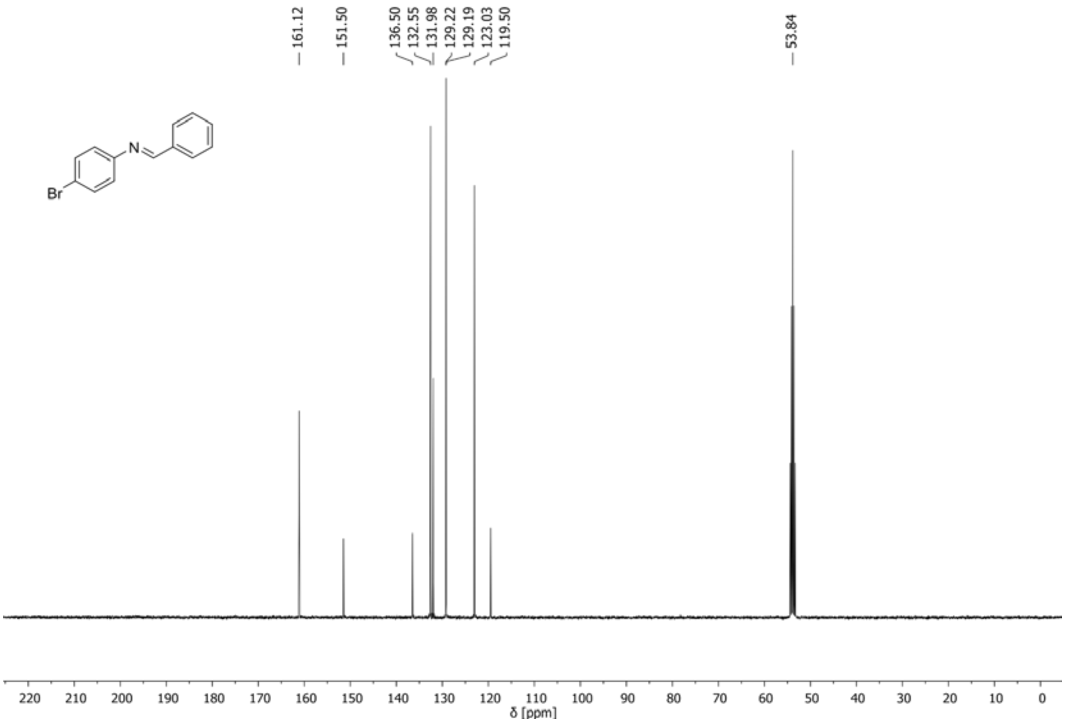

$^{13}C\{^{1}H\}$ NMR: (E)-N-benzylidene-4-bromoaniline (**1f**)

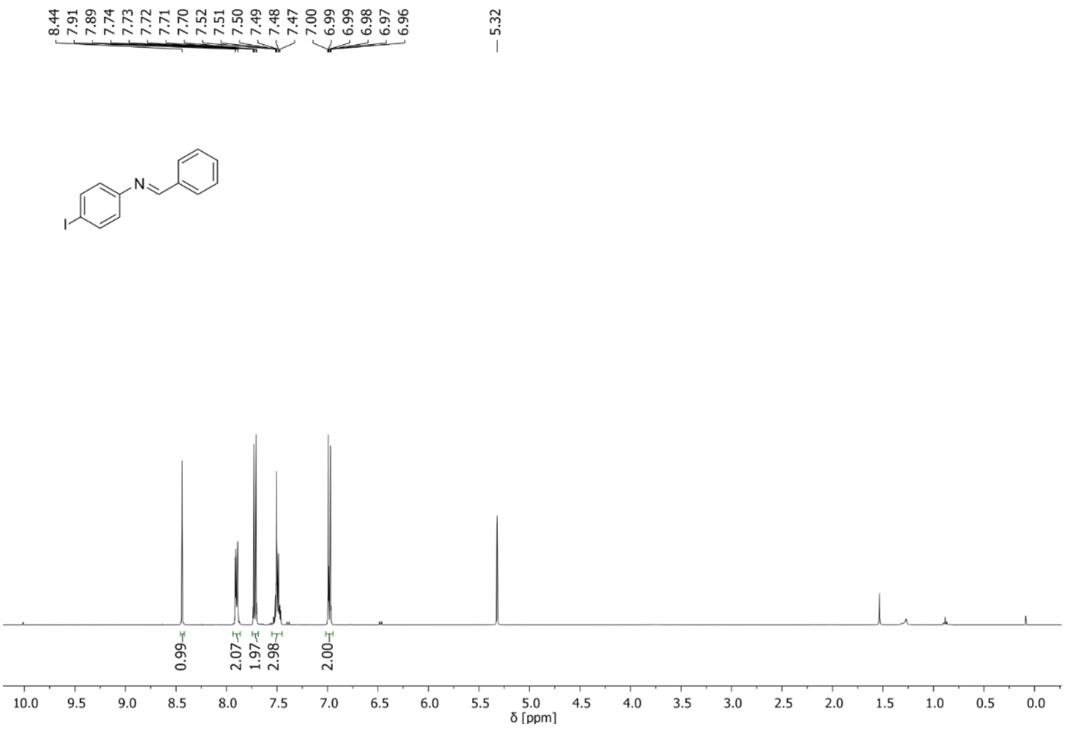

^{1}H NMR: (E)-N-benzylidene-4-iodoaniline (**1g**)

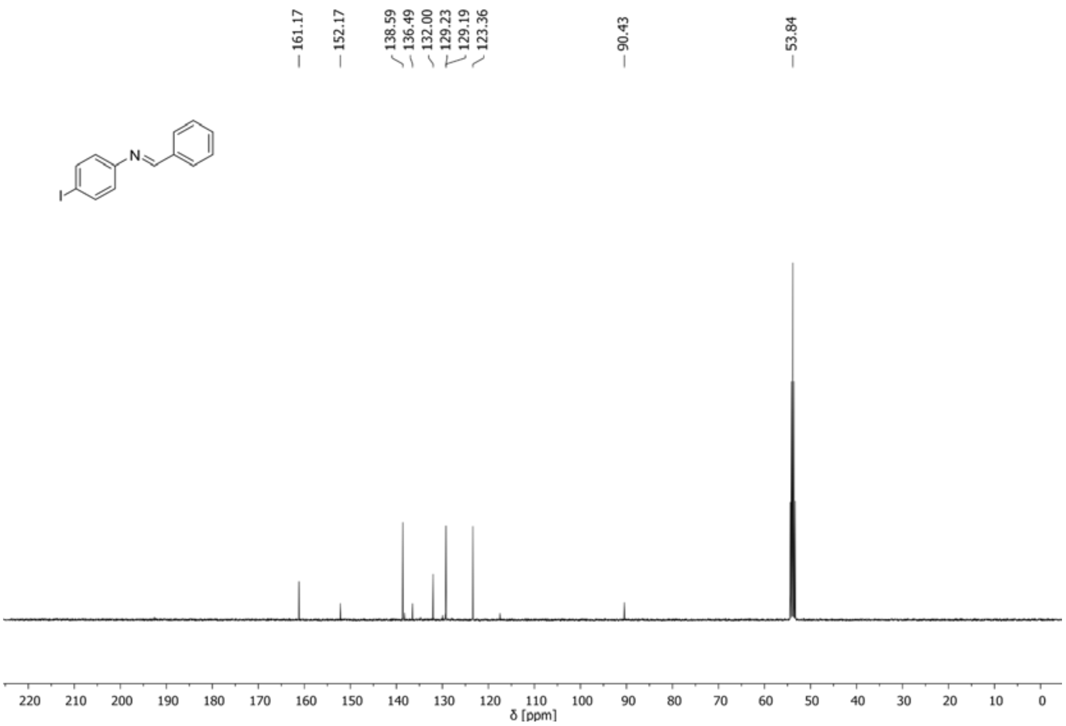

$^{13}C\{^{1}H\}$ NMR: (E)-N-benzylidene-4-iodoaniline (**1g**)

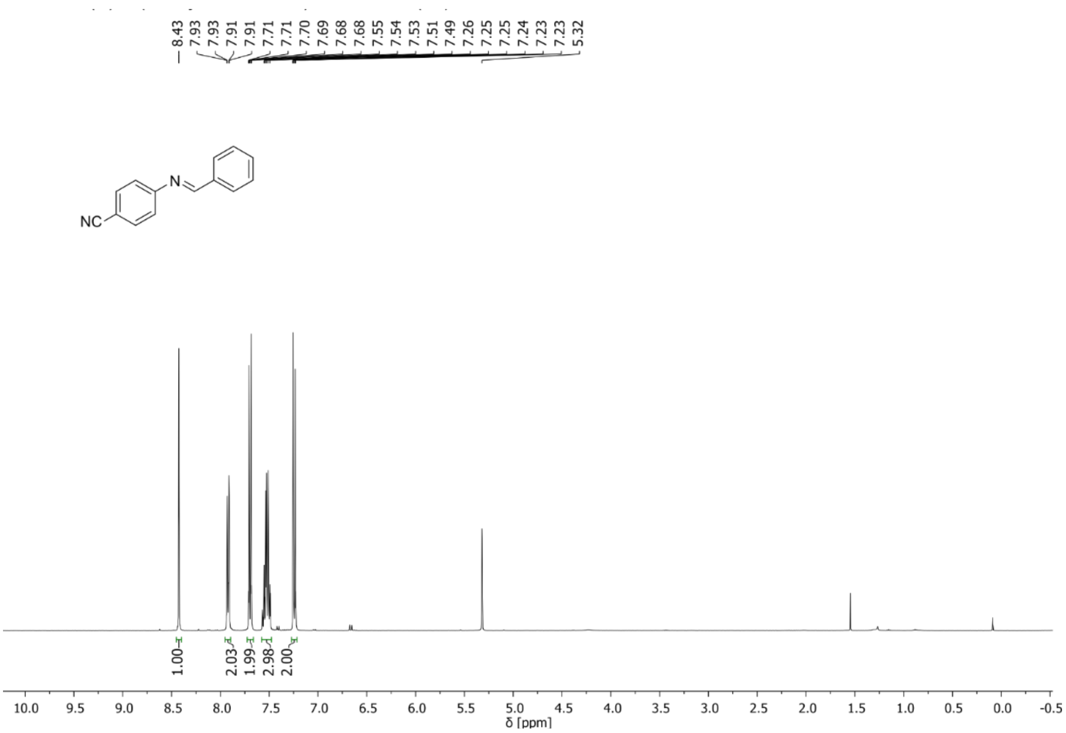

^{1}H NMR: (E)-4-(benzylideneamino)benzonitrile (**1h**)

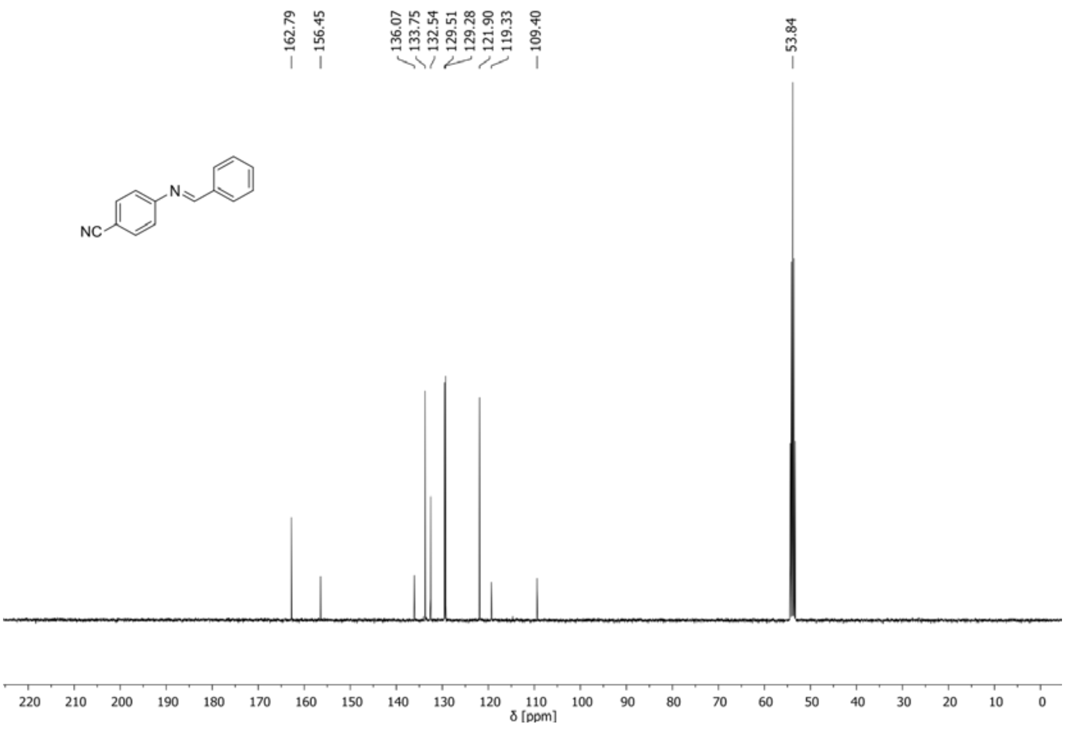

$^{13}C\{^{1}H\}$ NMR: (E)-4-(benzylideneamino)benzonitrile (**1h**)

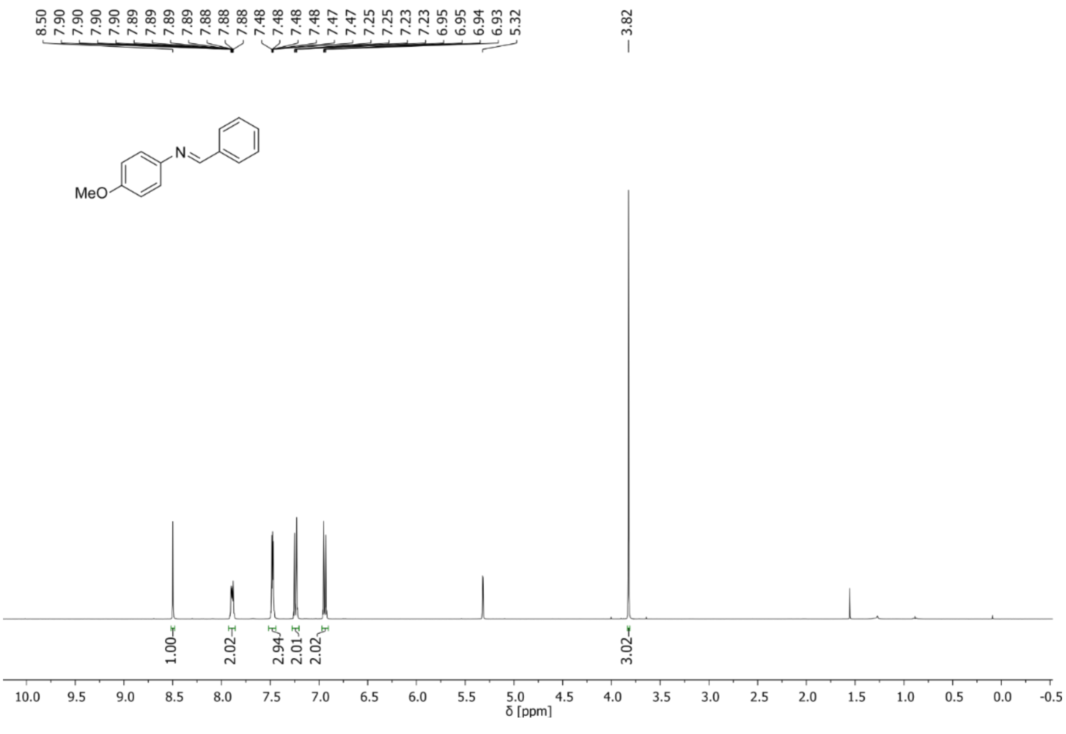

^{1}H NMR: (E)-N-benzylidene-4-methoxyaniline (**1i**)

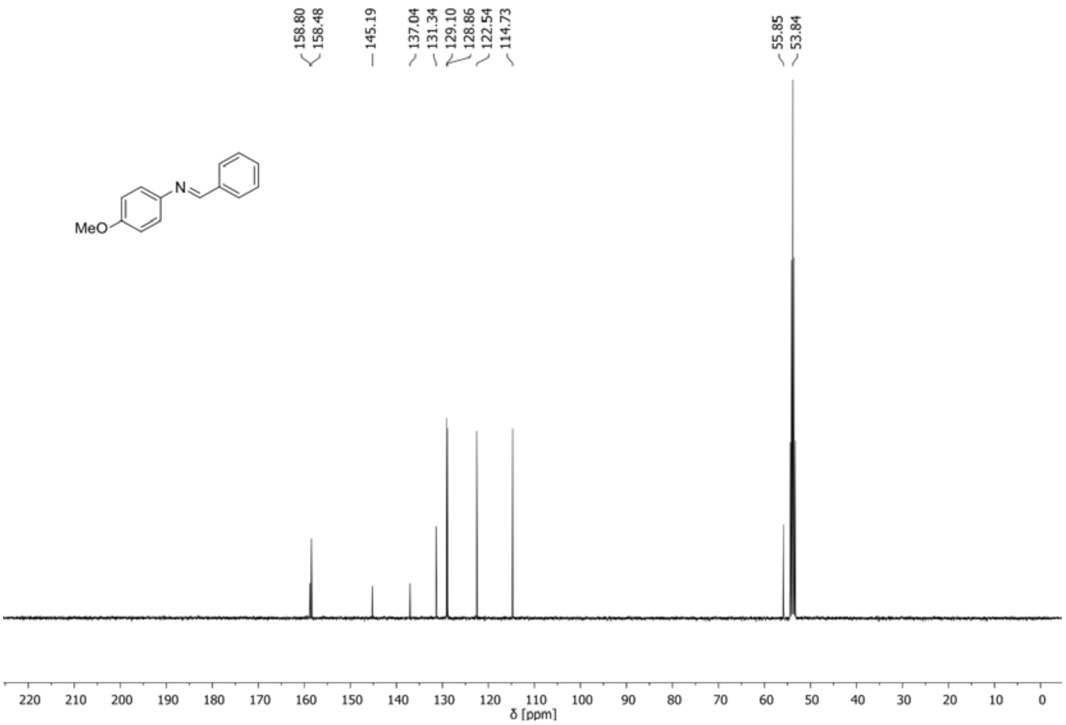

$^{13}C\{^{1}H\}$ NMR: (E)-N-benzylidene-4-methoxyaniline (**1i**)

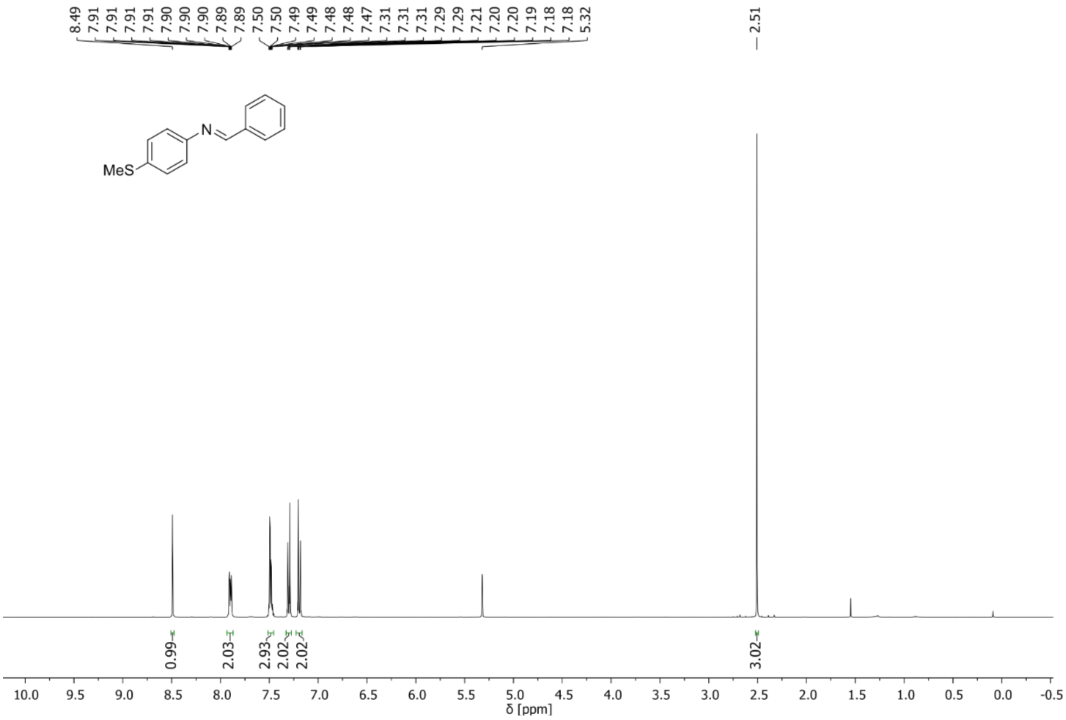

^{1}H NMR: (E)-N-benzylidene-4-(methylthio)aniline (**1j**)

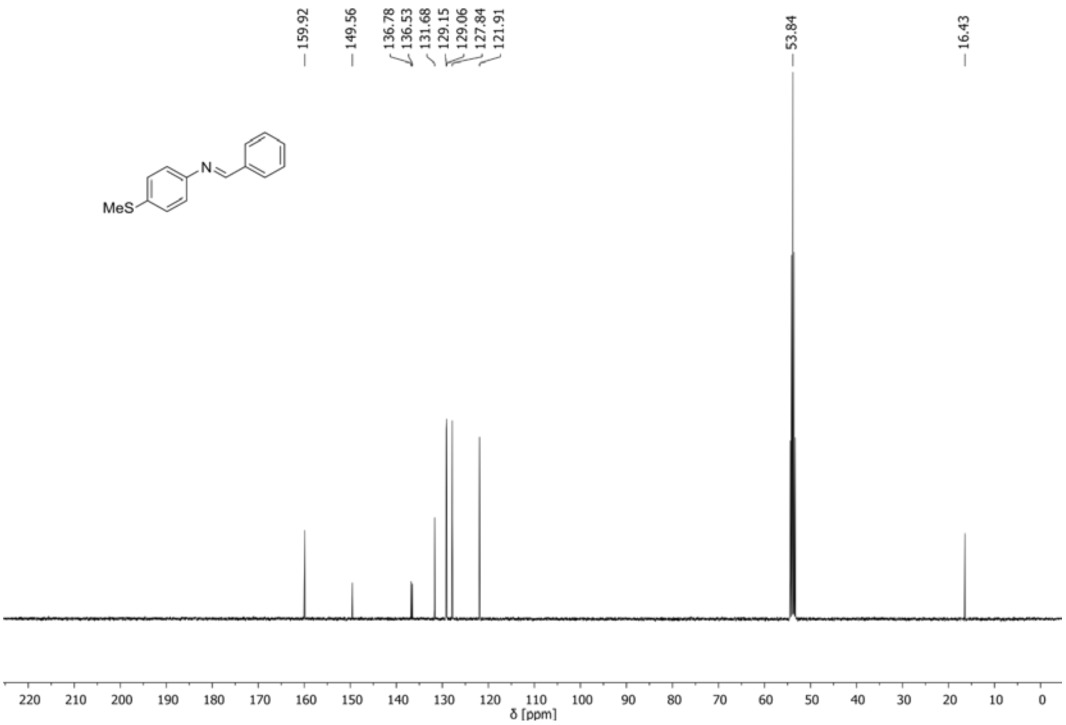

$^{13}C\{^{1}H\}$ NMR: (E)-N-benzylidene-4-(methylthio)aniline (**1j**)

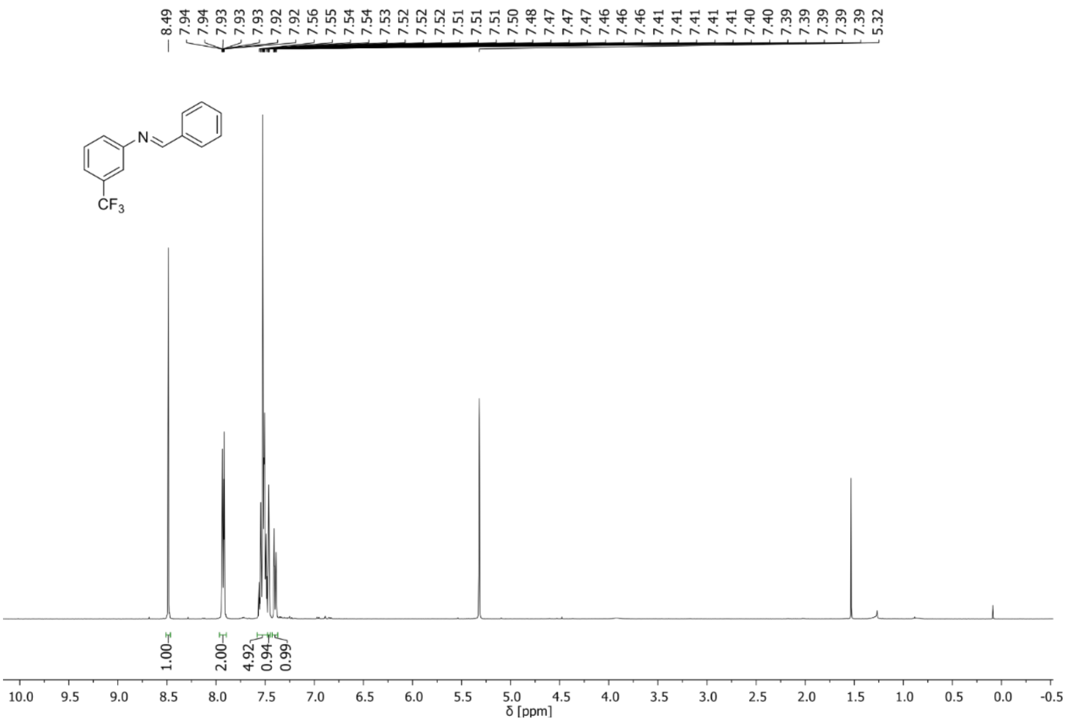

^{1}H NMR: (E)-N-benzylidene-3-(trifluoromethyl)aniline (**1k**)

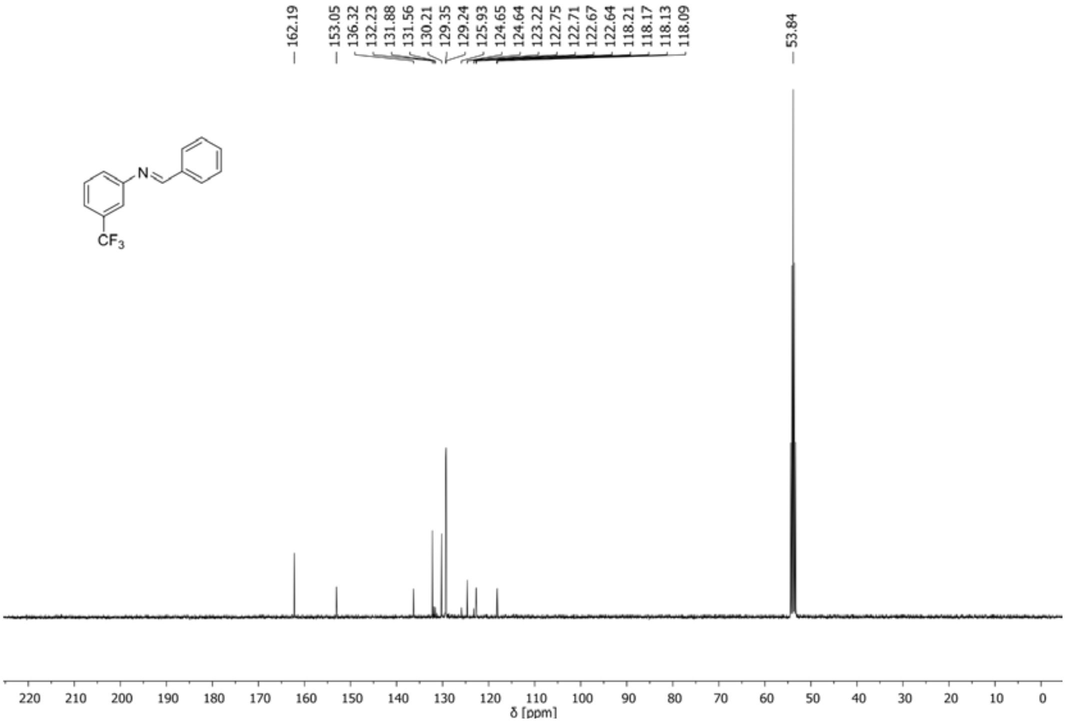

$^{13}C\{^{1}H\}$ NMR: (E)-N-benzylidene-3-(trifluoromethyl)aniline (**1k**)

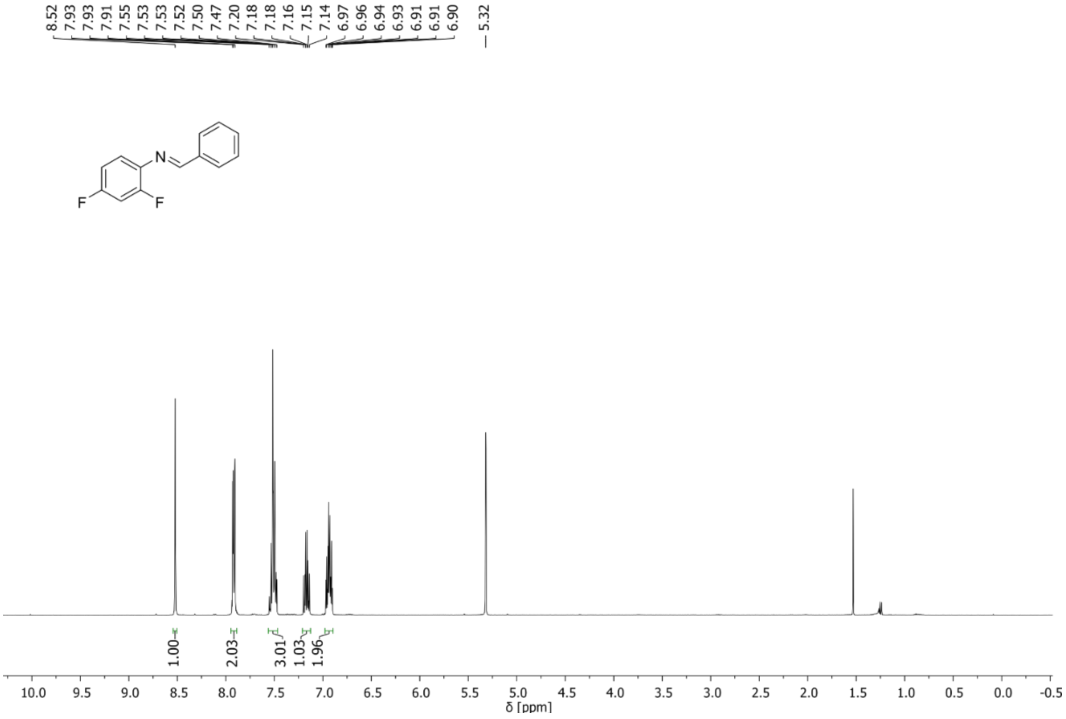

^{1}H NMR: (E)-N-benzylidene-2,4-difluoroaniline (**1l**)

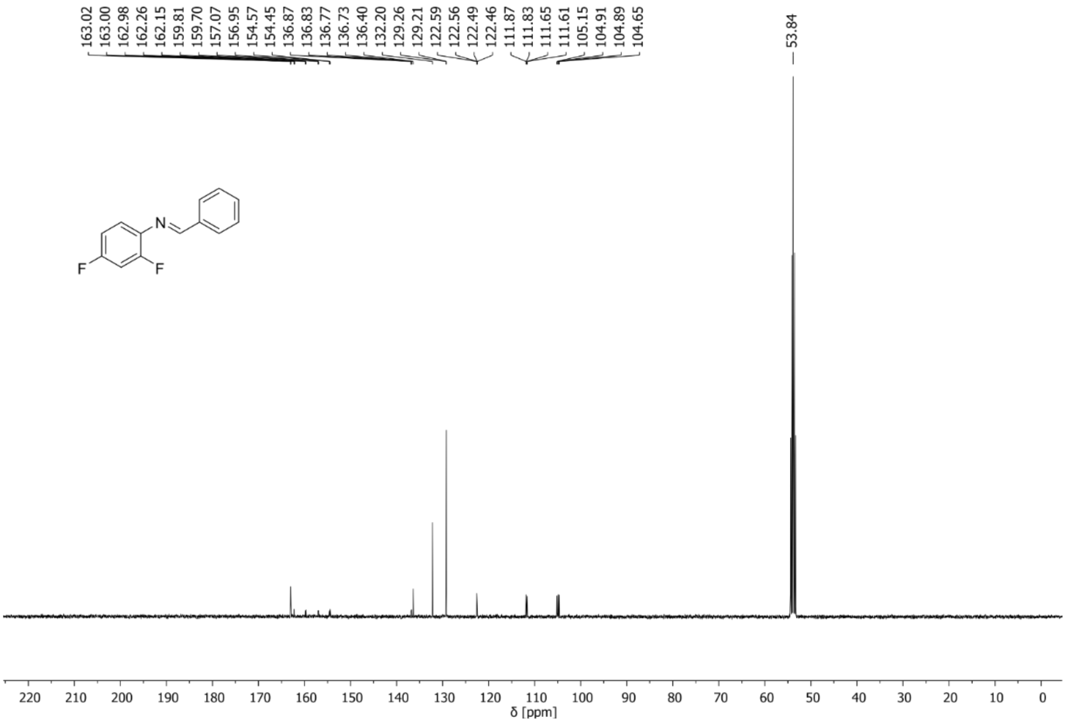

$^{13}C\{^{1}H\}$ NMR: (E)-N-benzylidene-2,4-difluoroaniline (**1l**)

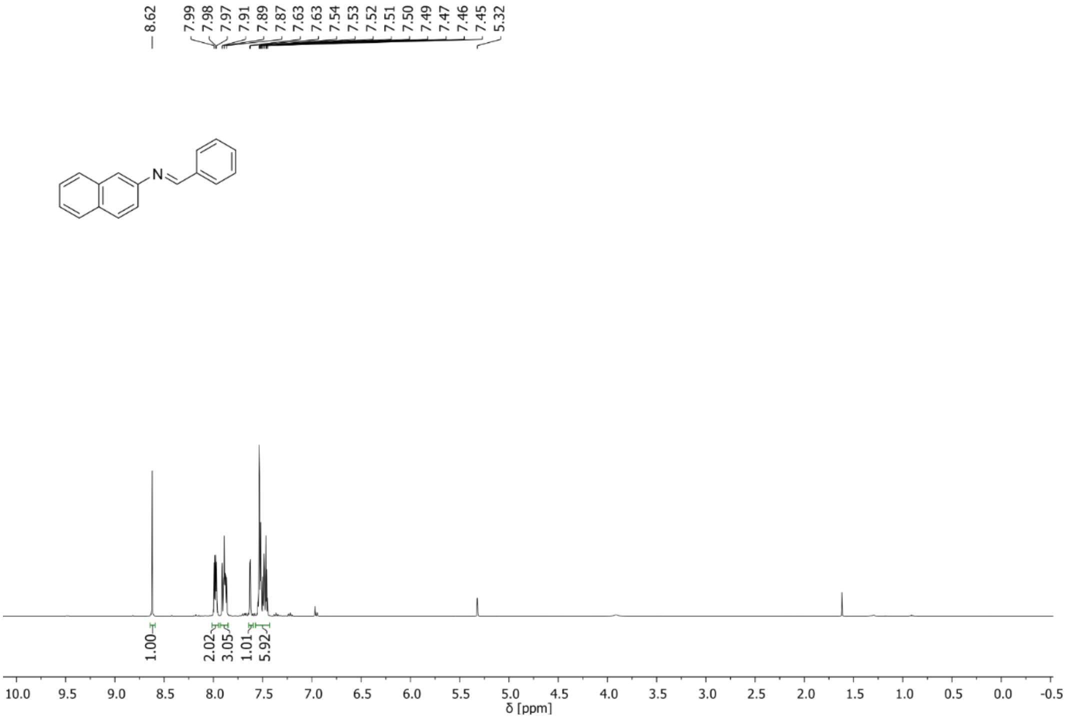

^{1}H NMR: (E)-N-benzylidene-2-naphthylamine (**1m**)

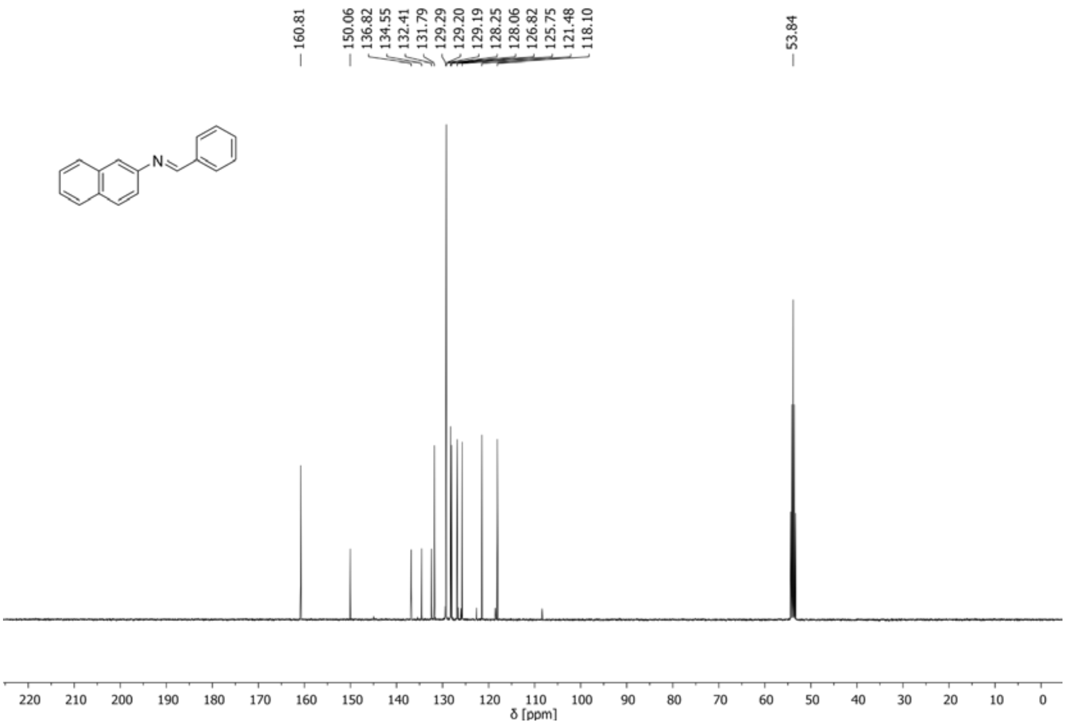

^{13}C{^{1}H} NMR: (E)-N-benzylidene-2-naphthylamine (**1m**)

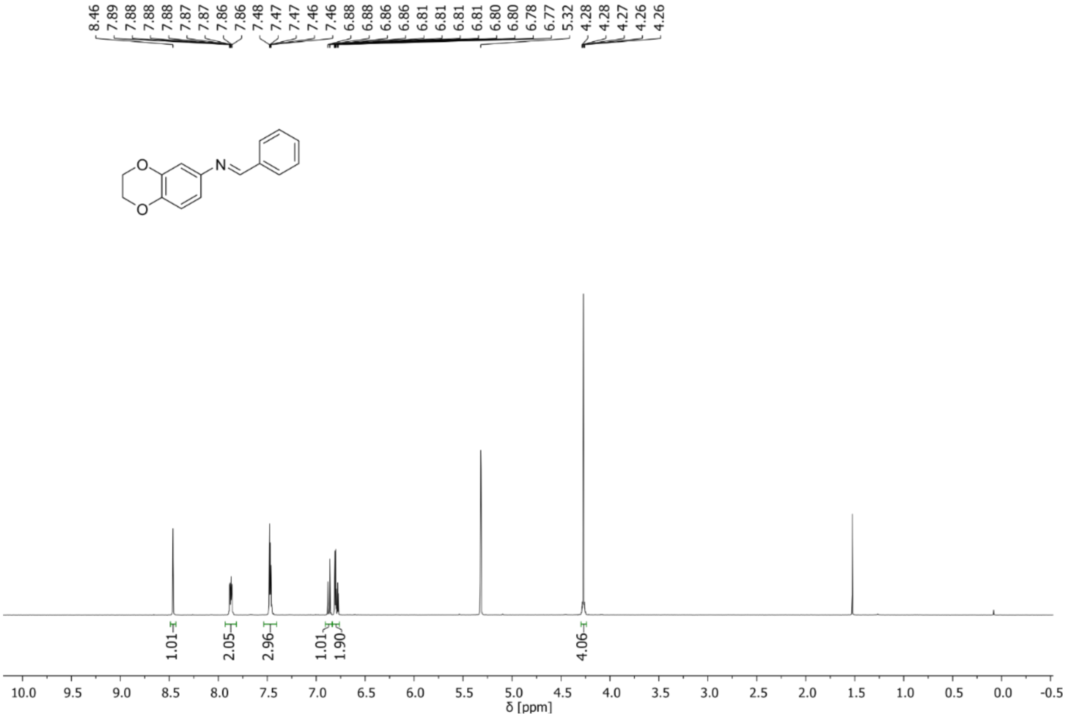

^{1}H NMR: (E)-N-benzylidene-2,3-dihydrobenzo[b][1,4]dioxin-6-amine (**1n**)

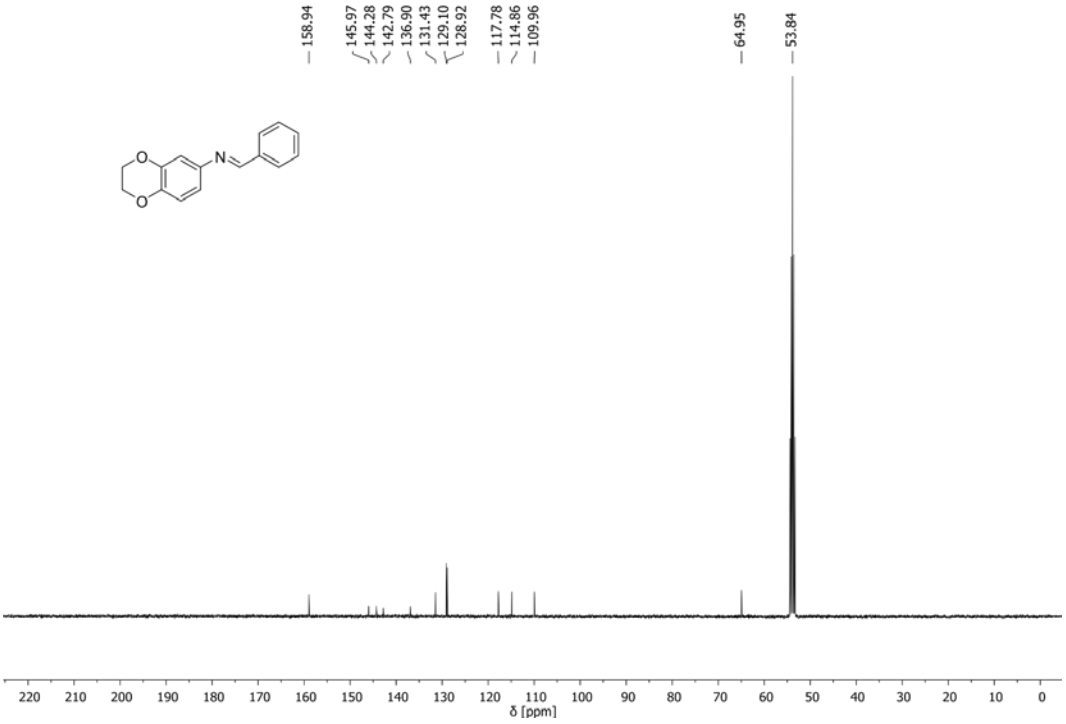

$^{13}C\{^{1}H\}$ NMR: (E)-N-benzylidene-2,3-dihydrobenzo[b][1,4]dioxin-6-amine (**1n**)

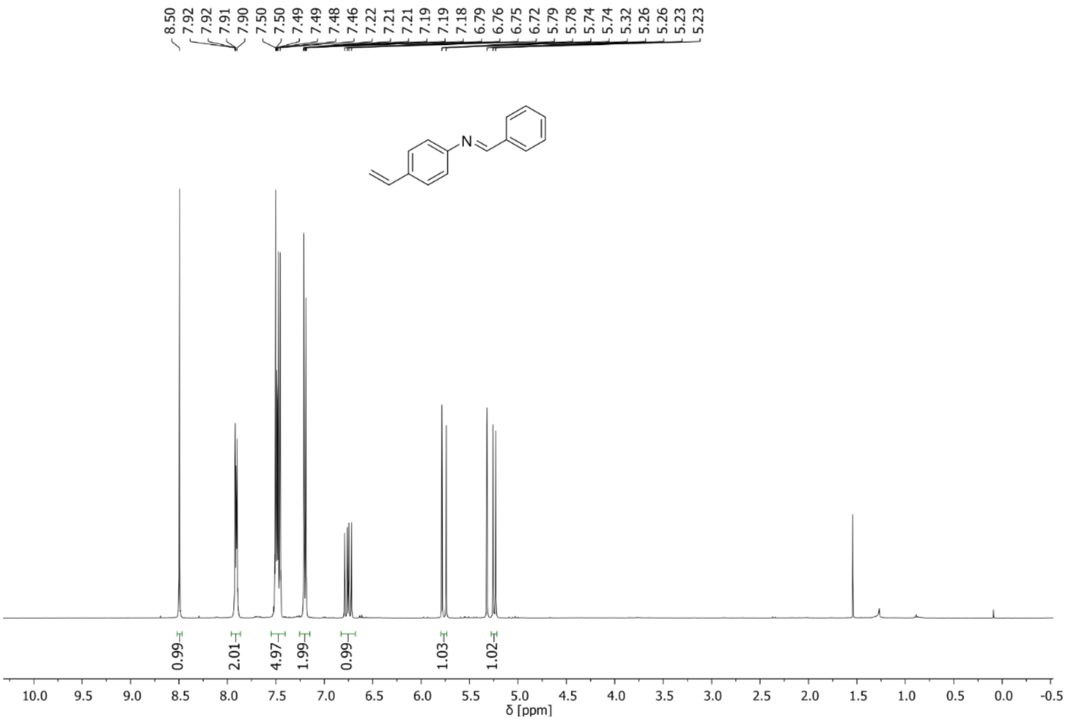

^{1}H NMR: (E)-N-benzylidene-4-vinylaniline (**1o**)

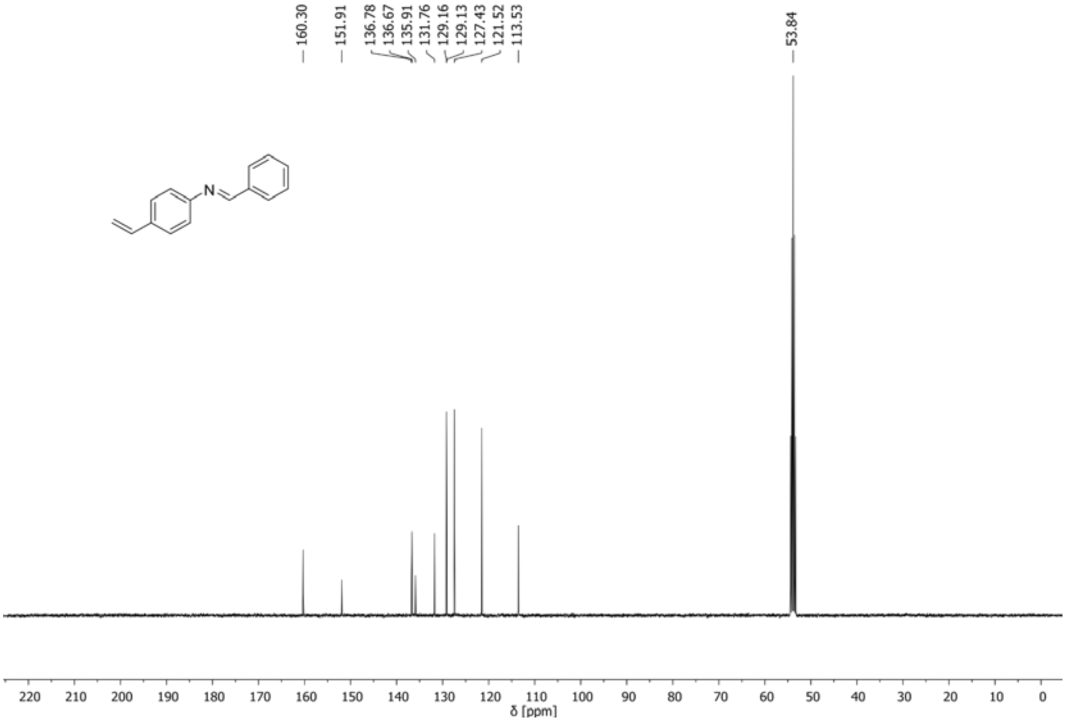

^{13}C{^{1}H} NMR: (E)-N-benzylidene-4-vinylaniline (**1o**)

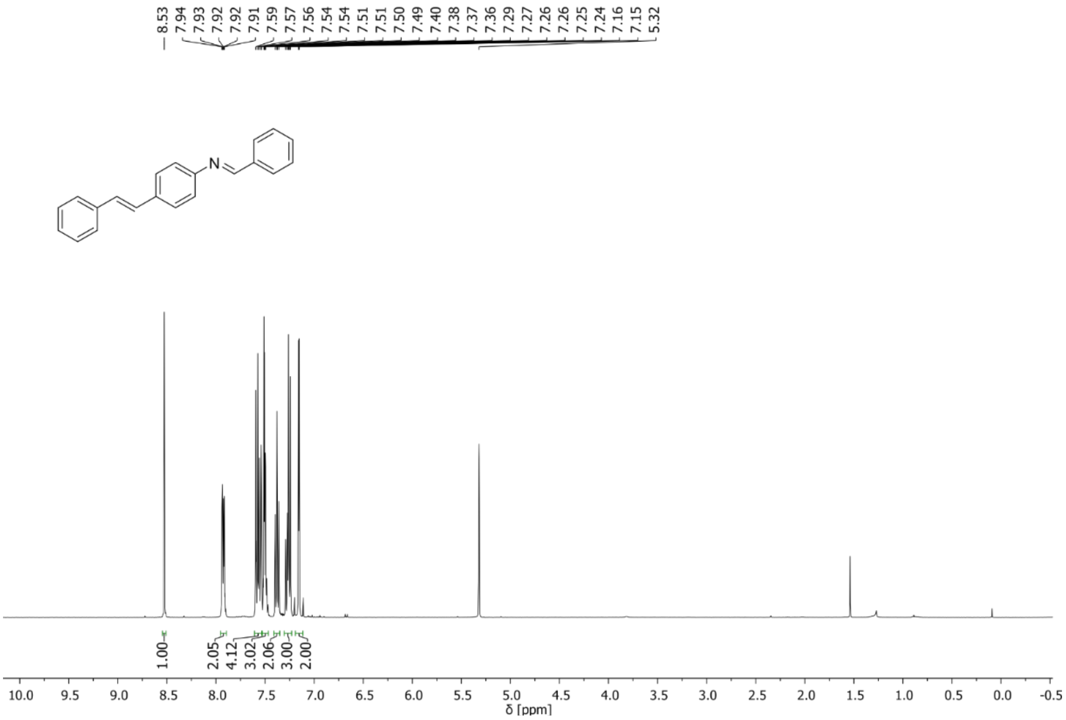

^{1}H NMR: (E)-N-benzylidene-4-aminostilbene (**1p**)

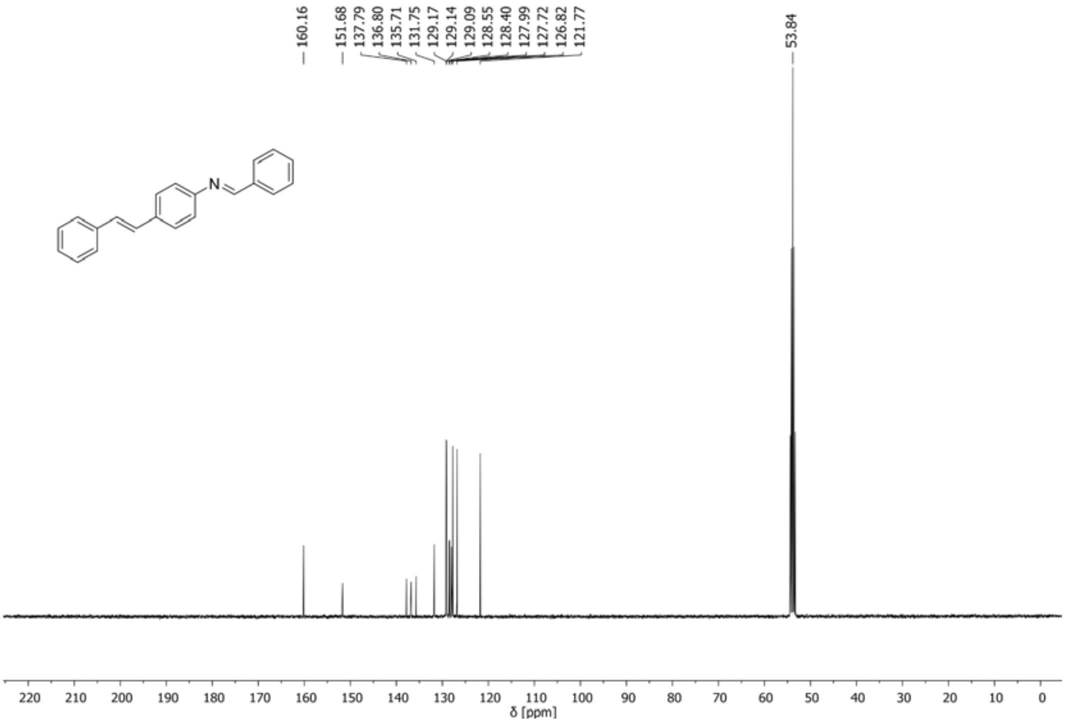

^{13}C{^{1}H} NMR: (E)-N-benzylidene-4-aminostilbene (**1p**)

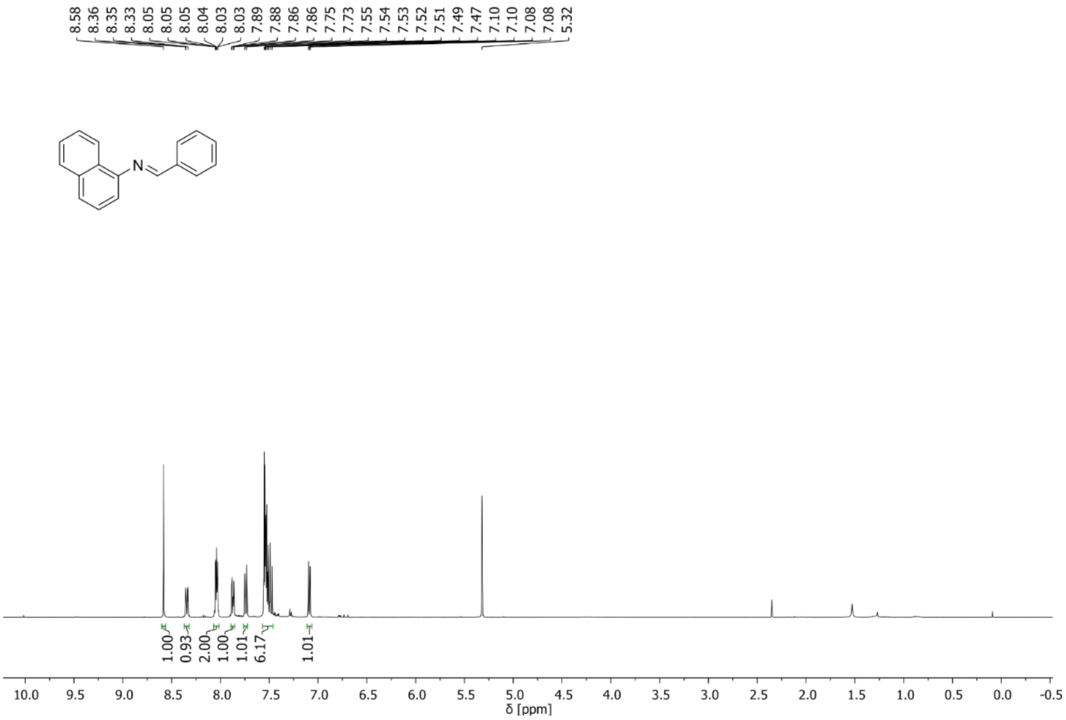

^{1}H NMR: (E)-N-benzylidene-4-naphthylamine (**1q**)

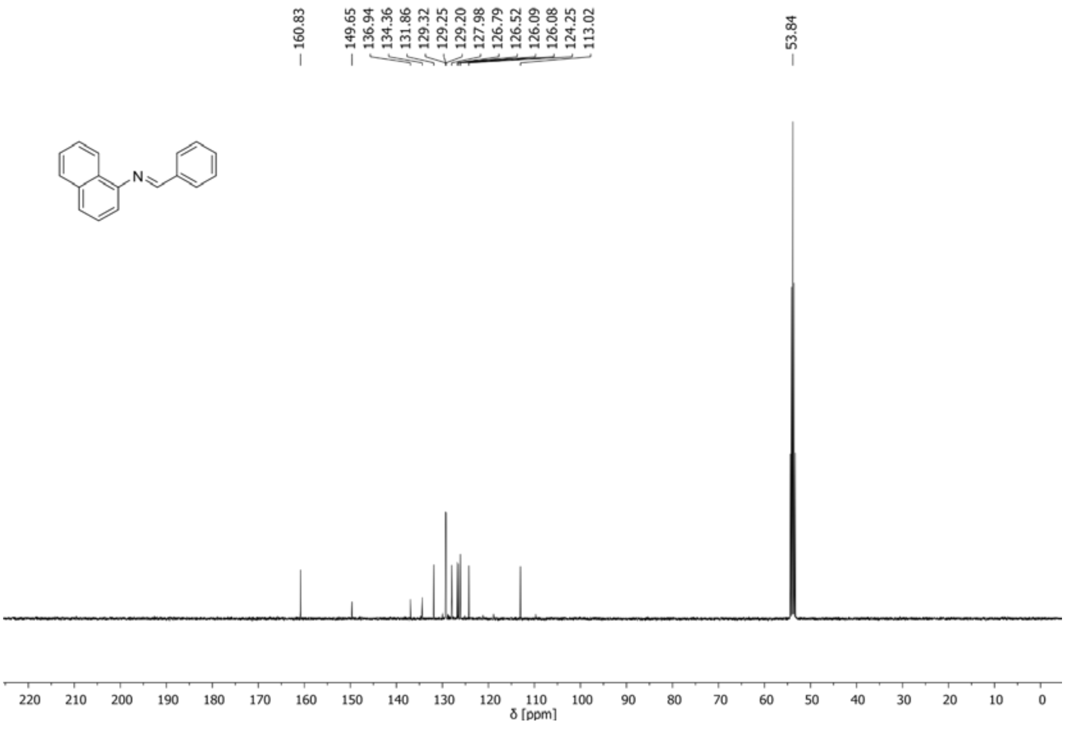

^{13}C{^{1}H} NMR: (E)-N-benzylidene-4-naphthylamine (**1q**)

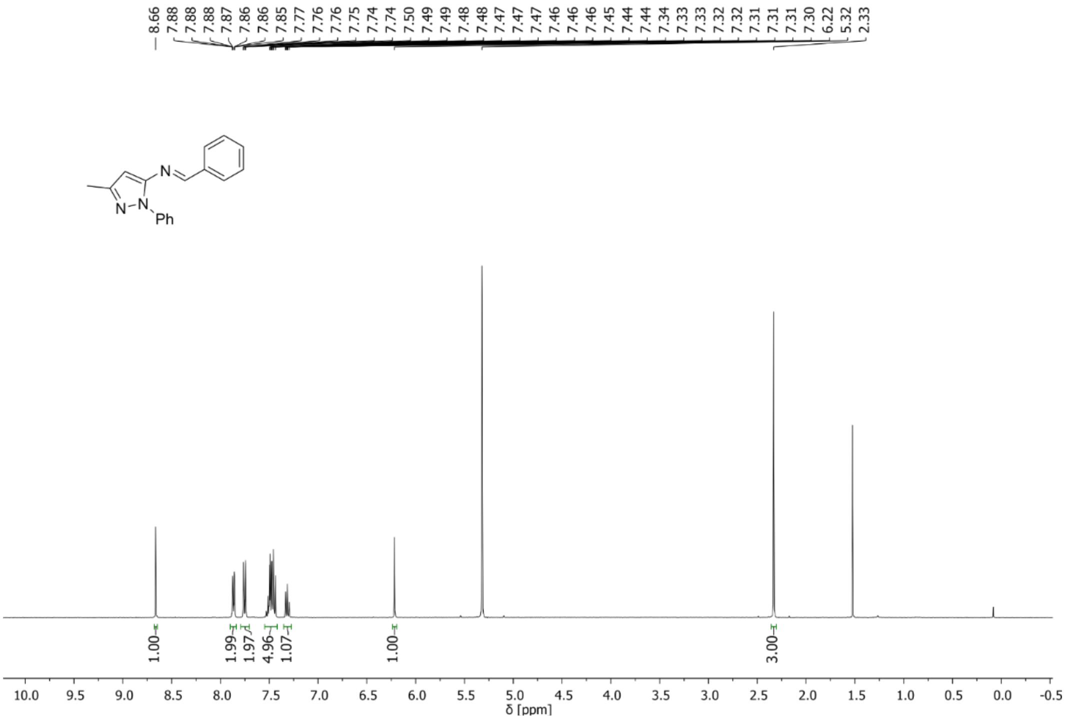

^{1}H NMR: (E)-N-benzylidene-3-methyl-1-phenyl-1H-pyrazol-5-amine (**1r**)

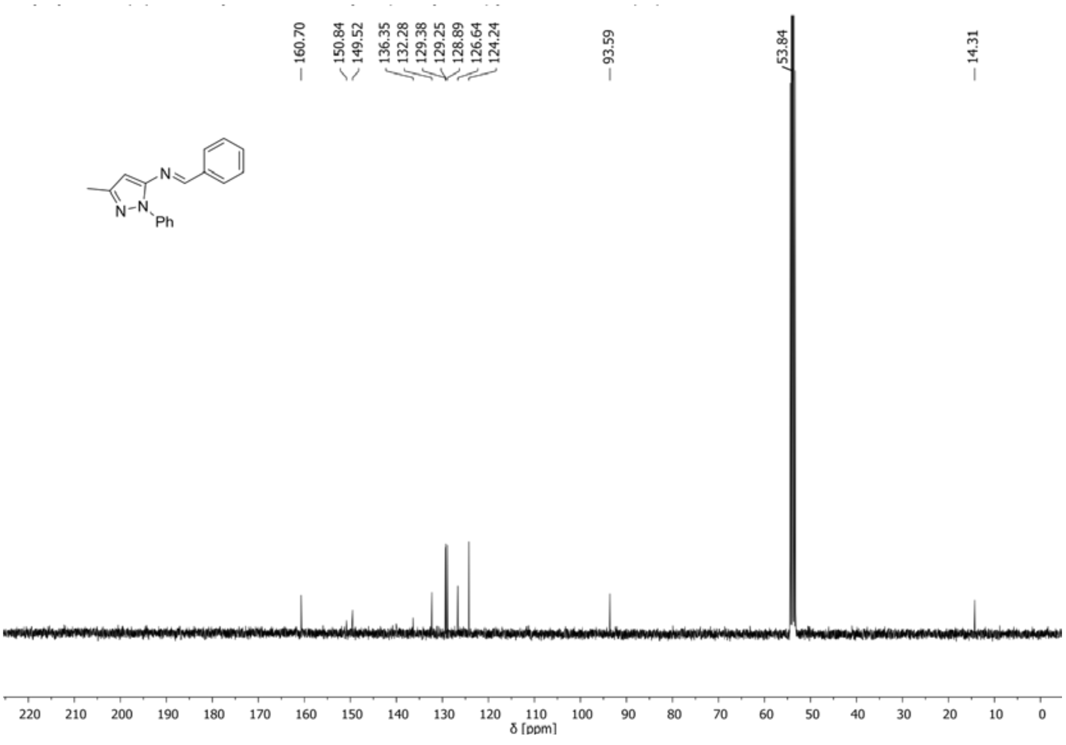

^{13}C{^{1}H} NMR: (E)-N-benzylidene-3-methyl-1-phenyl-1H-pyrazol-5-amine (**1r**)

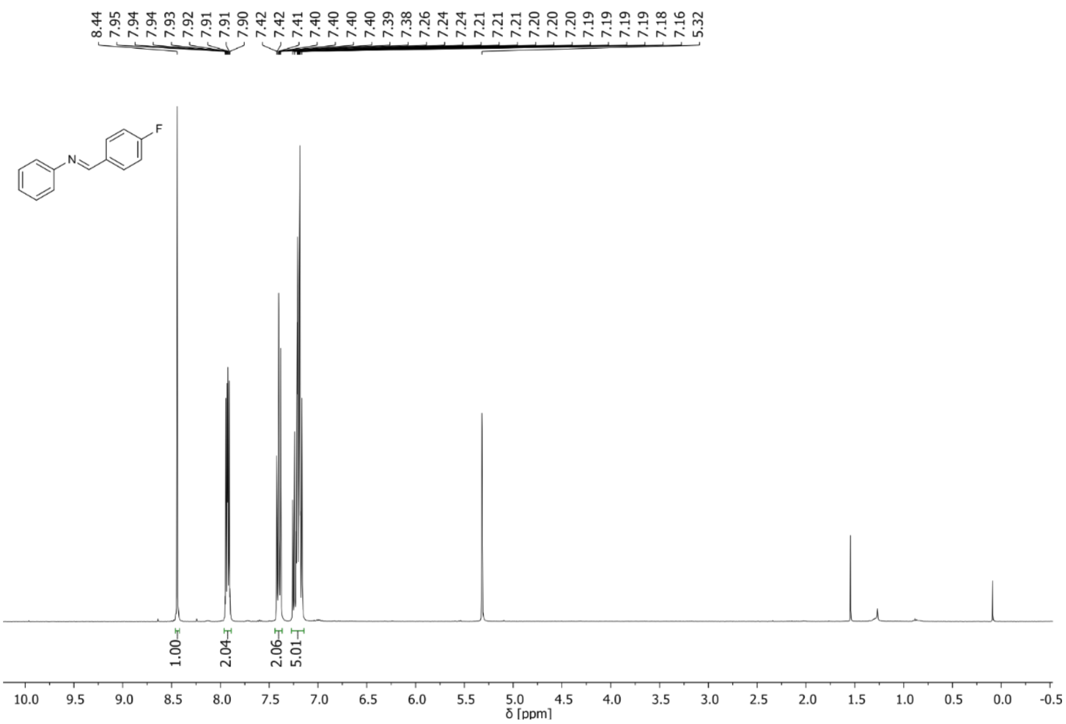

^{1}H NMR: (E)-N-(4-fluorobenzylidene)aniline (**1s**)

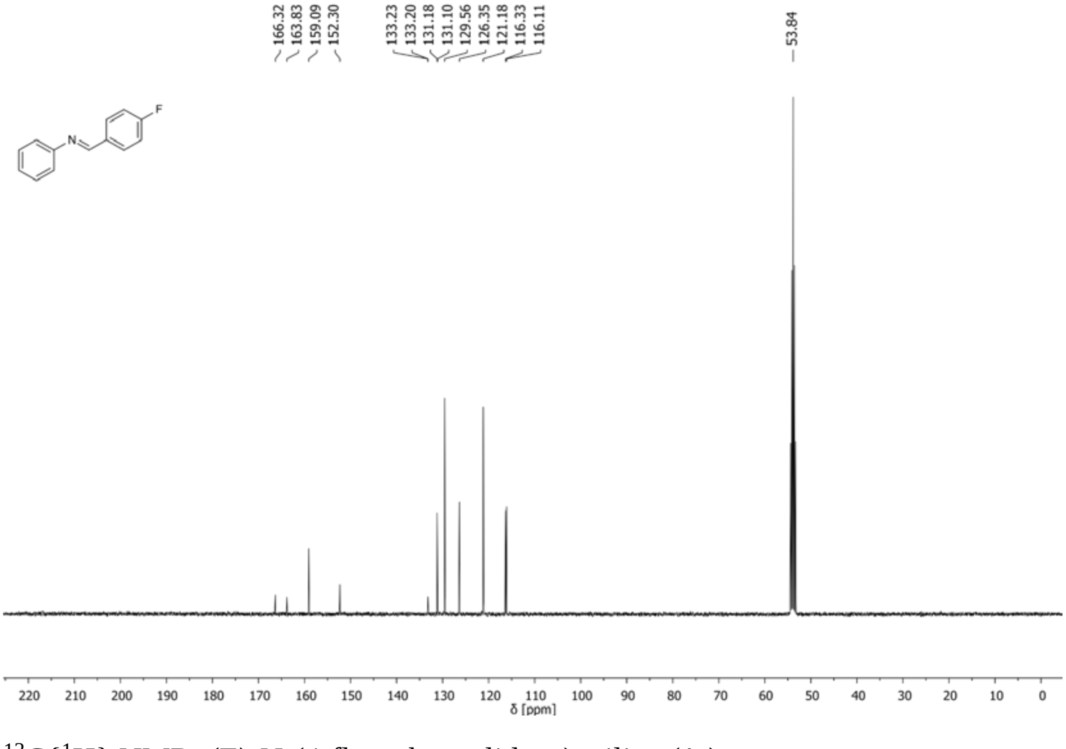

^{13}C{^{1}H} NMR: (E)-N-(4-fluorobenzylidene)aniline (**1s**)

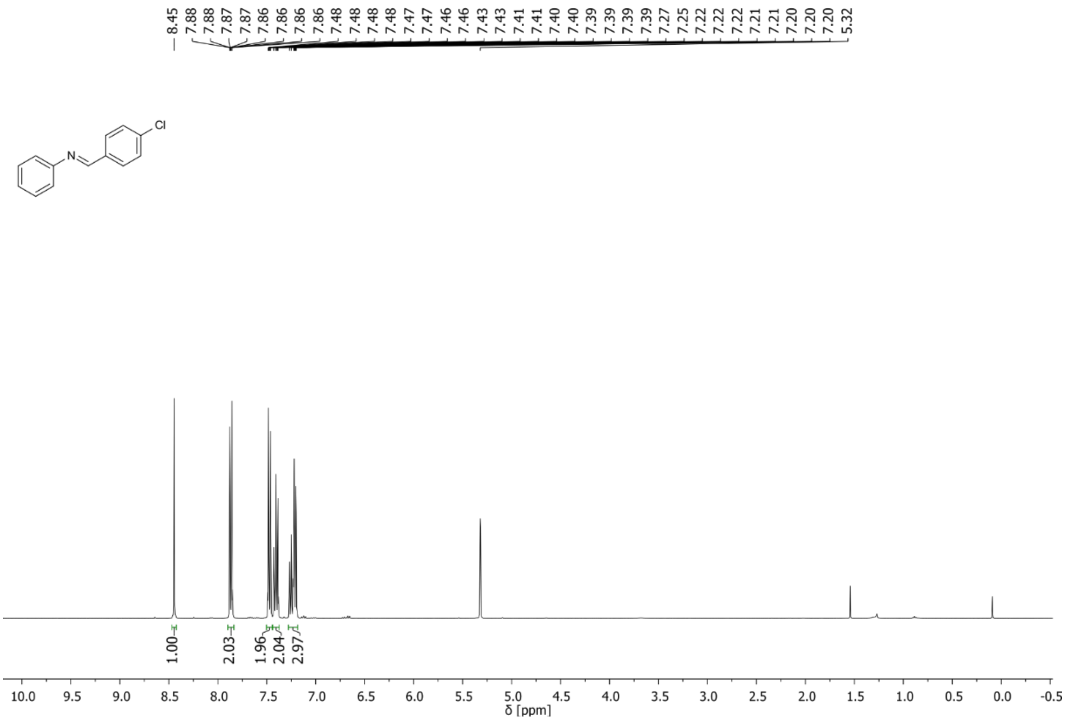

^{1}H NMR: (E)-N-(4-chlorobenzylidene)aniline (**1t**)

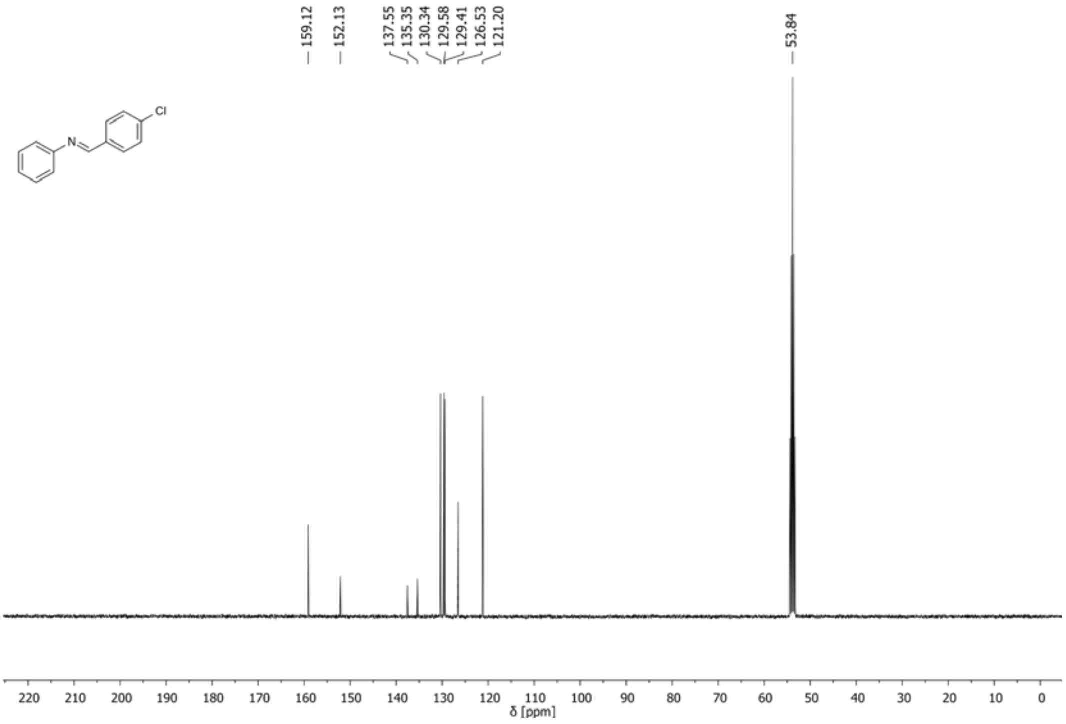

^{13}C{^{1}H} NMR: (E)-N-(4-chlorobenzylidene)aniline (**1t**)

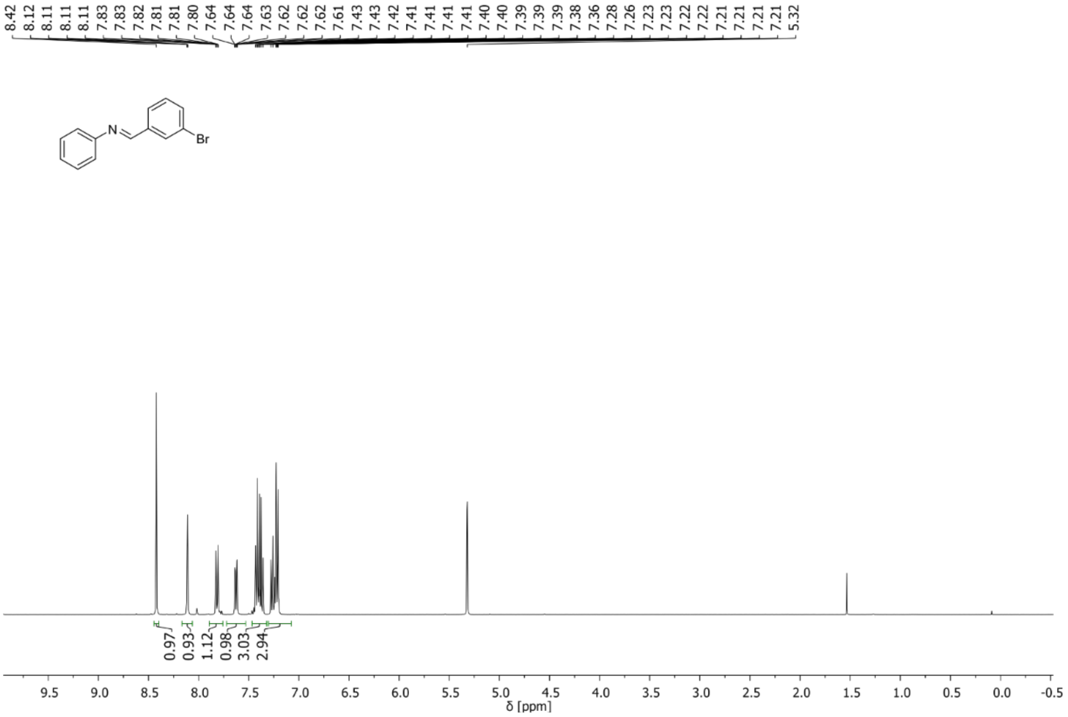

^{1}H NMR: (E)-N-(3-bromobenzylidene)aniline (**1u**)

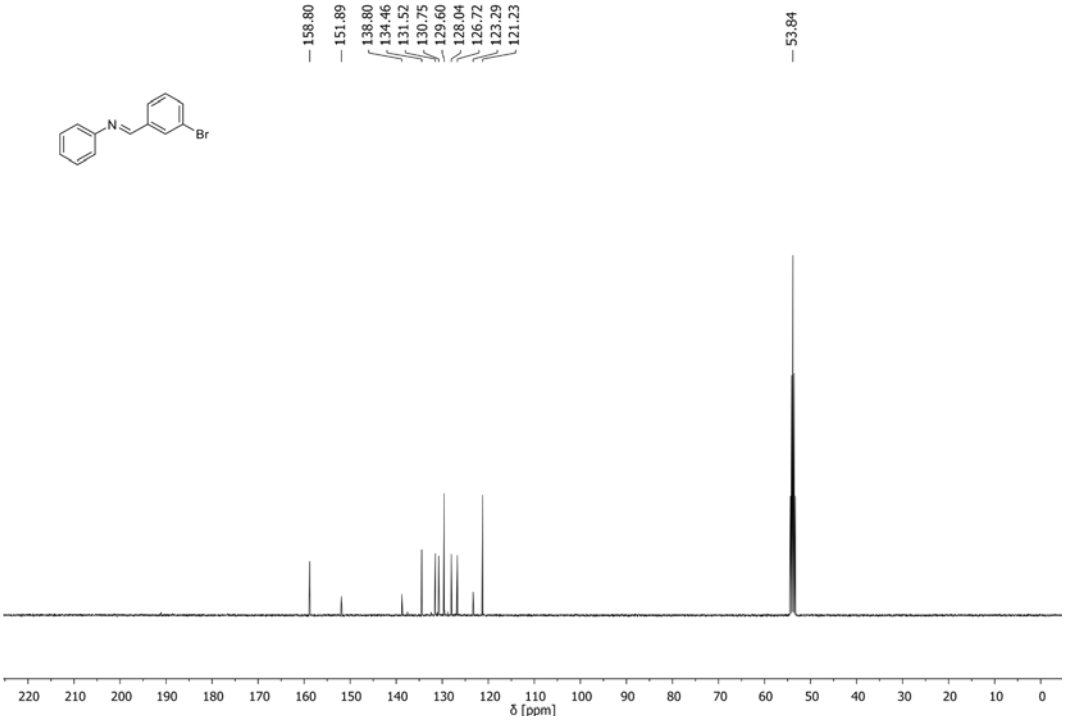

^{13}C{^{1}H} NMR: (E)-N-(3-bromobenzylidene)aniline (**1u**)

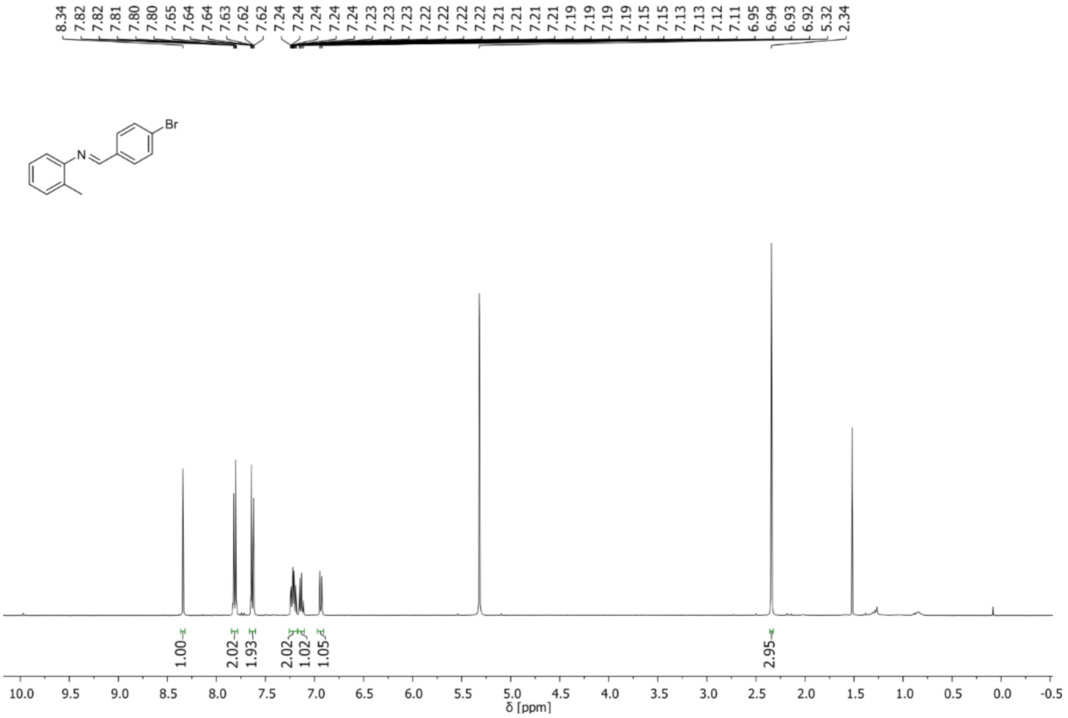

^{1}H NMR: (E)-1-(4-bromophenyl)-N-(o-tolyl)methanimine (**1v**)

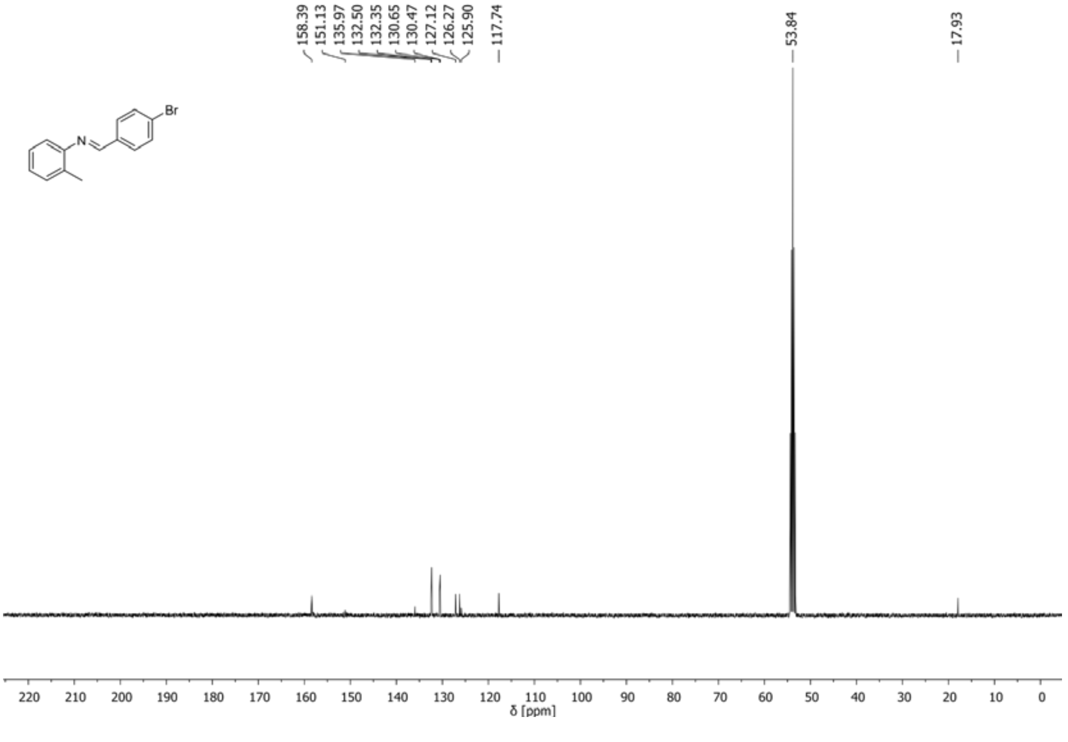

^{13}C{^{1}H} NMR: (E)-1-(4-bromophenyl)-N-(o-tolyl)methanimine (**1v**)

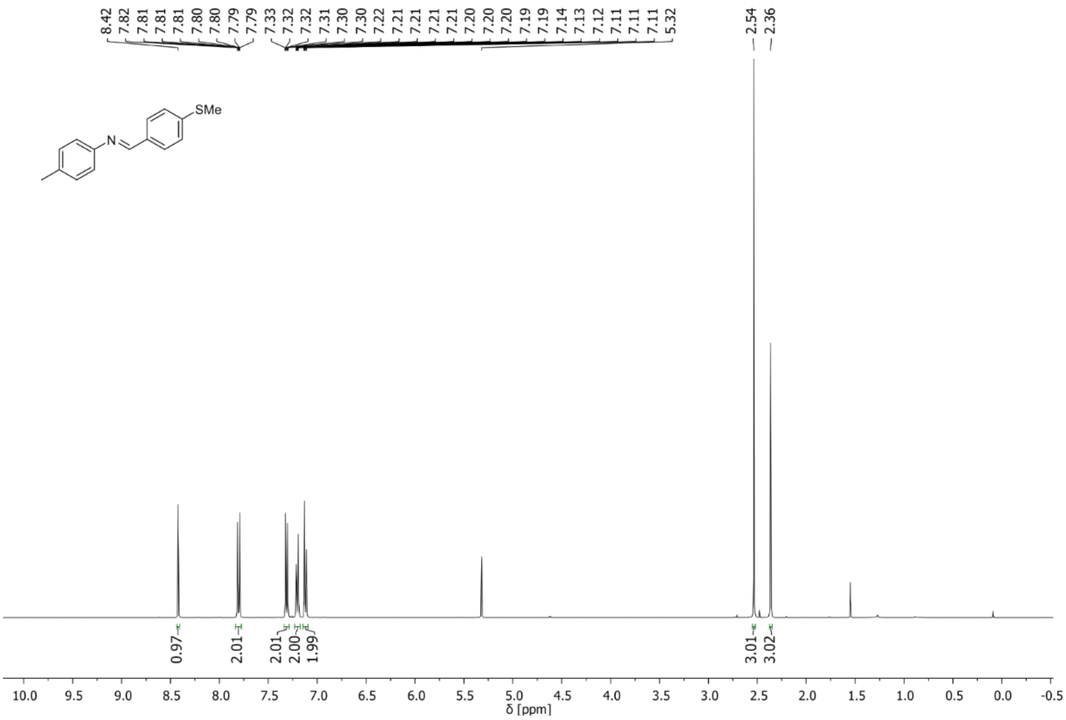

^{1}H NMR: (E)-1-(4-(methylthio)phenyl)-N-(p-tolyl)methanimine (**1w**)

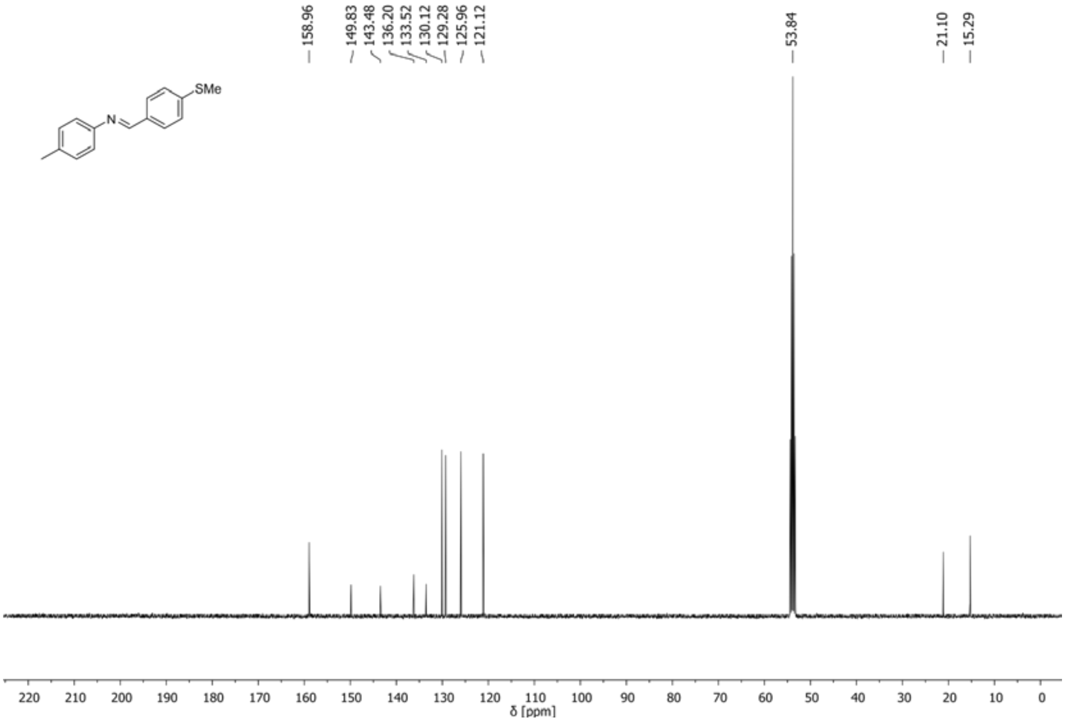

^{13}C{^{1}H} NMR: (E)-1-(4-(methylthio)phenyl)-N-(p-tolyl)methanimine (**1w**)

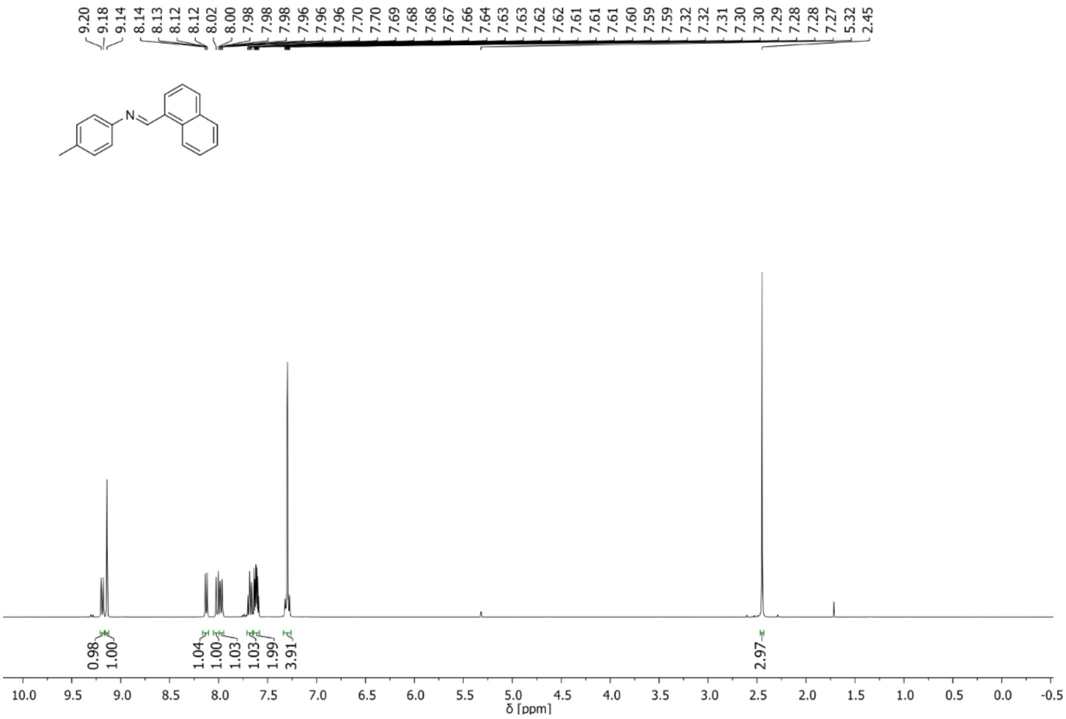

^{1}H NMR: (E)-1-(naphthalen-1-yl)-N-(p-tolyl)methanimine (**1x**)

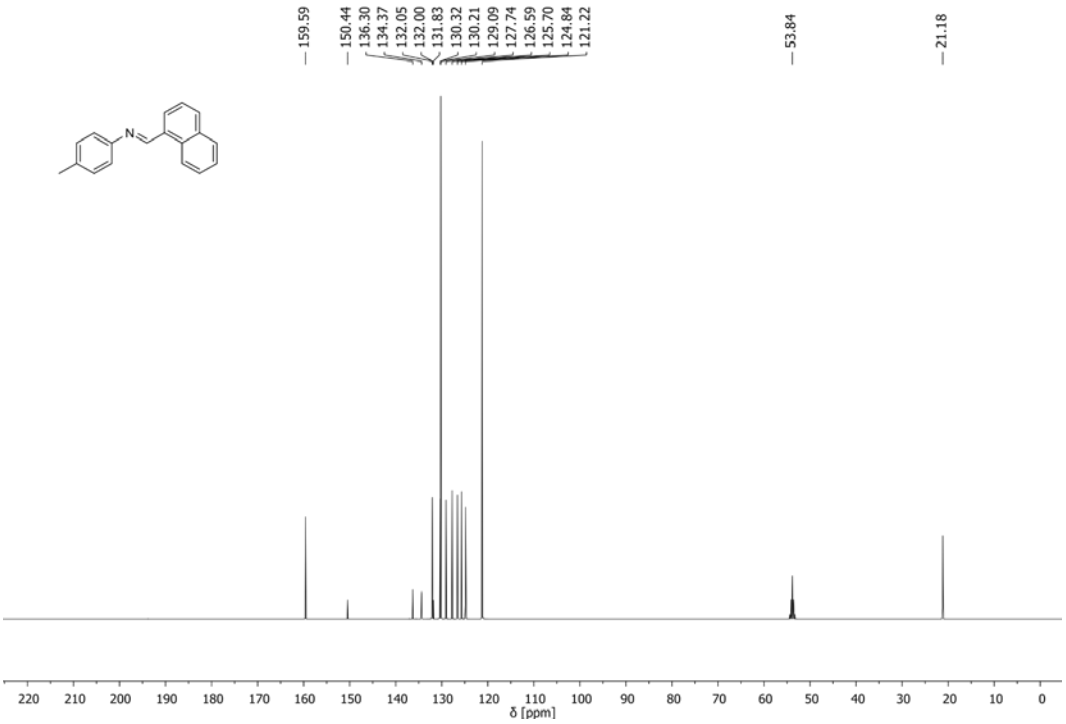

^{13}C{^{1}H} NMR: (E)-1-(naphthalen-1-yl)-N-(p-tolyl)methanimine (**1x**)

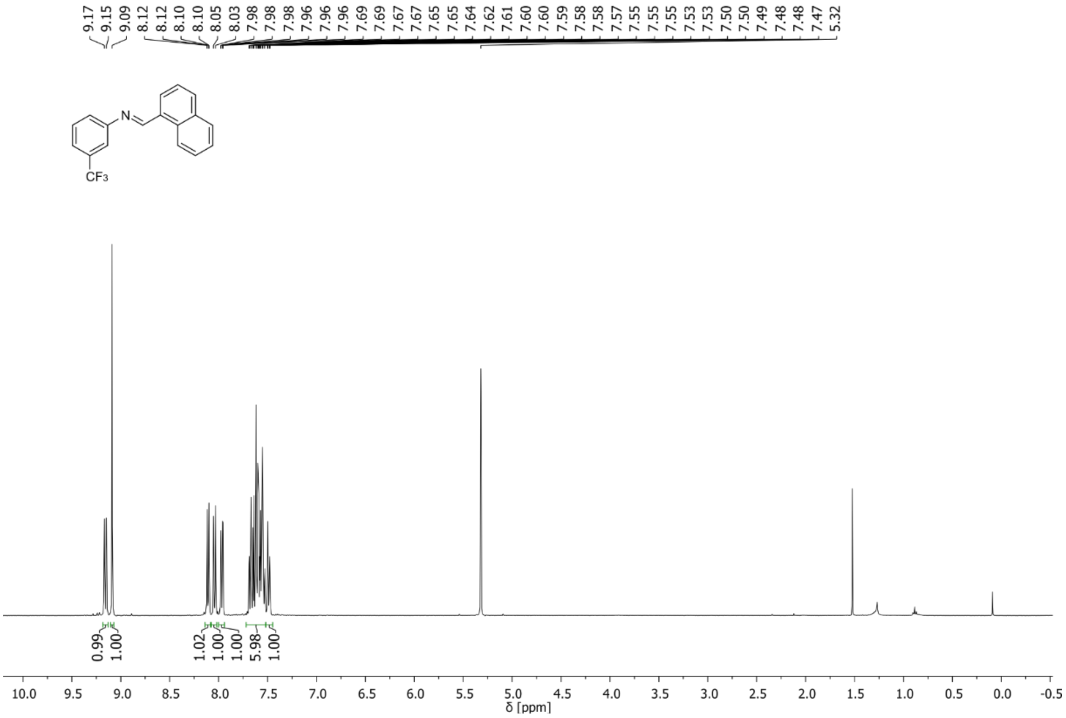

^{1}H NMR: (E)-1-(furan-2-yl)-N-(3-(trifluoromethyl)phenyl)methanimine (**1y**)

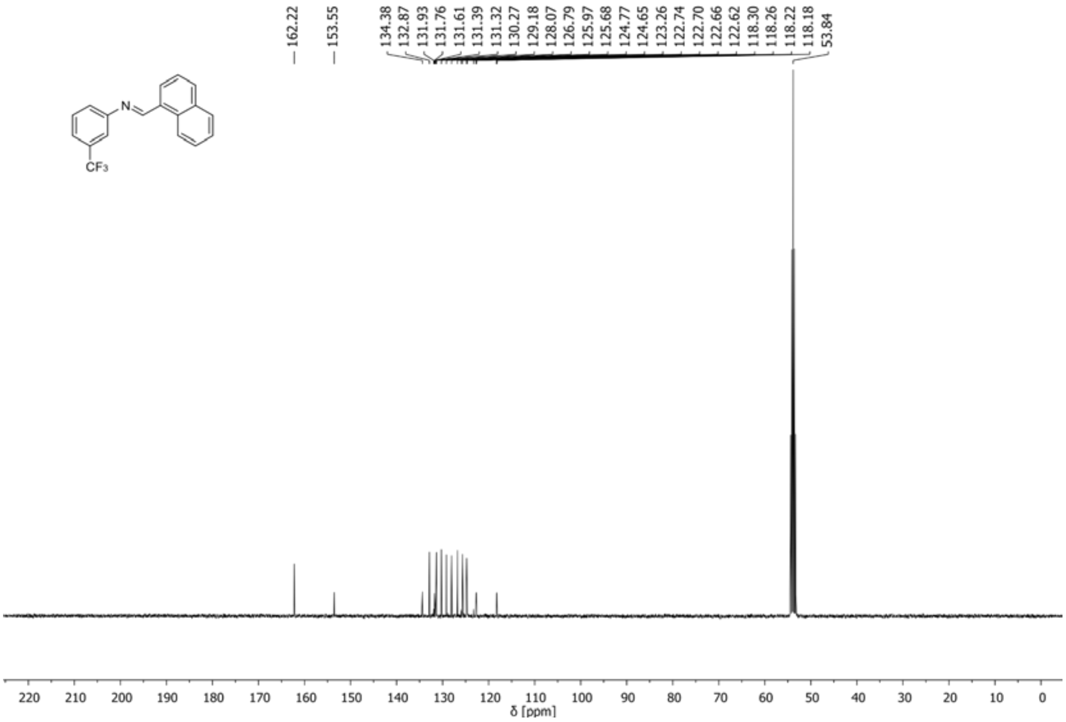

^{13}C{^{1}H} NMR: (E)-1-(furan-2-yl)-N-(3-(trifluoromethyl)phenyl)methanimine (**1y**)

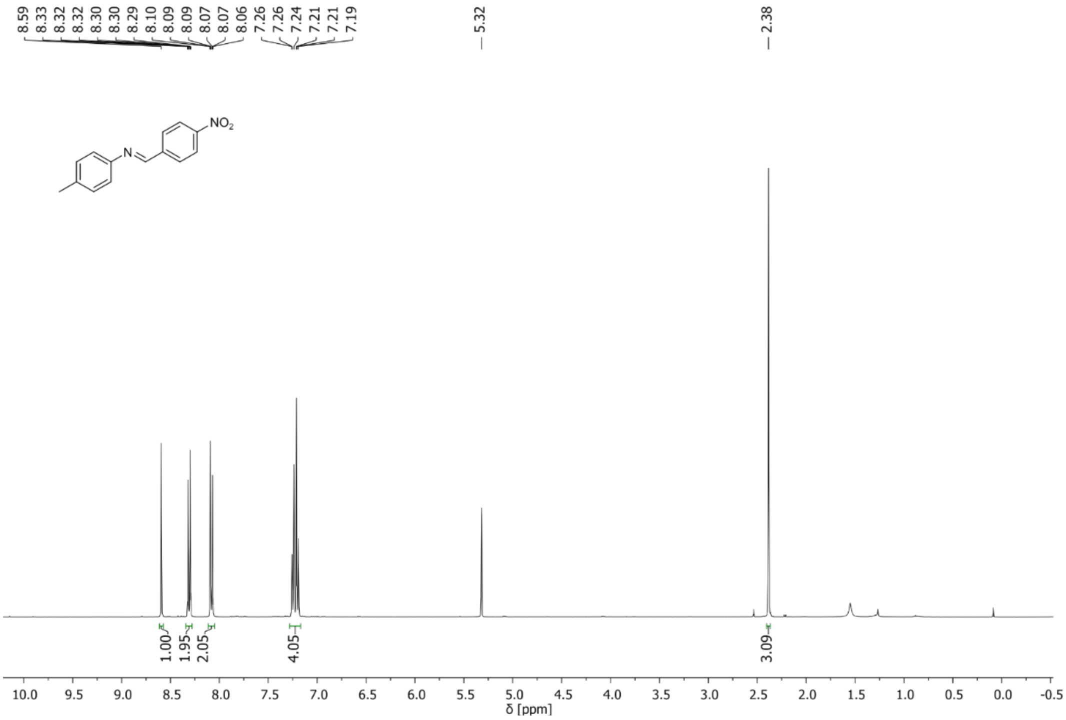

^{1}H NMR: (E)-4-methyl-N-(4-nitrobenzylidene)aniline (**1z**)

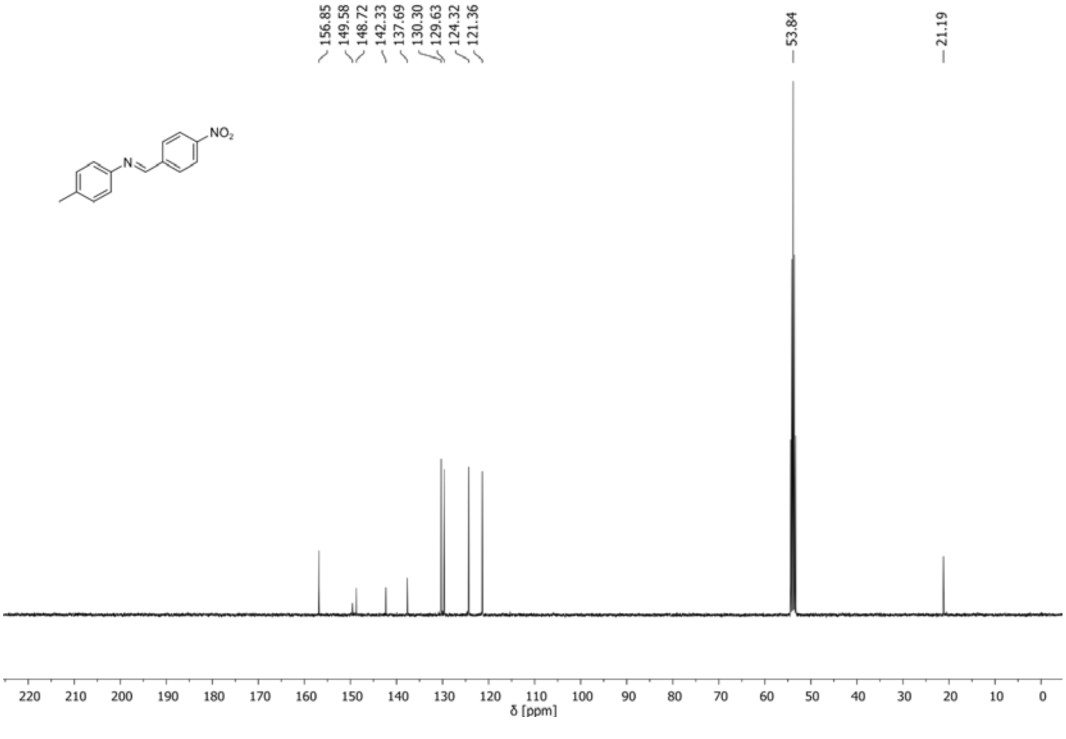

$^{13}C\{^{1}H\}$ NMR: (E)-4-methyl-N-(4-nitrobenzylidene)aniline (**1z**)

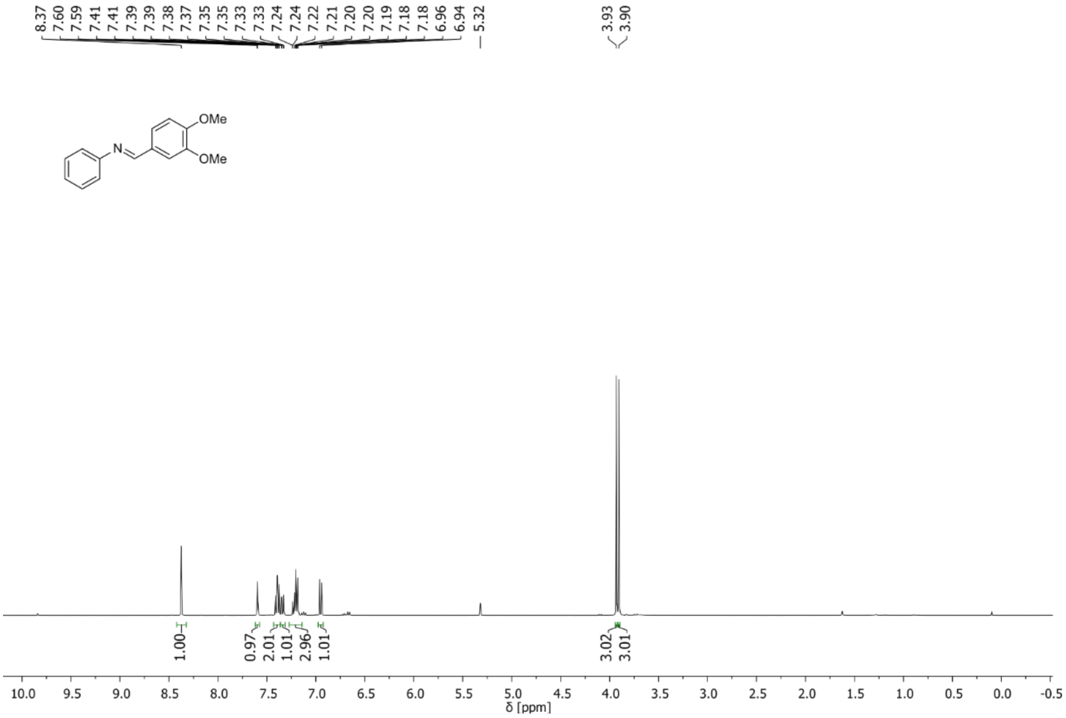

^{1}H NMR: (E)-N-(3,4-Dimethoxybenzylidene)aniline (**1aa**)

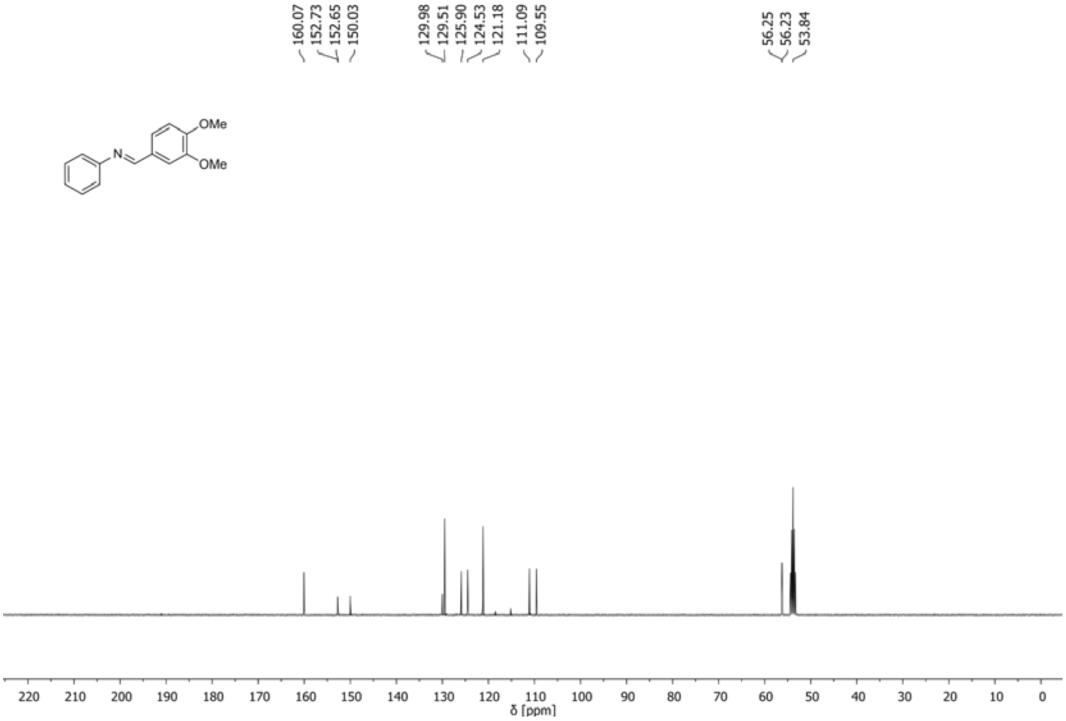

^{13}C{^{1}H} NMR: (E)-N-(3,4-Dimethoxybenzylidene)aniline (**1aa**)

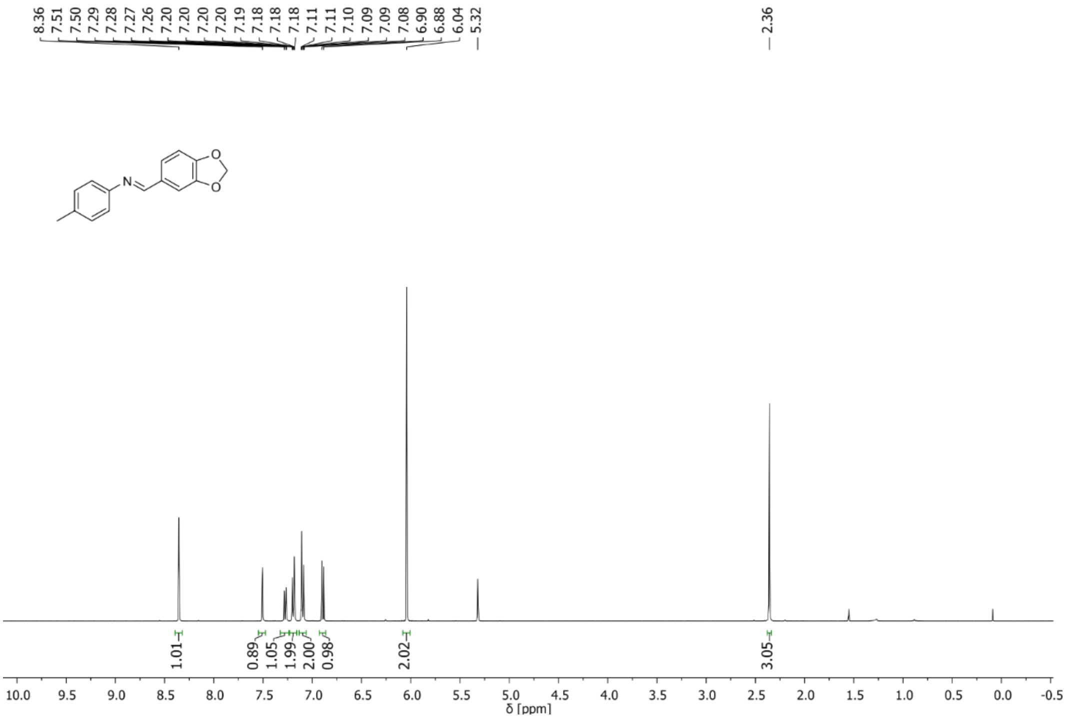

^{1}H NMR: (E)-1-(benzo[d][1,3]dioxol-5-yl)-N-(p-tolyl)methanimine (**1ab**)

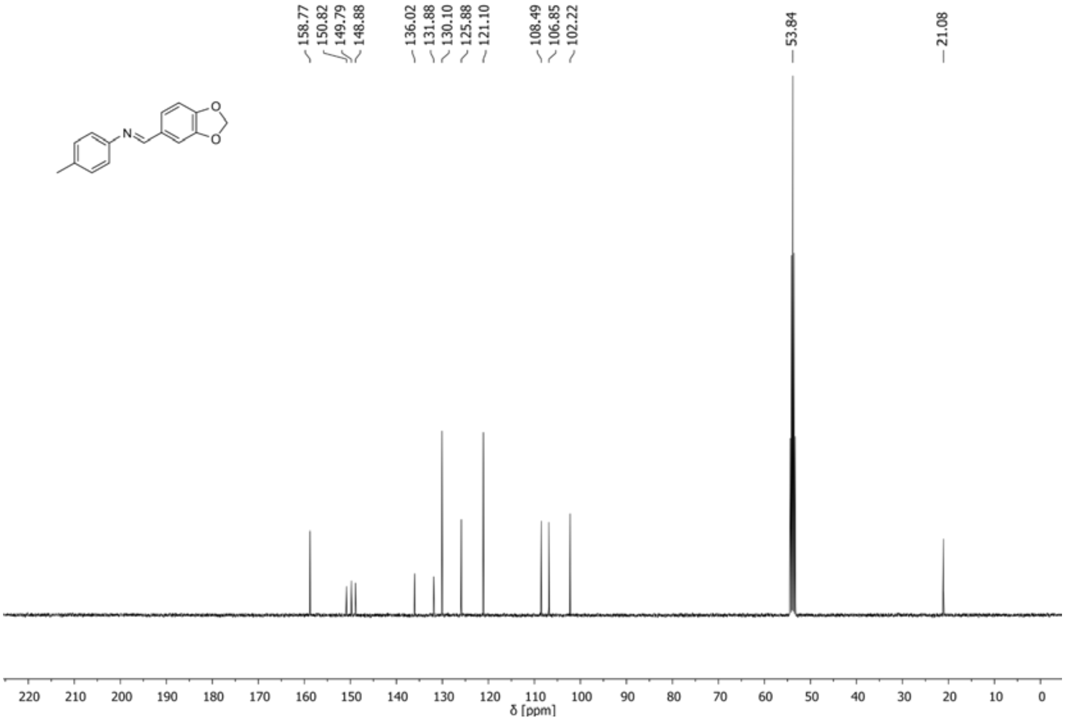

^{13}C{^{1}H} NMR: (E)-1-(benzo[d][1,3]dioxol-5-yl)-N-(p-tolyl)methanimine (**1ab**)

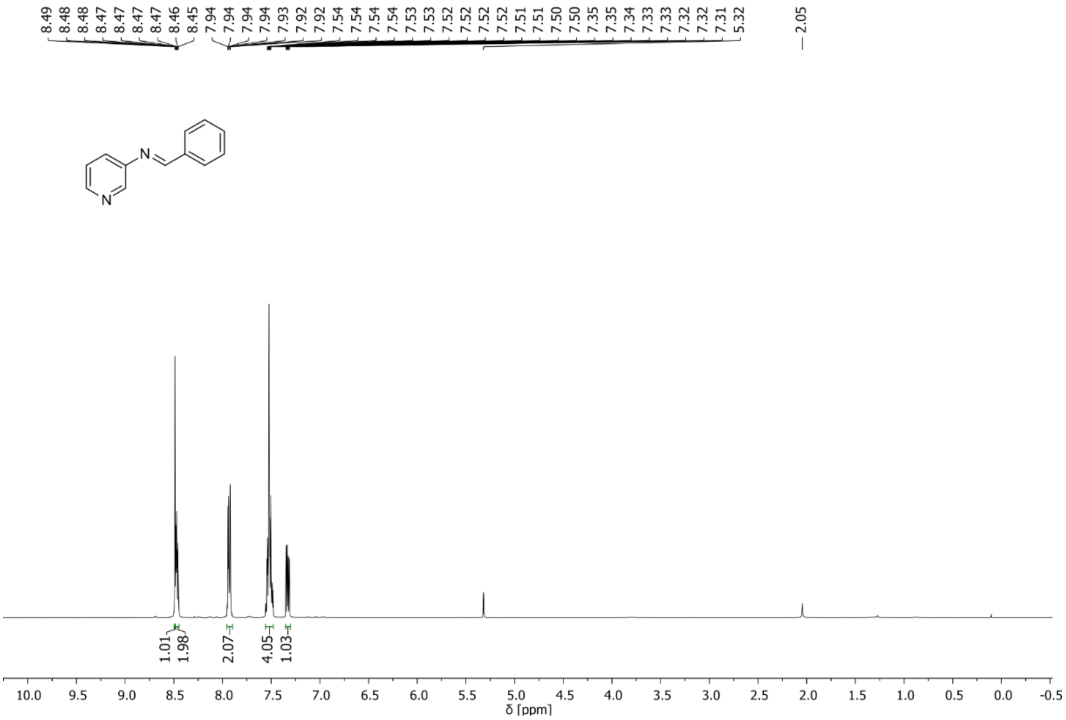

^{1}H NMR: (E)-N-benzylidene-3-pyridinamine (**1ac**)

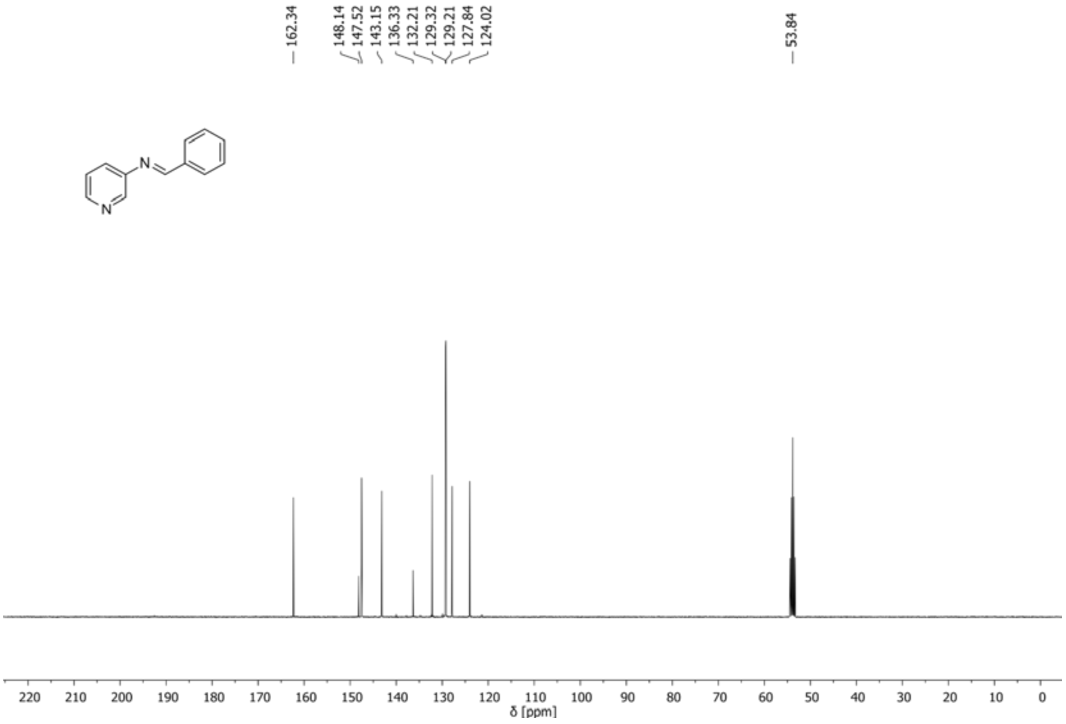

^{13}C{^{1}H} NMR: (E)-N-benzylidene-3-pyridinamine (**1ac**)

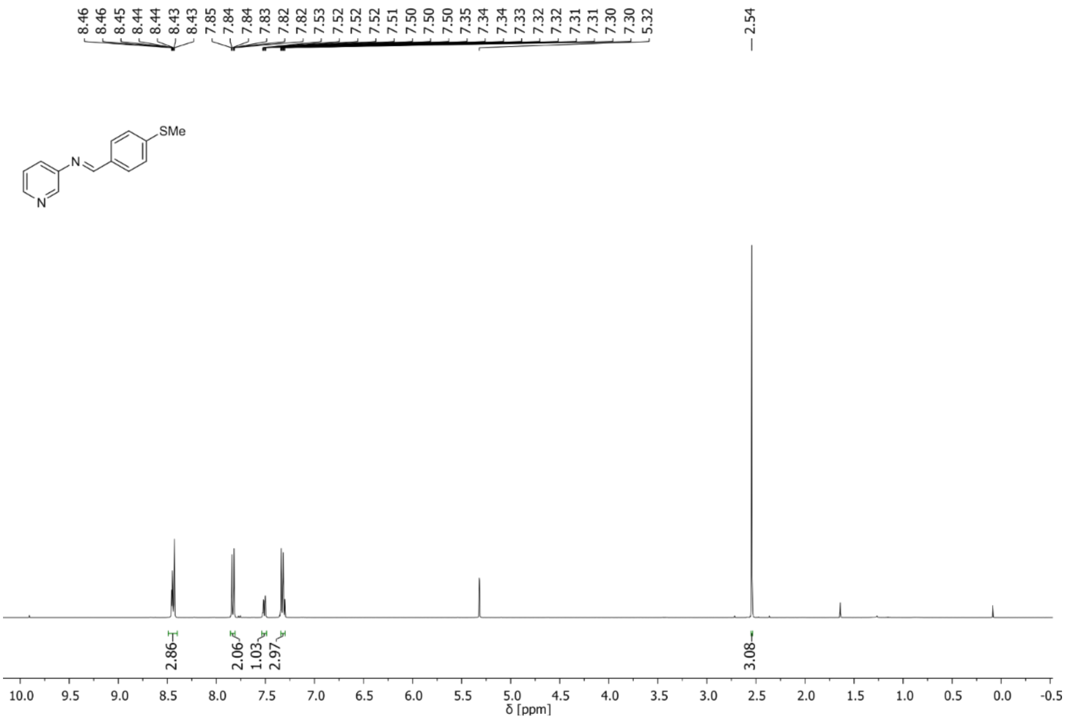

^{1}H NMR: (E)-1-(4-(methylthio)phenyl)-N-(pyridine-3-yl)methanimine (**1ad**)

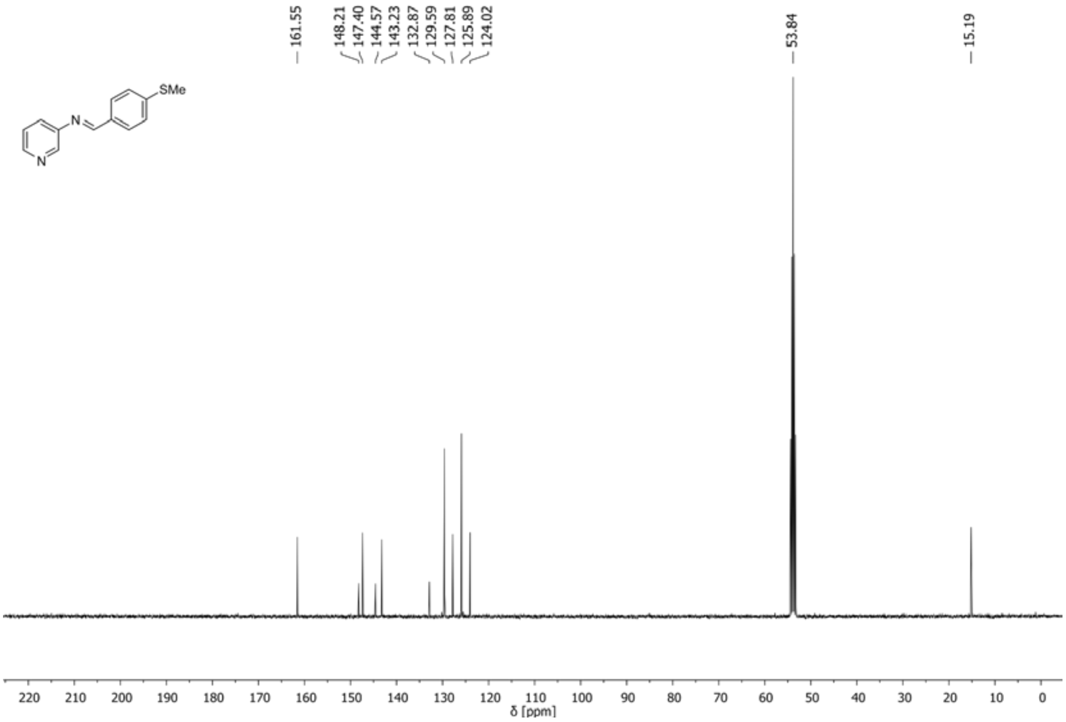

^{13}C{^{1}H} NMR: (E)-1-(4-(methylthio)phenyl)-N-(pyridine-3-yl)methanimine (**1ad**)

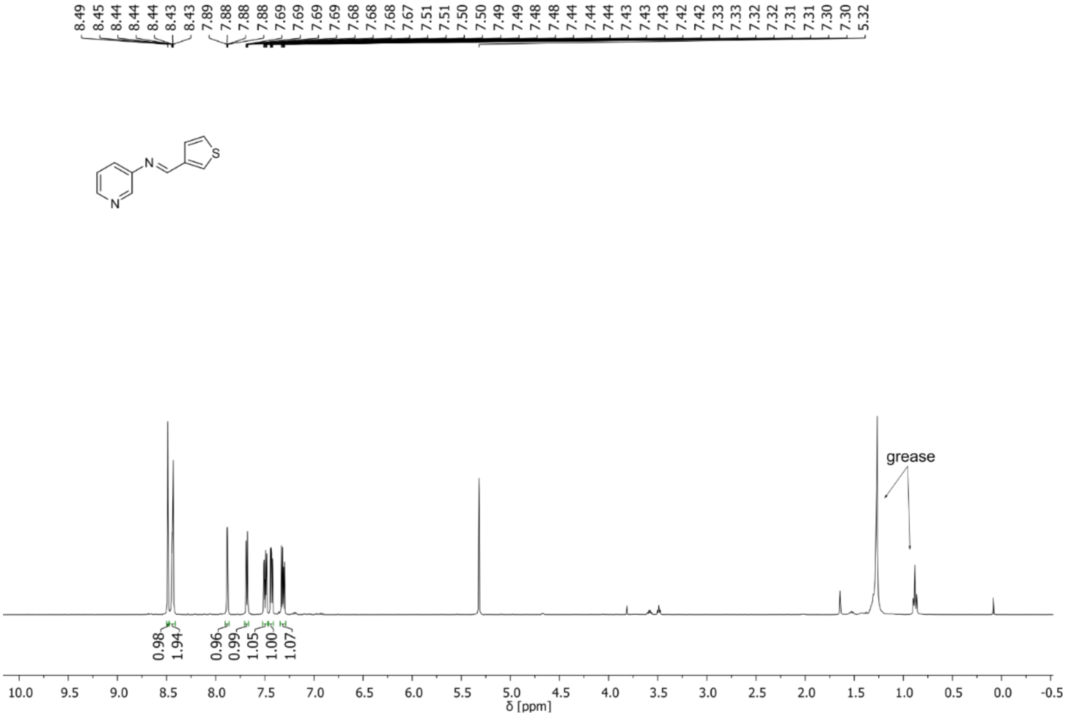

^{1}H NMR: (E)-N-(pyridin-3-yl)-1-(thiophen-3-yl)methanimine (**1ae**)

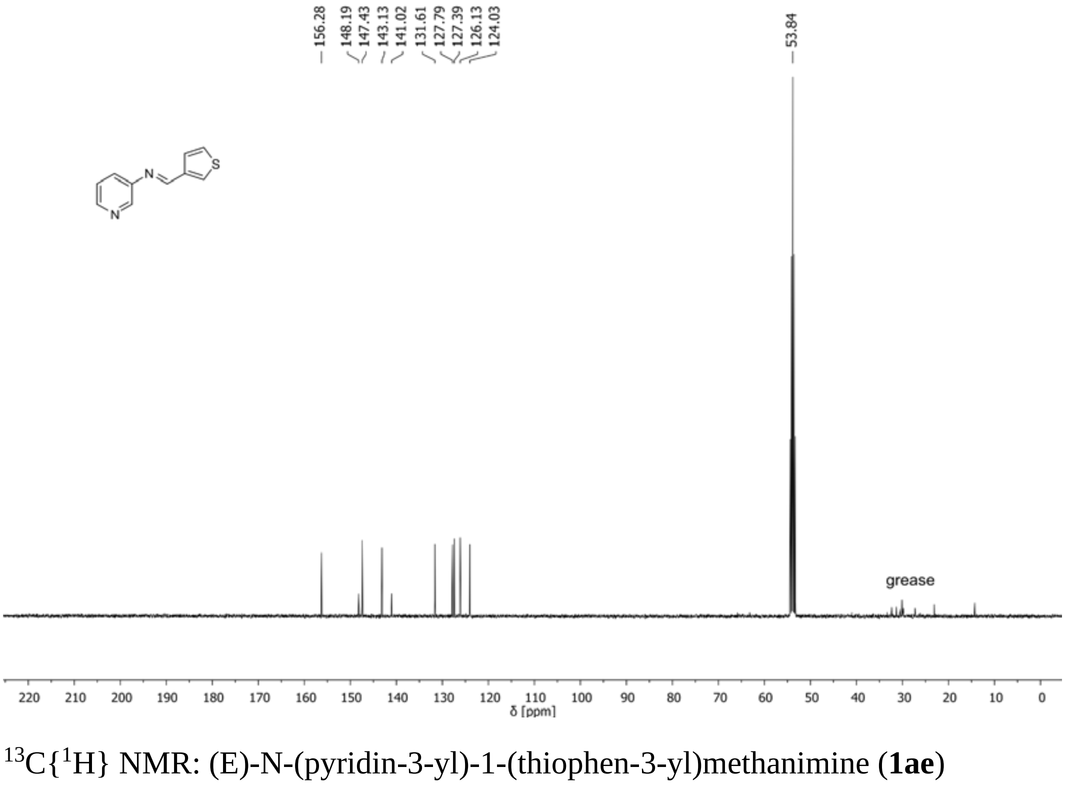

$^{13}C\{^{1}H\}$ NMR: (E)-N-(pyridin-3-yl)-1-(thiophen-3-yl)methanimine (**1ae**)

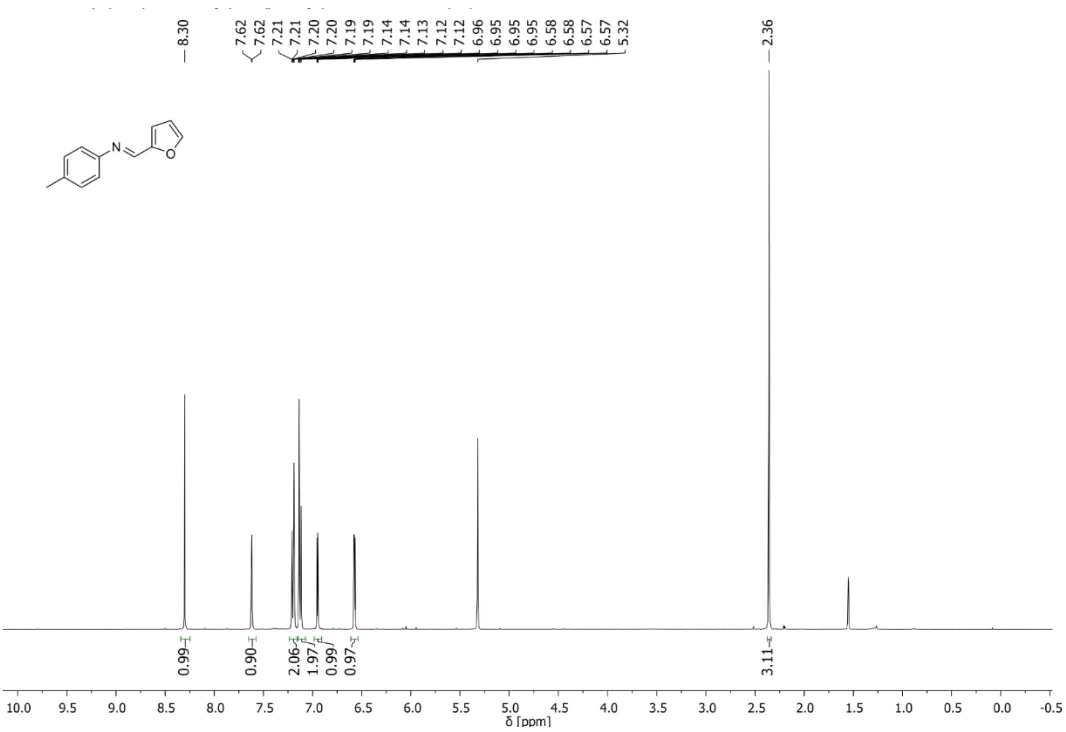

^{1}H NMR: (E)-1-(furan-2-yl)-N-(p-tolyl)methanimine (**1af**)

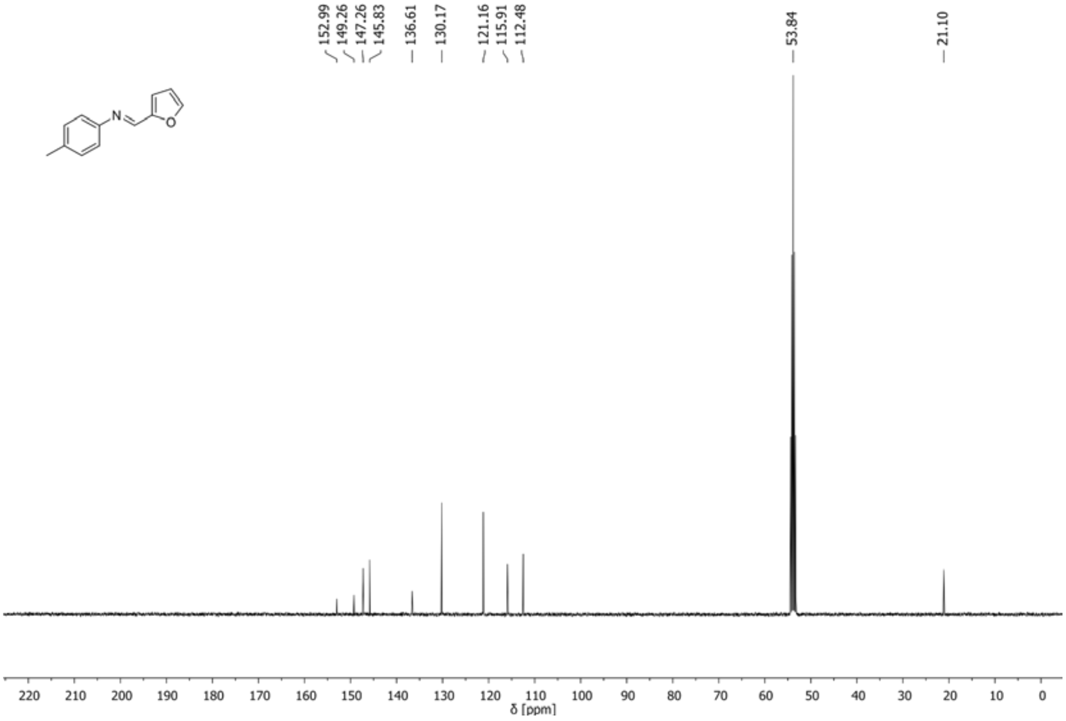

$^{13}C\{^{1}H\}$ NMR: (E)-1-(furan-2-yl)-N-(p-tolyl)methanimine (**1af**)

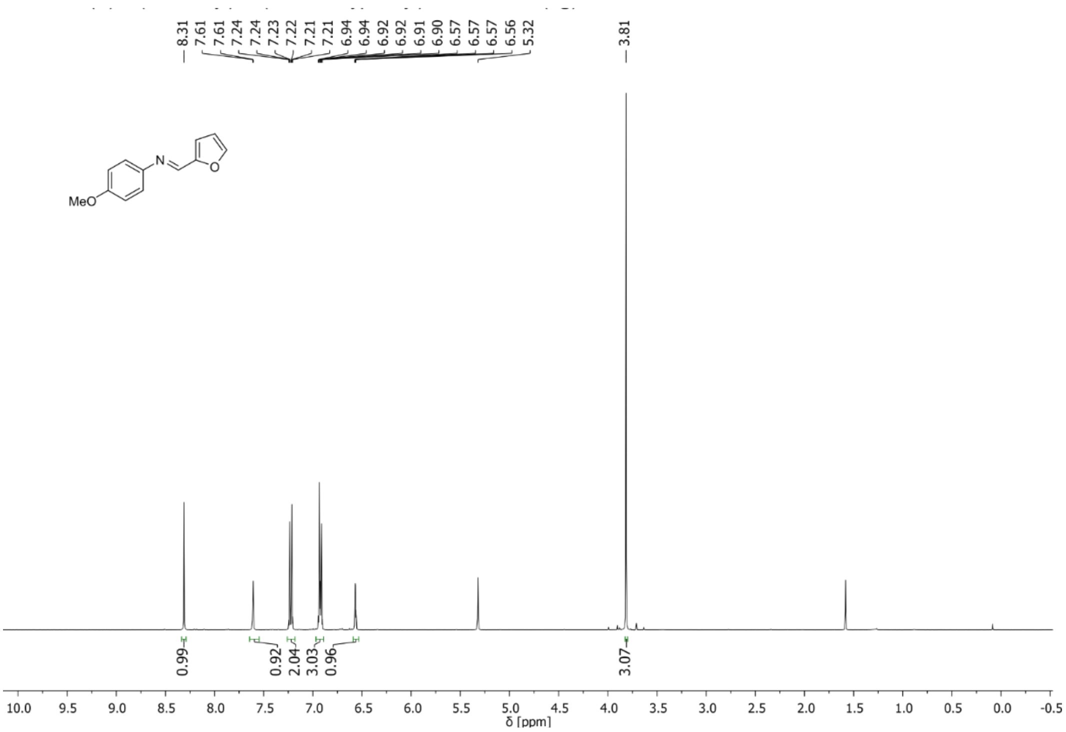

^{1}H NMR: (E)-1-(furan-2-yl)-N-(4-methoxyphenyl)methanimine (**1ag**)

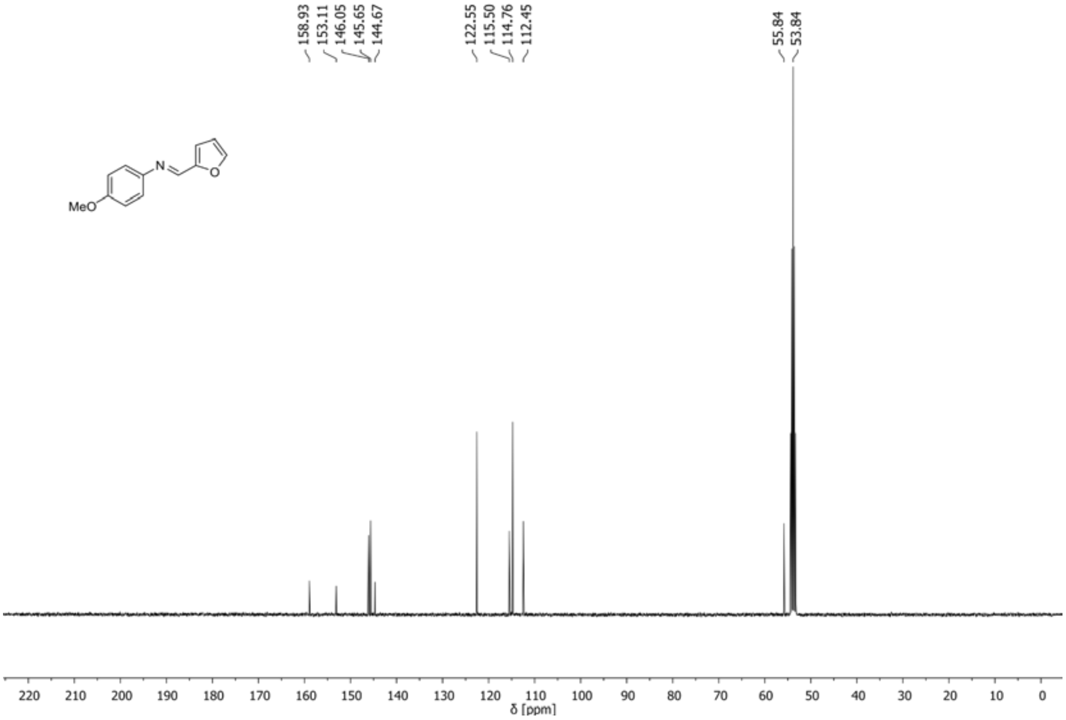

^{13}C{^{1}H} NMR: (E)-1-(furan-2-yl)-N-(4-methoxyphenyl)methanimine (**1ag**)

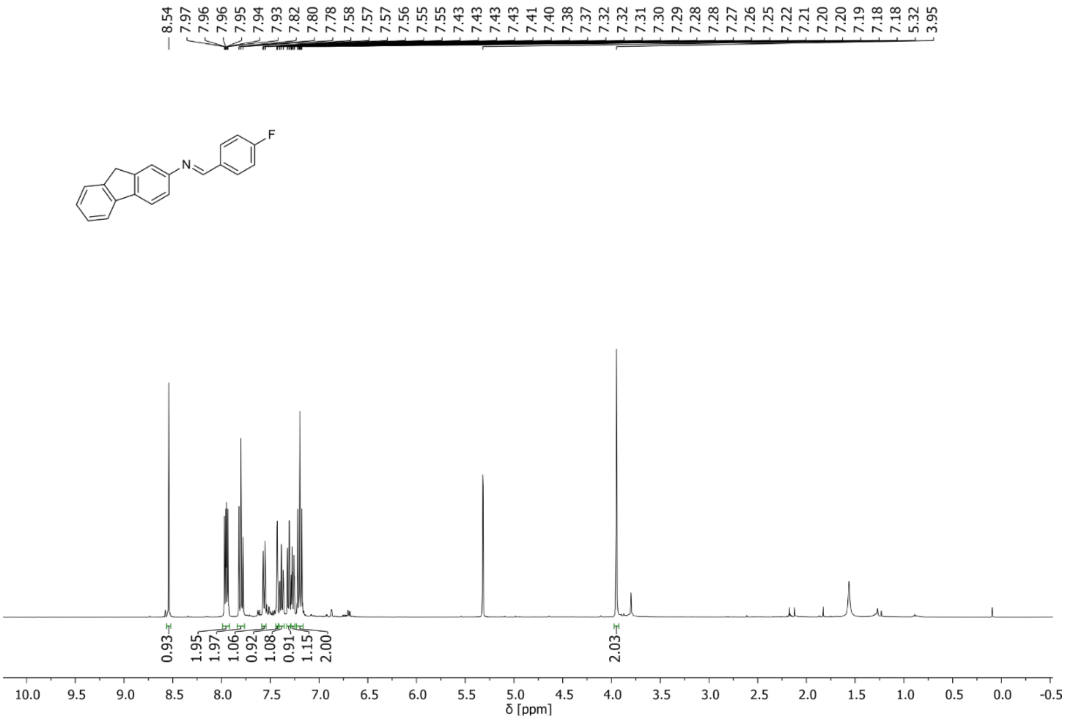

^{1}H NMR: (E)-N-(4-fluorobenzylidene)-9H-fluoren-2-amine (**1ah**)

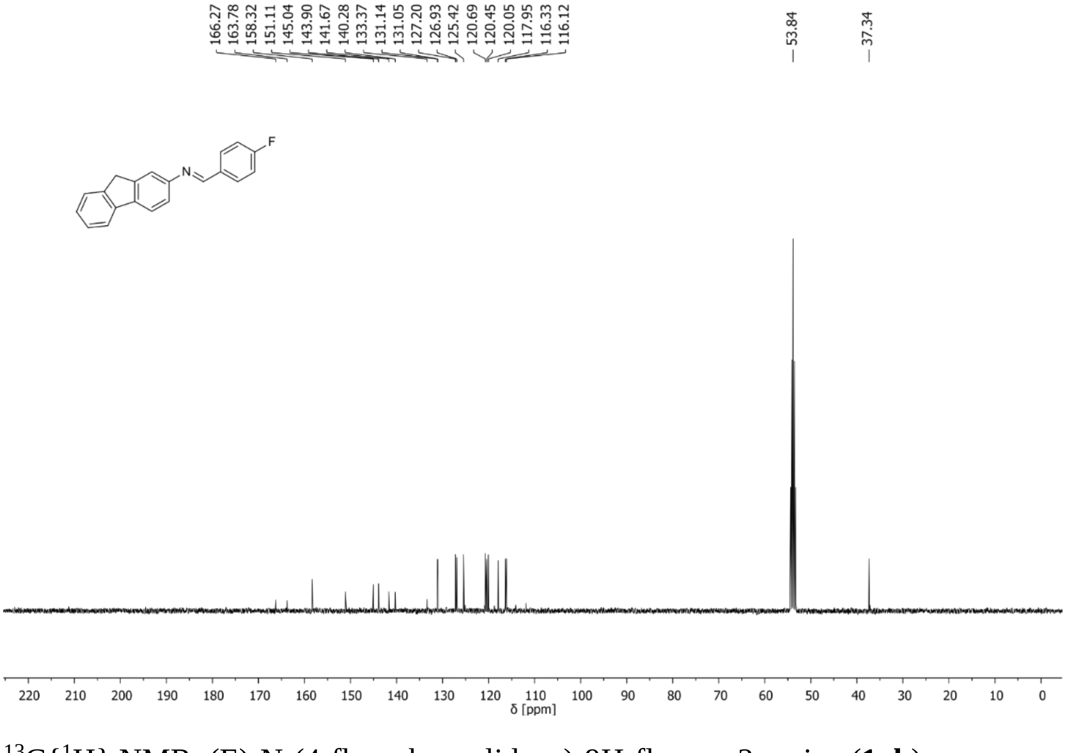

^{13}C{^{1}H} NMR: (E)-N-(4-fluorobenzylidene)-9H-fluoren-2-amine (**1ah**)

7. References

(1) H. Naka, D. Koseki, Y. Kondo, *Adv. Synth. Catal.* **2008**, *350*, 1901-1906.

(2) L. Tang, H. Sun, Y. Li, Z. Zha, Z. Wang, *Green Chem.* **2012**, *14*, 3423-3428.

(3) J. Wu, C. Darcel, *J. Org. Chem.* **2021**, *86*, 1023-1036.

(4) R. Fertig, T. Irrgang, F. Freitag, J. Zander, R. Kempe, *ACS Catal.* **2018,** *8*, 8525-8530.

(5) D. Riemer, W. Schilling, A.G. Götz, Y. Zhang, S. Gehrke, I. Tkach, O. Holloczki, *ACS Catal.* **2018**, *8*, 11679-11687.

(6) X. Hong, H. Wang, B. Liu, B. Xu, *Chem. Commun*. **2014**, *50*, 14129-14132.

(7) F.J. Goetz, *J. Heterocycl. Chem.* **1968**, *5*, 501-507.

(8) S.M. Landge, V. Atanassova, M. Thimmaiah, B. Torok, *Tetrahedron Lett.* **2007**, *48*, 5161-5164.

(9) A.L. Iglesias, J.J. Garcia, *J. Mol. Catal. A: Chem.* **2009**, *298*, 51-59.

(10) M. Mastalir, M. Glatz, N. Gorgas, B. Stöger, E. Pittenauer, G. Allmaier, L.F. Veiros, K. Kirchner, *Chem. Eur. J.* **2016**, *22*, 12316-12320.

(11) L.M. Kammer, M. Krumb, B. Spitzbarth, B. Lipp, J. Kuhlborn, J. Busold, O.M. Mulina, A.O. Terentev, T. Opatz, *Org. Lett.* **2020**, *22*, 3318-3322.

(12) B. Li, Y. Wang, Q. Chi, Z. Yuan, B. Liu, Z. Zhang, *New J. Chem*. **2021**, *45*, 4464-4471.

(13) N. Azizi, M. Edrisi, *Monatsh. Chem.* **2015**, *146***,** 1695-1698.

(14) G.J. Zhang, F.Y. Tang, X.Y. Wang, P. An, L.Q. Wang, Y.N. Liu, *ACS Sustainable Chem. Eng.* **2020**, *8*, 6118-6126.

(15) E. Manivannan, S.C. Chaturvedi, *Bioorg. Med. Chem.* **2012**, *20*, 7119-7127.

(16) F. Stanek, R. Pawlowski, P. Morawska, R. Bujok, M. Stodulski, *Org. Biomol. Chem.* **2020**, *18*, 2103-2112.

(17) L. Wang, B. Chen, L. Ren, H. Zhang, Y. Liu, S. Gao, *Chin. J. Catal.* **2015**, *36*, 19-23.

(18) A. Tejeria, Y. Perez-Pertejo, R.M. Reguera, R. Balaña-Fouce, C. Alonso, M. Fuertes, M. Gonzalez, G. Rubiales, F. Palacios. *Eur. J. Med. Chem.* **2016**, *124*, 740-749.

(19) W. Gong, M. Han, C. Chen, Y. Lin, G. Wang, H. Zhang, H. Zhao, *ChemCatChem* **2020**, *12*, 5948-5958.

(20) J.C. Anderson, G.P. Howell, R.M. Lawrence, C.S. Wilson, *J. Org. Chem.* **2005**, *70*, 5665-5670.

(21) D.J. Young, M.J.T. Robinson, *J. Labelled Cpd. Radiopham.* **2000**, *43*, 121-126.

(22) W. Nam, S.Y. Oh, M.H. Lim, M.H. Choi, S.Y. Han, G.J. Jhon, *Chem. Commun.* **2000**, 1787–1788.

(23) a) C. Adamo, V. Barone, *J. Chem. Phys.* **1999**, *110*, 6158-6170; b) F. Weigend, R. Ahlrichs, *Phys. Chem. Chem. Phys.* **2005**, *7*, 3297-3305; c) E. Caldeweyher, C. Bannwarth, S. Grimme, *J. Chem. Phys.* **2017**, *147*, 034112 d) V. Barone, M. Cossi, *J. Phys. Chem. A* **1998**, *102*, 1995-2001; e) F. Neese. *WIREs ComputMol Sci.* **2022**,*12*, e1606; f) F. Neese, F. Wennemohs, U. Becker, C. Riplinger. *J. Chem. Phys.* **2020**, *152*, 224108; g). C. Riplinger, F. Neese, *J. Chem. Phys.* **2013**, *138*, 034106; h) J. Zheng, X. Xu, D. G. Truhlar, *Theor. Chem. Acc.* **2011**, *128*, 295; i) F. Weigend, *Phys. Chem. Chem. Phys.* **2006**, *8*, 1057-1065; j) S. Grimme. *J. Chem. Phys.* **2022**, *124*, 034108.

(24) J. N. Harvey, M. Aschi, H. Schwarz, W. Koch. *Theor. Chem. Acc*. **1998**, *99*, 95.

(25) S. Stoll, A. Schweiger, *J. Magn. Reson.* **2006**, *178*, 42-55.
